# Understanding the emergence of the boson peak in molecular glasses

**DOI:** 10.1038/s41467-023-35878-6

**Published:** 2023-01-13

**Authors:** Mario González-Jiménez, Trent Barnard, Ben A. Russell, Nikita V. Tukachev, Uroš Javornik, Laure-Anne Hayes, Andrew J. Farrell, Sarah Guinane, Hans M. Senn, Andrew J. Smith, Martin Wilding, Gregor Mali, Motohiro Nakano, Yuji Miyazaki, Paul McMillan, Gabriele C. Sosso, Klaas Wynne

**Affiliations:** 1 School of Chemistry, University of Glasgow, Glasgow, UK; 2 Department of Chemistry, University of Warwick, Warwick, UK; 3 Slovenian NMR Centre, National Institute of Chemistry, Ljubljana, Slovenia; 4 Diamond Light Source, Harwell Science and Innovation Campus, Harwell, UK; 5 School of Chemistry, University of Cardiff, Cardiff, UK; 6 Department of Inorganic Chemistry and Technology, National Institute of Chemistry, Ljubljana, Slovenia; 7 Research Center for Thermal and Entropic Science, Osaka University, Osaka, Japan; 8 Department of Chemistry, University College London, London, UK

**Keywords:** Structure of solids and liquids, Structure of solids and liquids

## Abstract

A common feature of glasses is the “boson peak”, observed as an excess in the heat capacity over the crystal or as an additional peak in the terahertz vibrational spectrum. The microscopic origins of this peak are not well understood; the emergence of locally ordered structures has been put forward as a possible candidate. Here, we show that depolarised Raman scattering in liquids consisting of highly symmetric molecules can be used to isolate the boson peak, allowing its detailed observation from the liquid into the glass. The boson peak in the vibrational spectrum matches the excess heat capacity. As the boson peak intensifies on cooling, wide-angle x-ray scattering shows the simultaneous appearance of a pre-peak due to molecular clusters consisting of *circa* 20 molecules. Atomistic molecular dynamics simulations indicate that these are caused by over-coordinated molecules. These findings represent an essential step toward our understanding of the physics of vitrification.

## Abstract (web summary)

The ‘boson peak’ refers to an extra peak in the terahertz vibrational spectrum of glasses. It is now shown that for liquids of highly symmetric molecules the boson peak can be singled out by means of depolarized Raman scattering; the peak is linked to the formation of clusters of about 20 molecules.

## Introduction

Glasses are prepared by the rapid cooling of liquids (while avoiding crystal nucleation) and are therefore solids with some of the disorder of the liquid phase frozen in. When a liquid is (super) cooled, the primary or *α*-relaxation rate—directly related to viscous flow—decreases and decreases dramatically somewhere near the glass-transition temperature *T*_*g*_, where calorimetry measurements identify a rapid decrease in heat capacity^1^. Below the glass transition, secondary or *β*-relaxation processes can be observed due to faster dynamic processes that become uncoupled from the *α*-relaxation on vitrification and are the dominant relaxation channel in the glassy state^2^.

The molecular origin of these secondary relaxations is still unclear^3^. Because secondary relaxations were first observed in polymer glasses, they were assumed to originate in the small angle orientational diffusion of the side chains or functional groups of the polymers and therefore of essentially intramolecular character. However, the observation of secondary relaxations in rigid polymer glasses and even metallic glasses^2^ implies that at least some (now referred to as “Johari–Goldstein”) secondary relaxations are of an intermolecular character. One suggestion is that they are caused by spatial inhomogeneities^4^ giving rise to more loosely packed regions, distinct relaxing domains, defects^5,6^, or regions of different packing such as locally favoured *vs*. liquid-like structures^7^. Another approach starts from the liquid’s potential energy landscape and views secondary relaxations as transitions between neighbouring potential energy minima while primary relaxation corresponds to the higher energy transitions between mega basins^1^. This picture explains why *α* relaxation is frozen out before *β* relaxations.

Another phenomenon often associated with the glass is the boson peak^1,8^, which is observed as an excess intensity of low-frequency modes around about 1 THz in spectroscopic studies or as an excess heat capacity signature. The boson peak represents a peak in the vibrational density of states^9^ corresponding to an excess in the density of states over that expected from phonons in a perfect Debye crystal. This gives rise to anomalous behaviour of the low-temperature heat capacity *C*_*p*_ and a peak in *C*_*p*_/*T*^3^ at a few 10 s of K. The origin of the phenomenon has been assigned various interpretations, ranging from the occurrence of “two-level” excitations associated with broken bonds or other defects in glasses^10^, to other mechanisms for creating an excess in the low-frequency vibrational density of states in the terahertz range over that expected for dispersive phonons in a perfect crystal, giving rise to an additional contribution to the very low-temperature heat capacity *C*_*p*_ leading to a peak in *C*_*p*_/*T*^3^^11^. The presence of the boson peak has been linked to fluctuating elastic constants within a structurally disordered amorphous matrix^12^, quasi-localised soft potential defects^13,14^, localisation of transverse phonons associated with defective soft structures^15,16^, (quasi) localised vibrational modes of locally favoured structures^17,18^, a crystal-like van Hove singularity near the pseudo-Brillouin zone edge washed out by structural disorder^19^, may be caused by diffusive damping rather than spatial disorder^20^, and might not even contribute any extra heat capacity^21^.

Both Johari–Goldstein *β* relaxations and the boson peak are intermolecular in nature. Therefore, a study of the intermolecular structure and dynamics in supercooled and vitrified liquids could provide insight into these phenomena. Intermolecular modes have been observed using various techniques including inelastic neutron, X-ray, and Raman scattering as well as infrared and dielectric relaxation spectroscopy. However, in these techniques the intermolecular modes are typically (partially) obscured by other terahertz frequency bands, such as low-frequency vibrations, librations, and (above the glass transition) orientational and translational relaxation bands. This complicates lineshape analysis.

We have previously shown that symmetry can be used to simplify the depolarised Raman spectra of liquids in the terahertz range^22–24^. The orientational-relaxation and librational bands are proportional to the anisotropic part of the molecular polarisability tensor, which vanishes for molecules with tetrahedral or octahedral symmetry, leaving a simplified spectrum. The disadvantage of Raman spectroscopy is that the Raman coupling coefficient has a frequency dependence that is unknown in principle^25^ although it is experimentally found to be linearly proportional to frequency^26–29^. Inelastic neutron and X-ray scattering, on the other hand, are sensitive to the movements of all atoms and have no frequency-dependent coupling coefficient. However, the clear advantage of Raman spectroscopy is selectivity through symmetry and (in the technique used here) greater signal to noise over a wide frequency range. Thus, it would be enormously advantageous to study liquids of symmetric molecules that also readily vitrify into a glass. However, in practice this appears to be a contradiction in terms.

The alkoxides of early transition metals such as Ti and Zr and group 14 elements (Si, Ge, etc.) are important compounds in their liquid state, providing precursors for sol–gel and chemical vapour deposition technologies for the production of sols, gels and ceramics, including zeolites^30^, periodic mesoporous silicas^31^, organosilicas^32^ and materials with photocatalytic and superhydrophobic properties^33,34^. The transition metal alkoxides (M(OR)_n_) oligomerise while exhibiting rapid ligand exchange due to the labile nature of the M-O bonds and the ability of the metal centres to adopt a coordination higher than four^35^. In contrast, silicon-based alkoxides such as Si(OBu)_4_ (tetrabutyl orthosilicate, TBOS hereafter) do not exhibit ligand lability and do not normally oligomerise. All of these liquids are known to hydrolyse readily to form amorphous metal-oxide gels and organic–inorganic hybrid materials^36^.

Here, we show that TBOS is an example of a class of monomeric tetrahedrally symmetric molecular liquids that nonetheless vitrify. In fact, it was impossible to make these compounds crystallise at all. TBOS was studied with sub-terahertz depolarised Raman spectroscopy, synchrotron X-ray scattering, calorimetry, and atomistic molecular dynamics simulations to gain insights into the temperature-dependent relaxation processes and their dynamic signatures in relation to structural changes that occur within the liquid and glassy states. It was found that the depolarised Raman spectra are indeed greatly simplified due to the molecular symmetry. This allowed us to separate *α*-relaxation processes from non-diffusive modes, in particular intermolecular modes. Detailed temperature-dependent lineshape analysis of these intermolecular modes shows that in TBOS, they are inhomogeneously broadened and split into two clearly identifiable bands developing differently with temperature. The behaviour of the lower frequency of these two bands is consistent with that of the boson peak. Analysis of the first sharp diffraction peak in temperature-dependent small and wide-angle X-ray scattering experiments show inhomogeneous broadening and a weak pre-peak in the glass—corresponding to clusters about three TBOS molecules across—consistent with the OKE results. Molecular dynamics simulations reveal an increase in the average coordination number of TBOS molecules in the glass, which manifests itself as a change in the topology of the disordered network. In particular, by means of a Voronoi analysis, we pinpoint the emergence of specific over-coordinated molecular environments, unique to the glass. In addition, the topology of these Voronoi polyhedra and a bond orientational order analysis suggest that the over-coordination of the glass prevents the supercooled liquid (which displays a weak tendency toward HPC order) from crystallising, consistent with the experimental data. These topological features might also be responsible for the emergence of the pre-peak in the X-ray scattering experiments.

## Results

Tetrabutyl orthosilicate (TBOS) is a viscous liquid that does not crystallise and has a glass transition temperature *T*_*g*_ as measured by calorimetry (see Supplementary Fig. 1) of 120 K. The experimentally determined shear viscosity of TBOS is shown in Supplementary Fig. 2 together with a fit to the Vogel–Fulcher–Tammann equation with *T*_*0*_ = 105.4 ± 0.5 K.

Femtosecond optical Kerr-effect (OKE) spectroscopy^37,38^ was used to measure the Bose–Einstein corrected depolarised Raman spectrum using a time-resolved pump–probe technique and numerical Fourier deconvolution. In our set-up^23,24,39,40^, which has a time resolution of about 20 fs, the pump–probe delay can be as large as 4 ns resulting in spectra with a maximum spectral range from 125 MHz to 50 THz but is limited here to the range 10 GHz to ~10 THz to maximise the signal to noise.

The low-frequency depolarised Raman spectra of liquids typically contain (overlapping) contributions from orientational relaxation, translational relaxation, intermolecular cage rattling motions, librations, and vibrations^41,42^. We have shown that—at least within the accessible frequency range >1 GHz—the orientational and translational relaxations in nearly all liquids follow the Stokes–Einstein–Debye and Stokes–Einstein laws tracking the macroscopic shear viscosity and are therefore representative of primary or *α* relaxations^24^.

The amplitudes of orientational relaxation and librations in the spectra are proportional to the anisotropic molecular polarisability tensor, which vanishes in a molecule with tetrahedral, octahedral, or icosahedral symmetry. Quantum chemistry calculations were carried out on TBOS (see “Methods”) and stability calculations of dimers, trimers, and higher aggregates show that TBOS is expected to remain monomeric under normal conditions. The calculated infrared spectrum in the Si–O–C stretch region (around 1100 cm^−1^) matches the experimental one (see Supplementary Fig. 3), confirming that TBOS is monomeric and the silicon atom tetrahedrally coordinated as expected. Temperature-dependent ^13^C NMR spectroscopy from 20° to −50 °C shows four sharp bands as expected for a monomer (see Supplementary Fig. 4).

Calculation of the molecular polarisability tensor of 100 structures randomly picked from 31,500 low-energy conformers shows that the anisotropic polarisability remains an order of magnitude smaller than the isotropic one, demonstrating that the deviation from perfect tetrahedrality due to the flexibility of the butoxy side chains has a minimal effect. Therefore, the spectrum of TBOS should be greatly simplified due to symmetry, only showing translational relaxation, intermolecular cage rattling motions, and vibrations.

### The OKE spectra

**Fig. 1:**
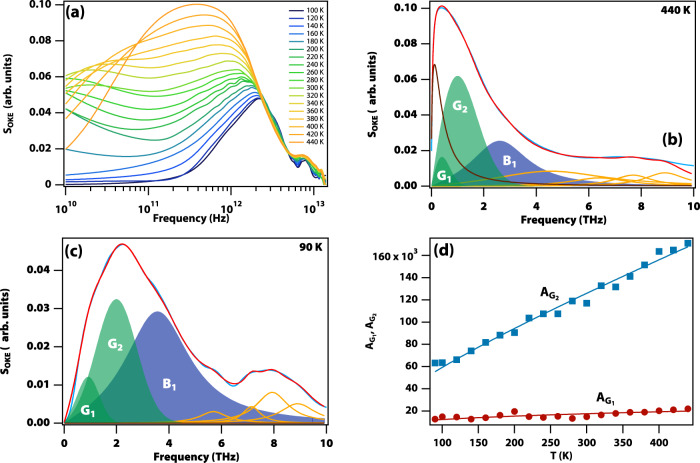
Optical Kerr-effect (OKE) spectra of supercooled and vitrified tetrabutyl orthosilicate (TBOS). **a** All data from 90 to 440 K. **b**, **c** Two representative temperatures and fits. The black line in (**b**) is the component due to diffusive *α* relaxation fitted to a Havriliak–Negami function, which freezes out below the glass transition and is therefore absent in (**c**). The two green bands at low frequency are intermolecular modes fitted to two Gaussian functions. The blue band is an intramolecular vibration fitted to a Brownian oscillator function with constant amplitude. The yellow curves are additional intramolecular vibrations. **d** Temperature-dependent amplitudes of the low-frequency (*A*_*G1*_, red disks) and high-frequency (*A*_*G2*_, blue squares) intermolecular modes. The lines are guides to the eye. While these amplitudes change, the amplitude of the higher frequency intramolecular modes remain unchanged with temperature as expected.

OKE spectra of TBOS were obtained over a temperature range from 90 to 440 K, as shown in Fig. 1 (see Supplementary Figs. 5 and 6 for individual spectra). The spectra are largely temperature independent ≥3 THz as this region only features intramolecular modes. The low-frequency (≤3 THz) part of the spectra has a strong temperature dependence with changes in shape as well as amplitude.

The spectra in this low-frequency range could be fitted consistently with four functions (see also Supplementary Note 1 for details and Supplementary Table 1 for fit parameters): a Havriliak–Negami function representing the diffusive *α* relaxation, two Gaussian functions representing the intermolecular modes, and a Brownian oscillator function for the lowest frequency intramolecular mode. As expected, the diffusive *α* relaxation is strongly temperature-dependent and freezes out below the glass transition. An Arrhenius plot of the relaxation time constant is shown in Supplementary Fig. 7, showing that it closely follows the temperature-dependent macroscopic shear viscosity through the Stokes–Einstein law, as expected for translational relaxation, and demonstrating it is an *α* relaxation. The Brownian oscillator has a constant amplitude consistent with an intramolecular mode.

**Fig. 2:**
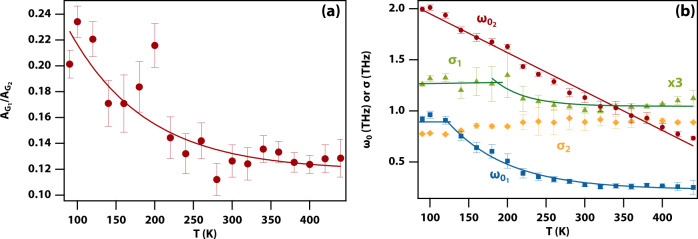
Temperature dependence of the fit parameters for the intermolecular modes. **a** Ratio of the amplitude of the low-frequency intermolecular mode over that of the high-frequency one. The solid red line is an exponential fit to guide the eye (The data point at 200 K was omitted in this fit on account of the noise at low frequencies in the corresponding OKE data). **b** Centre frequency *ω*_0_ of the two intermolecular modes (blue squares and red disks, also shown are linear and exponential fits to guide the eye) and the corresponding widths *σ* (green triangles and yellow diamonds respectively, also shown are an exponential fit and a horizontal line to guide the eye).

The amplitudes of the two Gaussians (see Fig. 1) are proportional to temperature, showing that they are collision-induced intermolecular “cage rattling” modes. The spectra below the glass transition (e.g., at 90 K in Fig. 1) clearly show the two intermolecular bands as a peak at 2 THz and a shoulder at 0.9 THz. As can be seen in Fig. 2, the two intermolecular bands evolve differently as a function of temperature. The ratio of the amplitudes of the low and high-frequency bands stays approximately constant at high temperature but doubles on cooling to the glass transition. The width of the high-frequency band is essentially temperature independent suggesting the corresponding inhomogeneity is constant. The width of the low-frequency band slightly increases on cooling and plateaus below ~200 K but this is a minor effect. The centre frequency of both bands increases on cooling in a linear fashion for the high-frequency and a nonlinear fashion for the low-frequency band, the latter plateauing below the glass transition.

### WAXS and Raman experiments

**Fig. 3:**
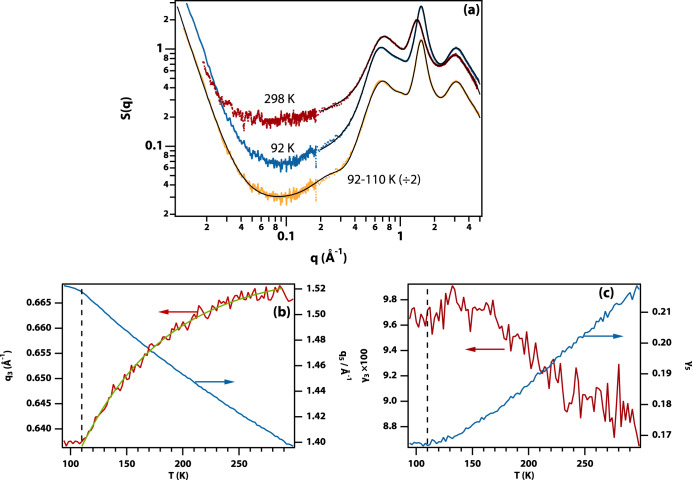
Analysis of temperature-dependent WAXS data. **a** Experimental SAXS and WAXS data taken at 298 K (red) and 92 K (blue) and fit to four Gaussians and a Lorentzian (black). The average of the nine data sets at 110 K and below is shown (yellow) with a fit including an additional Gaussian to account for the pre-peak (black). **b** Variation of the peak of the first (*q*_3_, red) and second (*q*_5_, blue) sharp diffraction peaks in the WAXS data obtained from fits to a Gaussian and a Lorentzian, respectively. The green line is an exponential fit to guide the eye. **c** Variation of the width of the first (*γ*_3_, red) and second (*γ*_5_, blue) sharp diffraction peaks.

Structural information can be obtained using small (SAXS) and wide-angle X-ray scattering (WAXS) experiments carried out over a similar temperature range as the OKE experiments with a 2 K step size (see Fig. 3 and Supplementary Fig. 8). The first sharp diffraction peak at ~0.65 Å^−1^ is consistent with the Si–Si nearest neighbour distance of 10.4 Å calculated from the liquid density (see Supplementary Fig. 9).

The SAXS/WAXS intensity data were analysed by curve fitting, requiring a Gaussian function to fit the first sharp diffraction peak and a Lorentzian for the second (see Supplementary Note 2 and Supplementary Table 2 for the fit parameters). The fits were used to calculate the radial distribution functions using analytical transformation, showing a reduction of the first and second solvation shell radius on cooling as expected (see Supplementary Fig. 10).

Figure 3b, c shows the evolution of the peak position and width of the first and second sharp diffraction peak as a function of temperature. The first sharp diffraction peak shifts in a nonlinear fashion to lower *q* on cooling, while the second peak linearly shifts to higher *q*. Both show a distinct change in their evolution at 110 K, which is slightly below the glass transition temperature as determined using calorimetry (124 K). Temperature-dependent Raman spectra of the CH-stretch band (Supplementary Fig. 11) show that, on lowering the temperature, the TBOS molecules reduce the number of gauche defects in the alkoxide side chains, consistent with the lowering of the peak position of the first sharp diffraction peak on cooling.

Close inspection of the data at the lowest temperatures shows the presence of a weak pre-peak at q ~0.2 Å^−1^, however, the signal-to-noise in the SAXS–WAXS transition region is insufficient for full temperature-dependent analysis of this feature. The nine lowest temperature data sets (≤110 K) were averaged for improved signal-to-noise revealing a clear pre-peak. This spectrum could be fitted with an additional Gaussian, allowing a determination of the pre-peak position as 0.234 Å^−1^ (see Supplementary Table 3 for fit parameters).

### MD simulations

**Fig. 4:**
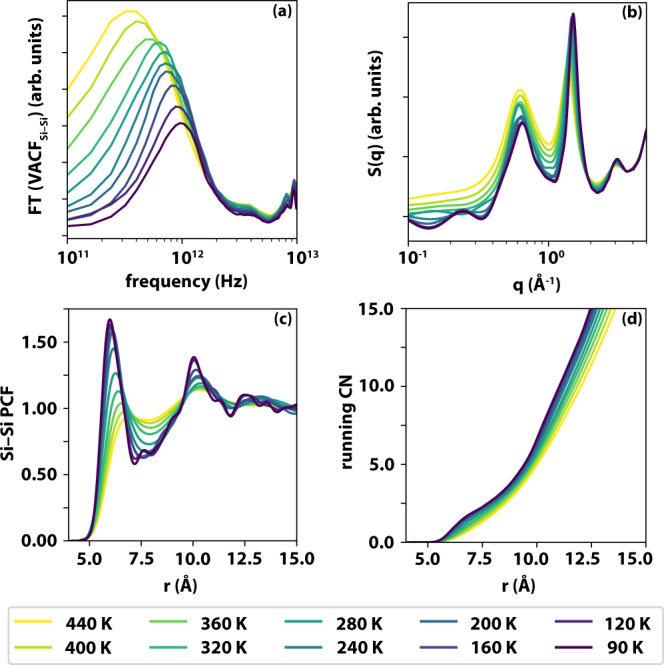
Results of the temperature-dependent molecular dynamics simulations of TBOS. **a** Power spectra obtained from the Fourier transform of the Si–Si velocity–velocity autocorrelation functions. **b** Static structure factor, including Si, O and C atoms. **c** Si–Si pair correlation functions. **d** running Si–Si coordination number.

Atomistic MD simulations (see “Methods” and Supplementary Note 3 for details) were used to generate a 512-molecule model of liquid TBOS, quenched from 440 K to 90 K at a rate of 7.5 × 10^8 ^K/s into the glass. The resulting *T*_*g*_ is 249 ± 20 K (see Supplementary Fig. 12), significantly higher than the experimental value (124 K) due to the much faster cooling rate. The Fourier transform of the Si–Si velocity–velocity autocorrelation function (Fig. 4a), which thanks to the molecular symmetry of TBOS should display the same features of the OKE spectra, indeed shows a decrease of signal intensity and a shift to higher frequencies on cooling. Computation of the total Si–Si (static) structure factor (Fig. 4b) shows the emergence of a low-*q* feature on cooling between 0.25 and 0.45 Å^−1^_,_ consistent with the pre-peak observed experimentally (Fig. 3a). The occurrence of this structural feature was verified by generating three additional models of TBOS, see Supplementary Fig. 13.

The Si–Si pair correlation function (Fig. 4c) shows a significant increase in short-range order, particularly in the second coordination shell. The first coordination shell (up to ~8 Å, see Fig. 4d) only contains, on average, about 2.5 molecules, showing a lack of tetrahedral order in both liquid and glassy TBOS. However, the second coordination shell (up to ~12 Å) contains about 12 molecules.

**Fig. 5:**
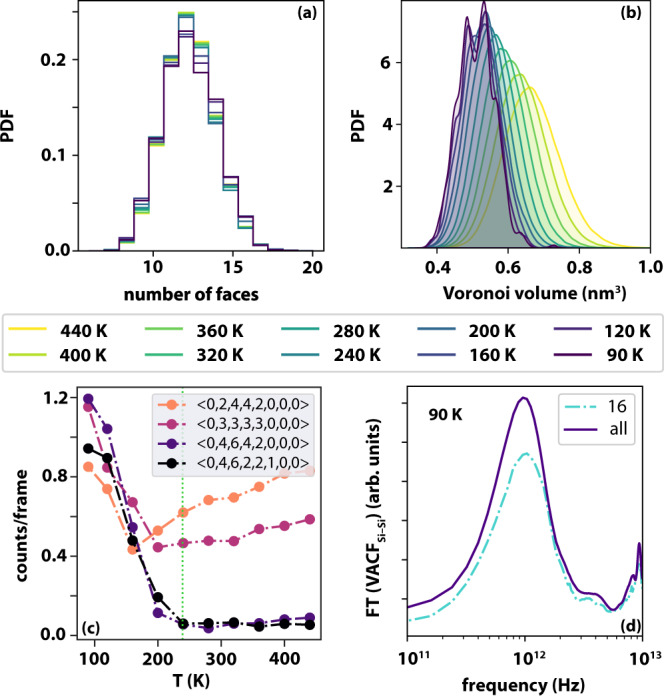
Temperature-dependent Voronoi analysis of TBOS models. **a** Probability density function of the number of faces characterising the Voronoi polyhedra (VP) for each Si atom, averaged over 1000 frames across a 10 ns long MD trajectory. **b** Probability density function of the volume of the VP. **c** Frequency of the occurrence of selected VP as a function of temperature. See Supplementary Table 4 for information about the indices of each VP. The green dotted line indicated the value of (computationally obtained) *T*_*g*_. **d** Power spectra obtained from the Fourier transform of the Si–Si velocity–velocity autocorrelation functions at 90 K. The purple and light blue lines refer to the result obtained considering all the Si atoms (same as Fig. 4a) and those Si atoms characterised by VP with 16 faces only, respectively.

A Voronoi analysis using the Si atoms was performed (Fig. 5a) to gain further insight into these structural changes and to provide an effective coordination number. Overall, it is clear that the network of Si atoms is predominantly 12-coordinate at any given temperature. However, below *T*_*g*_, the probability density of the volumes of the Voronoi polyhedra (VP) splits (Fig. 5b), indicating the emergence of specific structural features. These can be identified (Fig. 5c) as VP with 15 and 16 faces, which are unique to the glassy state. VP characteristic of ordered phases (see Fig. 5c and Supplementary Table 4) are very infrequently observed. VP characteristics of FCC order, such as the <0, 3, 6, 4 >VP, are found in the supercooled liquid but disappear below *T*_*g*_, where the <0, 4, 4, 2 >VP, characteristic of HCP order, makes an appearance. This is consistent with both the local environment analysis and the bond-orientation order analysis (summarised in Supplementary Fig. 14), which show that TBOS displays a weak tendency toward HPC order—which however is frustrated by the overcoordinated, largely disordered network emerging in the proximity of *T*_*g*_. In fact, the overcoordinated VP tend to form larger clusters as the liquid is cooled below *T*_*g*_, as shown in Supplementary Fig. 15.

The analysis of the orientation of TBOS molecules as a function of temperature (Supplementary Fig. 16), indicates that TBOS is perfectly isotropic even below *T*_*g*_ and that the degree of orientational order in the supercooled liquid is higher than that observed for the glass. The simulations also reproduce the reduction of the number of gauche defects in the alkoxide side chains on cooling (Supplementary Fig. 17).

Below *T*_*g*_ the self-diffusion of the system is so slow that it is possible to relate the power spectrum of the system to specific polyhedra. It is found that the high-frequency region of the power spectra is mostly linked to the polyhedra with 16 faces (Fig. 5d) at 90 K. Thus, the increase in the local coordination may be part responsible for the blueshift of the OKE spectra on cooling.

### Low-temperature calorimetry

Supplementary Fig. 18 shows the specific heat and its Debye representation *C*_*p*_/*T*^3^ measured using adiabatic and relaxation calorimetry (see “Methods”). The specific heat curves exhibit a maximum in the *C*_*p*_/*T*^3^ representation at 6 K consistent with the boson peak.

## Discussion

Because of the near tetrahedral symmetry of TBOS and hence the nearly isotropic molecular polarisability, no orientational relaxation or librational motions are observed in the OKE spectra. This does not mean that the molecules do not orientationally relax or librate but simply that these processes are not observed here. The lowest frequency relaxational component in the spectra is therefore due to translational relaxation (in as far as rotational and translational motions can be disentangled) and becomes visible in the spectra through a collision-induced process^22–24^.

Apart from the translational relaxation, there are no further relaxational processes observed in TBOS, suggesting an absence of Johari–Goldstein *β* relaxations. However, the lowest observable frequency in these OKE experiments is 10 GHz, whereas *β* relaxations are typically observed near the glass transition and at much lower (~1 kHz) frequencies^2,3^. Therefore, the presence of *β* relaxations in TBOS cannot be ruled out. Similarly, a breakdown of the Stokes–Einstein law for translational relaxation much nearer the glass transition could also not be ruled out.

Because of the near isotropic polarisability tensor of TBOS, the intermolecular band is also collision-induced^22–24^. This band is therefore sensitive to transverse and longitudinal motions involving neighbouring molecules and hence to acoustic phonon-like modes near the pseudo-Brillouin-zone edge. Therefore, it is tempting to assign the two bands to the LA and TA phonons at the zone edge. However, these would be expected to have similar frequencies (as opposed to a ratio of ~2) and amplitudes (as opposed to a ratio of ~8), while evolving similarly as a function of temperature^22–24^. Therefore, the data are consistent with one of the two bands originating in acoustic phonons at the zone edge with the other one having a different origin.

The absolute amplitude of the intermolecular modes has limited physical relevance as these bands are collision-induced and their amplitude therefore reflects the (isotropic) molecular polarisability involved in making them visible through the dipole-induced-dipole effect^24,43^. However, the (temperature-dependent) amplitude *ratio* is physically relevant and shows that the low-frequency mode doubles in relative strength on cooling TBOS towards the glass transition. This behaviour is what one might expect for a boson peak, which reflects the additional vibrational density of states due to disorder that is frozen in during vitrification^1^. Hence, we assign the low-frequency intermolecular band as the boson peak and the high-frequency band as the acoustic phonon band at the pseudo-Brillouin zone edge.

Both intermolecular modes in TBOS are inhomogeneously broadened into Gaussian lineshapes. The width of the acoustic phonon band shows no temperature dependence consistent with it representing the inhomogeneity of the liquid state. The width of the boson peak increases on cooling (down to ~200 K) representing an increase in the inhomogeneity on cooling. It may be surmised that the boson peak’s lower (collision-induced) amplitude, lower frequency, and more rapidly changing frequency and width, are all associated with the formation of clusters of TBOS molecules undergoing collective oscillation.

The first sharp diffraction peak in the WAXS data is inhomogeneously broadened to a Gaussian consistent with the shape of the acoustic phonon band seen in OKE. At temperatures below the glass transition a pre-peak emerges with a peak at 0.234 Å^−1^ corresponding to a distance of 26.9 Å. This demonstrates the presence of supramolecular clusters in the glass ~2.7 TBOS molecules across or about 20 molecules in total. Although there is no incontrovertible proof, it is reasonable to conclude that this pre-peak has the same origin as the boson peak in the OKE spectra.

The atomistic MD simulations also reveal a low-q peak at 0.25 Å^−1^, while reproducing the key features of the OKE spectra and WAXS data. While it is not possible to make a quantitative connection with the supramolecular structures associated with the boson peak identified by OKE measurements (even slower cooling rates and even larger models would be needed), the MD simulations show that these structures are clusters of overcoordinated TBOS molecules which—were it not for the presence of the long side chains on top of the tetrahedral Si—would tend toward crystalline structural order.

The specific heat in its Debye representation *C*_*p*_/*T*^3^ shows a peak at 6 K. This shape can be reproduced by modelling the heat capacity of the crystal lattice with a Debye function (Debye temperature 60 K), Einstein functions for each of the 165 normal modes, and an additional mode with a frequency of 0.6 THz. This is consistent with the boson peak observed here at 0.9 THz without requiring a fudge factor^11^. However, in the absence of heat capacity data for the crystal, it cannot be ruled out that there might other sources such as hindered methyl-group rotations.

In summary, we have shown here that symmetry can be used to simplify depolarised Raman spectra to bring out the intermolecular modes. In TBOS, one of these modes is fully consistent with a boson peak, which is present throughout the (supercooled) liquid range but becomes stronger and more easily visible on vitrification. Molecular dynamics simulations and WAXS data show that the boson peak feature is related to clusters of overcoordinated TBOS molecules consisting of circa 20 molecules that are prevented from attaining crystalline structure. It was recently shown using a 2D computational model that such dynamical defects are a common structural origin of the boson peak^16^.

TBOS is representative of a broad class of molecular liquids consisting of alkoxides of early transition metals and group 14 elements of technological significance in which symmetry can be utilised to simplify Raman and infrared spectra while showing the full range of complexity of glass-forming liquids and crystal nucleation. By changing the metal or the ligand, the symmetry and strength of intermolecular interaction can be changed freely making these liquids of enormous interest for fundamental studies of nucleation and vitrification. Thus, this opens the way to the investigation of the detailed changes in the behaviour of boson peak as a function of temperature, pressure, fragility, and other physicochemical parameters, greatly aiding the science of the glassy state.

## Methods

### Sample preparation

TBOS purchased from Aldrich Chemistry and has a rated purity ≥97% and was used without further purification. Liquid samples were filtered with a PTFE filter (Millex) with 20-μm pore size and degassed for 1 min in an ultrasound bath before measurements. Room-temperature samples were contained in a 1-mm-thick rectangular quartz cuvette (Starna) and held in a temperature-controlled (±0.5 K) aluminium block. Low-temperature measurements were performed using a liquid-N_2_ cryostat (Oxford Instruments, ±0.1 K) in a nitrogen environment to avoid water condensation.

### OKE experimental details

A laser oscillator (Coherent Micra) produced ∼10 nJ pulses at a repetition rate of 82 MHz and with 800 nm nominal wavelength providing 20 fs temporal pulse width in the sample, broadening to 25 fs when using a cryostat. The OKE data were recorded in a standard time-domain pump–probe configuration and Fourier transformed to obtain the frequency-domain reduced depolarised Raman spectrum as described previously^44^. The data were analysed through curve fitting as described in Supplementary Note 1.

### SAXS and WAXS experiments

Small and wide-angle X-ray scattering (SAXS/WAXS) experiments were carried out at the Diamond Light Source (Oxford) at beamline I22 using Pilatus P3-2M (SAXS) and Pilatus P3-2M-L (WAXS) detectors and using an X-ray wavelength of 1 Å^45^. Liquid samples were contained in sealed polycarbonate capillaries and inserted into a Linkam capillary stage using mica windows allowing the temperature to be controlled between −180 °C and +550 °C with an accuracy of ±0.1 °C. The experiments were carried out by ramping down the temperature at a rate of 10 K/min and taking SAXS/WAXS data at 2 K intervals (400 ms averaging time).

The background SAXS/WAXS signal from the capillary, air, and vacuum window was subtracted and the data processed using Dawn to give the intensity *I*(*q*) of the scattered X-rays. The experimental structure factor was calculated using^46^

1 $$S(q)=\frac{I(q)}{{f}^{2}(q)}={S}_{coh}(q)+{f}^{2}(q){S}_{incoh}(q)$$

where the atomic structure factor *f*(*q*) was approximated using Gaussian functions with tabulated parameters^47^. The incoherent scattering contribution was then calculated^48^ and its amplitude determined by fitting *S*(*q*), thereby avoiding having to use the Krogh–Moe–Norman method^46^. However, it was found that the incoherent contribution was negligible in the range studied and hence it was ignored in order to improve the stability of the fitting procedure.

The SAXS data were smoothed with a 20-point moving average to improve the S/N at high *q*. All the SAXS data were then divided by 37.0 in order to overlap it with the WAXS data in the range *q* = 0.157 to 0.182 Å^−1^. The data were analysed through curve fitting as described in Supplementary Note 2.

### Molecular dynamics simulations

The all-atom CHARMM36 force field^49^ (version jul2017) was used to model the TBOS molecules. The relevant parametrisation was obtained via CGenFF^50^. Despite the lack of explicit refinement of the resulting force-field parameters, the computational results are in good agreement with the experimental measurements with respect to both structural and dynamical properties (see Figs. 4 and  5).

The GROMACS package (version 2021)^51^ was used to perform molecular dynamics simulations within the NPT ensemble. A leap-frog algorithm^52^ was used to integrate Newton’s equations of motion with a 2-fs time step. A twin cut-off of 12 Å was used for both electrostatic and van der Waals interactions, where, for the latter, forces were smoothly switched to zero between 10 and 12 Å. The Bussi–Donadio–Parrinello thermostat^53^ was used in conjunction with the Berendsen barostat^54^ to sample the isobaric-isothermal ensemble. The coupling constants for the thermostat and barostat are 1.0 and 4.0 ps, respectively. The LINCS^55^ algorithm was used to constrain the TBOS bonds involving hydrogen atoms.

In order to generate models of TBOS at different temperatures, 512 TBOS molecules were positioned at random positions within a cubic box. The system was then slowly equilibrated in terms of both temperature and pressure at 440 K and 1 bar. Under these conditions, the self-diffusion coefficient of the Si atoms was computed to be ~1 nm^2^/ns, which is indicative of a highly diffusive hydrodynamic regime. As the system was equilibrated for a further 20 ns at that temperature and pressure, statistically independent, well-equilibrated configurations of the liquid were generated, thus providing adequate starting points for the cooling ramp. The latter was implemented as a linear ramp, from 440 K to 90 K at 7.5 × 10^8 ^K/s. Configurations generated at temperature intervals of 40 K along the ramp were used as starting points for a 10-ns equilibration at that particular temperature. The system was also annealed from 90 K to 440 K in the same fashion, obtaining a discrepancy in the *T*_*g*_ of only 7 K. The lack of significant hysteresis is a strong indicator of the adequacy of the computational protocol.

The static structure factors reported in Fig. 4b were obtained according to widely used expression reported in, e.g., Eq. (6) in ref. ^56^. The X-ray atomic form factors were constructed from the relevant empirical constants^56^ that can be found in the International Tables for Crystallography^47^.

The power spectra reported in Figs. 4 and 5 were obtained from 10 ps (sampled every 2 fs) sections of MD trajectory taken from the 10 ns long equilibration runs at each temperature.

To perform the Voronoi analysis, the VORO + + library^57^ as well as Ovito^58^ were used.

### Differential scanning calorimetry

Differential scanning calorimetry measurements were carried out with a TA Instruments DSC 2500 differential scanning calorimeter equipped with a Quench Cooling Accessory. The samples were cooled from 40 °C with liquid nitrogen in circa 16 min to a temperature of −165 °C.

### Low-temperature calorimetry

Measurements of the specific heat, *C*_*p*_, from 1 K up to room temperature were taken. The measurements were carried out with a Quantum Design physical properties measurement system (PPMS) relaxation-type calorimeter employing a ^3^He probe in the temperature range 1 K ≤ T ≤ 20 K. In addition, specific heat measurements were performed from 10 to 300 K using an adiabatic calorimeter^59^. Resolutions of the measurements are 0.3% for the relaxation calorimetry and 0.2% for the adiabatic calorimetry. The sample masses used were 0.94217 g in the adiabatic measurement and 2.0799 mg in the PPMS instrument.

### FTIR

Fourier-transform infrared (FTIR) spectroscopy measurements used a Bruker Vertex 70 spectrometer purged with dry air. liquid samples were sandwiched between two ZnSe windows. Sub-ambient FTIR experiments were performed using the same liquid-N2 cryostat with ZnSe windows.

### NMR

Temperature-dependent ^13^C magic-angle spinning (MAS) NMR spectra were recorded on a 400 MHz Bruker Avance Neo spectrometer equipped with a 4-mm Bruker CPMAS probe. The investigated liquids were closed into Kel-F inserts before being put into 4-mm zirconia rotors. During the measurements, the samples were spun with frequencies of up to 10 kHz. A 90° pulse of 3.8 μs was used for the excitation of carbon nuclei, and proton decoupling was employed during signal acquisition. The number of scans was 256 using a repetition delay of 3 s. The ^13^C shifts were reported relative to the position of the ^13^C signal of tetramethylsilane (TMS).

### Raman

Confocal Raman microscopy experiments were performed using a Horiba LabRAM HR confocal microscope system. The excitation source was a linearly polarised 28-mW frequency-doubled DPSS laser operating at 532 nm. The temperature was controlled to ±0.1 K using a Linkam THMS600 microscope stage.

### Viscometry

Viscosity measurements were performed on an Anton Paar MCR 702e rheometer equipped with a CTD 600 MDR convection temperature device and using a 50-mm cone (cone angle =  1°, gap = 0.101 mm) in rotational shear mode. The temperature was lowered from 30 °C to −160 °C at a rate of 1 °C/min and the data recorded every minute.

### Molecular and normal mode calculations

While full conformational analysis of the TBOS molecule is beyond the scope of this study, it is still useful to estimate differences in dipole moment and polarisability for various conformers. In order to achieve that, low-energy conformers were generated using Confab algorithm^60^ yielding more than 31,500 conformers. 100 structures were then picked randomly to form a subset for subsequent DFT optimisations and property calculations.

All DFT calculations were performed using the ORCA 4.2.1 quantum chemistry programme^61^. Geometry optimisations and vibrational spectra calculations were carried out with the ωB97X-D3 exchange-correlation functional^62^ combined with ma-def2-SVP basis set^63^. Solvent effects were not accounted for. RIJCOSX approximation with default cut-offs was used for all DFT calculations. IR and depolarised Raman spectra for separate conformers were obtained in the double-harmonic approximation. Spectra were broadened with Lorentzians with 12 cm^−1^ half-width and averaged using Boltzmann weights.

**Fig. 6:**
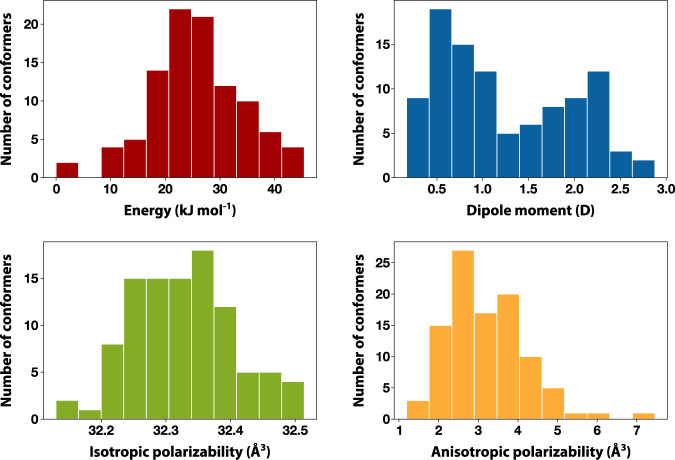
Histograms of various physicochemical properties of conformers of TBOS calculated using DFT. Shown are the distributions of the relative energy of each conformer, their permanent dipole moment, and (an)isotropic polarizabilities estimated using 100 TBOS conformers.

For all 100 conformers polarisability tensors were computed and a mean and standard deviation for iso- and anisotropic polarisabilities as well as dipole moment were estimated (see Fig. 6 and Table 1). It is worth noting that mean and standard deviation are unweighted, that is, each conformer contributed equally, which partially accounts for the relatively small sample size. For the anisotropic polarisability, the values are approximately an order of magnitude smaller than for the isotropic polarisability. Also, the relative SDs are comparable and large for the dipole moment and anisotropic polarisability as both are very sensitive towards changes in dihedrals for this molecule.

**Table. 1:** Dipole moment and polarisability statistics for TBOS conformers (mean and standard deviation)

μ / D	α_iso_, Å^3^	α_aniso_, Å^3^
1.25±0.71	32.3±0.1	3.2±1.0

### Silicon alkoxide aggregation

**Fig. 7:**
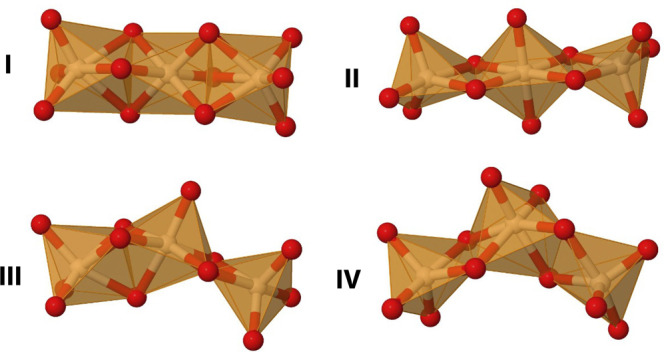
SiO skeletons for different energy minima. The SiO skeletons for the four lowest energy minima on the Si3(OMe)12 potential energy surface based on DFT calculations.

Recent work has shown that in some cases organosilicates can aggregate to form molecular structures with an octahedral SiO_6_ motif^64,65^. To ensure that simple silicon alkoxides remain monomeric under normal conditions, the stability of a dimer and various trimers (see Fig. 7) were investigated by means of DFT calculations for prototypical Si(OMe)_4_. It was found that, with the exception of the dimer and trimer **II**, the formation of aggregates is enthalpically favourable; however, due to the decrease of entropy associated with forming a cluster, the Gibbs free energies are clearly positive for all structures considered (Table 2). Since the formation of higher-order aggregates would impose even larger entropy penalty, silicon alkoxides can be assumed to remain monomeric under normal conditions.

**Table. 2:** Formation energies (in kJ/mol, per 1 mole of monomer) for the dimer and various trimers (**I**–**IV**) of Si(OMe)_4_

	Dimer	Trimers			
		I	II	III	IV
ΔE	4	−13	9	−6	−22
ΔH_298_	5	−11	13	−3	−18
ΔG_298_	39	39	64	45	32

## Supplementary information

Supplementary Information

Peer Review File

## Data Availability

The experimental data that support the findings of this study are available in the Enlighten Research Data Repository (University of Glasgow) with the identifier 10.5525/gla.researchdata.1381. The atomistic molecular dynamics data that support the findings of this study are available in the University of Warwick open access research repository—WRAP with the identifier http://wrap.warwick.ac.uk/172439/.
