# Author Correction: Understanding the emergence of the boson peak in molecular glasses

**DOI:** 10.1038/s41467-023-36662-2

**Published:** 2023-02-16

**Authors:** Mario González-Jiménez, Trent Barnard, Ben A. Russell, Nikita V. Tukachev, Uroš Javornik, Laure-Anne Hayes, Andrew J. Farrell, Sarah Guinane, Hans M. Senn, Andrew J. Smith, Martin Wilding, Gregor Mali, Motohiro Nakano, Yuji Miyazaki, Paul McMillan, Gabriele C. Sosso, Klaas Wynne

**Affiliations:** 1 School of Chemistry, University of Glasgow, Glasgow, UK; 2 Department of Chemistry, University of Warwick, Warwick, UK; 3 Slovenian NMR Centre, National Institute of Chemistry, Ljubljana, Slovenia; 4 Diamond Light Source, Harwell Science and Innovation Campus, Harwell, UK; 5 School of Chemistry, University of Cardiff, Cardiff, UK; 6 Department of Inorganic Chemistry and Technology, National Institute of Chemistry, Ljubljana, Slovenia; 7 Research Center for Thermal and Entropic Science, Osaka University, Osaka, Japan; 8 Department of Chemistry, University College London, London, UK

**Keywords:** Structure of solids and liquids, Structure of solids and liquids

Correction to: *Nature Communications* 10.1038/s41467-023-35878-6, published online 13 January 2023

The original version of this Article contained an error in Fig. 3, in which the panel 3b was incorrectly displayed. This correct version of Fig. 3b is:


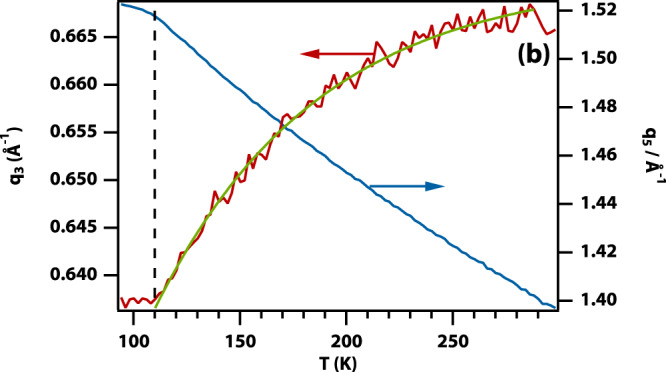


which replaces the previous incorrect version:


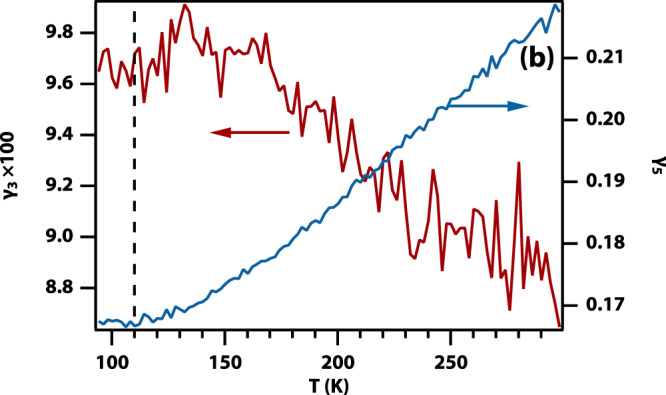


This has been corrected in both the PDF and HTML versions of the Article.

