# Supplementary material for: Understanding the emergence of the boson peak in molecular glasses
Source: Nat Commun. 2023 Jan 13;14:215. doi: 10.1038/s41467-023-35878-6 (PMC9839737; doi:10.1038/s41467-023-35878-6)
Supplement: Supplementary file 1 — Supplementary Information [file 41467_2023_35878_MOESM1_ESM.pdf]

Supplementary information
Understanding the emergence of the boson peak in molecular glasses

Mario González-Jiménez, Trent Barnard, Ben A. Russell, Nikita V. Tukachev, Uroš Javornik, Laure-Anne Hayes, Andrew J. Farrell, Sarah Guinane, Hans M. Senn, Andrew J. Smith, Martin Wilding, Gregor Mali, Motohiro Nakano, Yuji Miyazaki, Paul McMillan, Gabriele C. Sosso,* Klaas Wynne*

Supplementary Notes

Supplementary Note 1
OKE Data Analysis

OKE spectra consist of a number of broad overlapping bands that are either overdamped or underdamped, which are analysed through curve fitting to the imaginary part of a number of analytical complex functions. The overdamped or diffusive mode is fit to the imaginary part of the Havriliak-Negami function, which is defined in the frequency domain by

$$H_{\alpha,\beta}(\omega) = \frac{1}{\left(1+(-i\omega\tau)^{\alpha}\right)^{\beta}}, \qquad (1)$$

with $0 < \alpha,\beta \leq 1$ and where ω is the angular frequency and τ is the relaxation time. For $\beta = 1$, it reduces to the Cole-Cole function; for $\alpha = 1$ it reduces to the Cole-Davidson function; and for $\alpha = \beta = 1$ it reduces to the Debye function.

However, it would be unphysical if diffusive processes were to be faster than the corresponding underdamped motion. So, diffusive translational relaxation ought to be slower than intermolecular cage-rattling modes. This inertial effect is accommodated using a rise function in the time-domain as described previously.[1] Thus, translational relaxation is fit with an “inertial” Havriliak-Negami function defined by

$$S_{iCD}(\omega) = \mathrm{Im}\left[H_{1,\beta}(\omega) - H_{1,\beta}(\omega + i\gamma_{rise})\right], \qquad (2)$$

where γ_{rise} is the approximate frequency of the intermolecular mode.

In the terahertz range, one finds bands from modes that are not diffusive but critically damped or underdamped. These originate in librations, vibrations, and phonon-like modes. These were fitted using the Brownian oscillator function[2,3]

$$S_{BO}(\omega) = \mathrm{Im}\frac{\omega_0^2}{\omega_0^2 - \omega^2 - i\gamma\omega}, \qquad (3)$$

where ω_0 is the undamped oscillator angular frequency and γ is the damping rate. For the intermolecular modes, it is required to take into account inhomogeneous broadening and the bands are fitted using the antisymmetrised Gaussian function defined by

$$S_G(\omega) = \frac{1}{\sigma\sqrt{2\pi}}\left[e^{-\frac{(\omega-\omega_0)^2}{2\sigma^2}} - \mathrm{e}^{-\frac{(\omega+\omega_0)^2}{2\sigma^2}}\right] \qquad (4)$$

where σ is a width parameter.

The parameters of these functions, listed in Supplementary Table 1, were adjusted using the following procedure. Initially, it was determined that the minimum number of functions needed to describe the spectra in the range between 1 GHz and 10 THz was one inertial Havriliak-Negami, two antisymmetrised Gaussian, and 5 Brownian functions. Each experimental OKE spectrum was then fitted independently following a random sequence of temperatures to avoid systematic errors. For each fit, the parameters of a previous randomly selected fit were used as initial values.

When leaving all the fit parameters free to change, the amplitude of the Brownian oscillator (peaking at *circa* 3 THz) stays constant within the accuracy of the data, consistent with it being an intramolecular mode. In subsequent fitting, its amplitude was set to be constant. Similarly, the parameter τ_{rise} of the inertial Havriliak-Negami function remained constant at temperatures above 200 K, so it too was fixed during the fits.

The rest of the parameters were not fixed. The validity of the fits was subsequently checked by evaluating their change with temperature, which could be linear as in Figure 1(d), exponential as in Figure 2 or, in the case of the translational-relaxation time, following the Stokes-Einstein expression (Supplementary Figure 7).

Supplementary Note 2
Analysis of WAXS Data

The WAXS data can be fitted with two analytical functions, a Lorentzian

$$L_i(q) = L_i \frac{\gamma_i/\pi}{\gamma_i^2 + (q-q_i)^2}, \qquad (5)$$

and a Gaussian

$$G_i(q) = \frac{G_i}{\sqrt{2\pi}\sigma}\exp\left(-\frac{(q-q_i)^2}{2\sigma_i^2}\right), \qquad (6)$$

both of which are normalised by area. In these equations, q_i is the peak position, γ_i the Lorentzian width parameter, σ_i the Gaussian width parameter, and L_i and G_i amplitudes. A total of 4 Gaussians and 1 Lorentzian

were required to fit the WAXS data giving the fit function

$$S(q) = G_3(q) + G_4(q) + L_5(q) + G_6(q) + G_7(q). \quad (7)$$

The pair-distribution function is calculated from the fit functions using[4]

$$g(r) = 1 + \frac{1}{2\pi^2 r \rho_0} \int_0^\infty q\left[S(q) - 1\right]\sin(qr)\,dq. \quad (8)$$

The SAXS data additionally required a Porod function for the low q region

$$P_i(q) = P_i \frac{10^{-2n}}{q^2} \quad (9)$$

as well an additional Gaussian for the boson peak, giving the fit function

$$S(q) = P_1(q) + G_2(q) + G_3(q) + G_4(q) + L_5(q) + G_6(q) + G_7(q) + G_8(q). \quad (10)$$

Supplementary Note 3 Details of MD Simulations and Computational Analysis

Via atomistic MD simulations (see the Methods section for the computational details), a 512-molecule model of liquid TBOS was generated, which was quenched (at a rate of 7.5×10^8 K/s) into the glass. The resulting T_g, obtained from the thermal expansion data reported in Supplementary Figure 12 is 249±20 K. It should be noted that this value is significantly higher than its experimental counterpart (124 K). However, it is expected for the simulations to overestimate T_g due to the much faster cooling rate. The MD data reported hereafter refer to 10 ns long simulations performed at a given temperature within the NPT ensemble.

We have used Packmol[5] to generate the initial random arrangement of TBOS molecules within the simulation box. Details about the algorithm used by this particular piece of software can be found in Ref. [6]. However, we note that, given we have thoroughly equilibrated the system in a highly diffusive regime, the initial (random) configuration of the TBOS molecules within the simulation box does not have any impact on our subsequent molecular dynamics simulations.

The Fourier transform of the Si-Si velocity-velocity autocorrelation function (see the Methods section for the computational details) should display similar trends to the OKE signal measured experimentally, due to the symmetry considerations discussed in the previous section. Indeed, the results reported in Figure 4(a) are in good agreement with the experimental data, particularly in terms of the decrease of the intensity of signal upon cooling as well as the shift of the signal to higher frequencies upon cooling. We note that, as our results are derived from finite temperature MD simulations, the "simulated OKE spectrum" does implicitly take into account anharmonic effects.

Moving on to the structural features of TBOS, the MD simulations identified the emergence of a low-q feature within the total (static) structure factor, reported in Figure 4(b) – thus providing further evidence for the pre-peak observed experimentally, see Figure 3(a). This pre-peak was observed in a 216-molecule model of TBOS as well (generated following the exact same computational protocol), however to a lesser extent—likely because of the fact that this feature corresponds in subtle structural changes spanning up to ~25 Å in real space—hence the need for a large-enough TBOS model. A comparison between the total (static) structure factor obtained for the 216- and 512-molecule models is reported in Supplementary Figure 12. The partial (static) structure factors, obtained for every other atomic species in the system (as opposed to Si only, and we the exception of hydrogen atoms) are reported in Supplementary Figure 19. We note that in calculating the $S(q)$ we have considered both intra- and inter-molecular distances. The emergence of the low-q pre-peak can be observed for, *e.g.*, the O-O $S(q)$ as well, and it is particularly clear if we focus on the O-O inter-molecular distances only, as illustrated in Supplementary Figure 20.

In order to make sure that the emergence of the pre-peak in our simulated $S(q)$ represents a genuine structural feature of our TBOS models, we have generated three additional, statistically independent models, following exactly the same computational protocol. The total and Si-Si $S(q)$ is reported as a function of temperature in Supplementary Figure 13. Note that, despite the (expected) variability of the $S(q)$ as a whole (on our scale we are bound to generate/observe an ensemble of slightly different amorphous systems) the pre-peak is present in all the three models. We also report in Supplementary Figure 13 the Si-Si PCF in the range corresponding to the low-q region of the $S(q)$ where we observe the pre-peak – not the emergence of long range-order. Finally, we have thoroughly investigated the influence of sampling (*i.e.*, how many frames we considered within a certain time interval) and binning (*i.e.*, the "resolution" of the PCF), and found our results to be 100% robust in that respect.

The short-range structural changes are more evident in the Si-Si pair correlation function reported in Figure 4(c). It is interesting to observe the significant increase in terms of short-range order, particularly as it concerns the second coordination shell. In fact, the running coordination number reported in Figure 4(d) provides further insight into the topology of the TBOS network. The first coordination shell (which extends up to ~8 Å) only contains, on average, about 2 molecules – which shows the lack of tetrahedral order in both liquid and glassy TBOS. However, the second coordination shell (which extends up to ~12 Å) contains about 12 molecules – a number that appears to monotonically, if slightly, increase upon cooling. The pair correlation functions obtained for every other atomic species in the system (as opposed to

Si only, and we the exception of hydrogen atoms) are reported in Supplementary Figure 21.

To gain further insight into these structural changes and attempt to link them to the dynamical properties highlighted by the OKE measurements, a Voronoi analysis was performed (see the Methods section for further computational details), which, amongst other aspects, provides an "effective" coordination number whose definition does not depend on the choice of any specific cutoff radius. In the context of e.g. metallic alloys, the Voronoi Polyhedron (VP) corresponding to any given atom is usually labelled[7] in terms of Schläfli notation via a tuple of four indices as <n_3, n_4, n_5, n_6>, where n_i refers to the number of faces with i vertexes (or, equivalently, edges) within that particular VP. However, it was found that the complexity of the TBOS network is such that a more extensive indexing is required, namely <n_3, n_4, n_5, n_6, n_7, n_8, n_9, n_{10}>, to fully appreciate the topology of the system. This indexing has been used before when dealing with complex molecular systems displaying a huge variety of VP.[8] However, the overwhelming majority of VPs observed is characterised by $n_9 = n_{10} = 0$.

We start by investigating the distribution of the total number of faces characterising each VP. To this end, 1,000 configurations were considered of the TBOS model at any given temperature, across a 20 ns long MD simulation. The total number of faces corresponds to the effective coordination number of any given j-th Si atom (the only species considered for the Voronoi analysis), as $CN_j = \sum_{i=1}^{10} n_i$. To reduce the complexity of the Voronoi analysis, faces that account for less than 1% of the total surface area of a given VP were ignored—in line with the approach of, *e.g.*, Ref. [9] —aimed at avoiding overcounting and remove too small structural fluctuations.

The results are reported, as a function of temperature, in Figure 5(a). Overall, it is clear that the network of Si atoms within TBOS is predominantly 12-coordinate at any given temperature. Whilst the sheer variety of VP indices (more than 1,000) prevents a quantitative statement about the exact topology of the system, we observe traces of both icosahedral and FCC order (*e.g.*, VP indices <0,4,4,5,0,0,0,0> and <0,3,6,4,0,0,0,0>) in the supercooled liquid. These VP, however, disappear below T_g. However, the frequency by which we observe the <0,4,4,2,0,0,0,0> VP, which is characteristic of HCP order, slightly increase just below T_g before decreasing again at very low T (*i.e.*, ~ 90 K). These trends are supported by the analysis of the local environments (described in Ref. [10] and implemented in Ovito) summarised in Supplementary Figure 14(a), which shows a tendency toward HCP order for the supercooled liquid. This tendency, similarly, to what we observed for the <0,4,4,2,0,0,0,0> VP, decreases as we approach T_g.

As an additional indication of the absence of long-range order within both supercooled liquid and glassy TBOS, we have conducted a bond-orientational order analysis, utilising the Steinhardt parameters q_4 and q_6 according to the revised, "local" implementation described in Ref. [11]. The results as summarised in Supplementary Figure 14 (b). The very low values of q_6 indicated the absence of crystalline order, whilst the values we observe for q_4 sits around those expected for HCP order – which, consistently with both the VP and the local environments analysis, diminishes with temperature.

It can also be seen that the number of faces of the VP increases on cooling, consistent with the structuring of the Si-Si PCF illustrated in Figure 4(a). In particular, a proliferation of VP is observed, characterised by 15 and even 16 faces below T_g. For instance, the <0,4,6,2,2,1,0,0> and <0,4,6,4,2,0,0,0> VP are unique to the glassy state. Even more telling is the distribution of the volumes of the VPs as a function of temperature, reported in Figure 5(b). In this case, a split in the probability density function of said VP volumes can clearly be identified below T_g, which must correspond to the emergence of specific structural features.

Indeed, if the most frequent VP at each temperature are clustered together based on a simple distance criterion (see the Methods section for further computational details), a decisive increase in the size of the largest clusters formed by TBOS molecules characterised by VP with more than 15 faces is observed – as shown in Supplementary Figure 15. This is indicative of the formation of the over-coordinated structures that can be inferred from Figure 5(a) and Figure 5(b).

While it is not possible to make a quantitative connection with the supra-molecular structures identified by means of the OKE measurements (even slower cooling rates and even larger models might be needed to offer a quantitative comparison with the experimental data), the MD simulations are strongly suggestive that these structures are clusters of over-coordinated TBOS molecules which – were if not for the presence of the long side chains on top of the tetrahedral Si – would tend toward a BCC-like structural order.

Finally, as below T_g the self-diffusion of the system is so low that the local molecular environments tend to remain unchanged within the 20 ns timescale investigated in our MD simulations, one can attempt to "project", so to speak, the power spectrum of the system onto specific Si atoms characterised by specific VP. It is found that VPs characterised by 16 faces tend to populate the high-frequency region of the power spectra, as illustrated in Figure 5(d) at 90 K. Therefore, the increase in the local coordination of the TBOS molecules might also be part responsible for the shift of the OKE signal toward higher frequencies upon cooling in addition to the collision-induced effects.

In terms of the orientational order of TBOS molecules, the latter be considered as approximately tetrahedral units. In order to assess whether TBOS molecules would assume any preferential orientation within the glass, we have computed, for each TBOS molecule, the angle Θ_z formed by each of the four Si-O molecular axes (defined as the vectors originating from the Si atom and

pointing toward each of the four oxygen atoms) and the Cartesian z-axis. We have then averaged these four angles over all the molecules within a given frame to obtain an average orientation $<\Theta>_z$. The probability density function of $<\Theta>_z$, computed over 1,000 statistically independent frames within 10 ns long trajectories, is reported in Supplementary Figure 16(a), as a function of temperature. It can be seen that the distributions peak at ~90° at every temperature, which demonstrates that the system is isotropic even in the glassy state. Note the narrowing of the distribution as the temperature decreases – which is indicative of the much slower rotational dynamics within the glassy state.

Next, we wanted to investigate the relative orientation of the TBOS tetrahedral units within their local environments. To this end, we define, for each molecule, the plane illustrated in Supplementary Figure 16(b). We then leverage the so-called Solid Molecule Angle Criteria (SMAC) order parameter, described in Ref. [12]. In a nutshell, SMAC measures the degree to which molecules, within a certain distance from each other, are similarly oriented. In this case, we considered up to the 2nd coordination shell of the TBOS molecules, which encompasses ~12 neighbors. The orientation of each molecule is defined by the vector normal to the plane illustrated in Supplementary Figure 16(b). Higher (lower) values of SMAC correspond to more (less) ordered molecular environment in terms of the relative orientation of, in this case, the tetrahedral SMAC units.

The results are summarised in Supplementary Figure 16(c), where we have averaged the value of SMAC for each molecule within a given frame (which yields <SMAC>) and then built a probability density function over different frames. Interestingly, it appears that the glassy state is less ordered, in terms of the relative orientation of the TBOS molecules, than the supercooled liquid. This result is consistent with the progressive loss of structural short-range order we have observed (see Figure 5(c) and Supplementary Figure 16), and provides further evidence of the fact that the over-coordinated (Voronoi polyhedra characterised by > 12 faces, particularly 15 and 16 faces) clusters of TBOS molecules we have observed in the glassy state are not characterised by any degree of crystalline order.

Another structural aspect, observed experimentally, which is also captured by our MD simulations, is the progressive decrease, upon cooling, of the relative population of gauche defects within the alkoxide side chains of the TBOS molecules. To illustrate this aspect, we have computed the probability density function of the value of all the H-C-C-H dihedral angles (ϕ) within the alkoxide side chains, averaged across 10 ns at each temperature. The results are summarised in Supplementary Figure 17. As the temperature decreases, the distribution of ϕ narrows (as the mobility of the side chains is greatly reduced in the glassy state) and we observe a reduction of the relative population of gauche configurations with respect to trans configurations (as demonstrated by the ratio between the height of the PDF peaks at ~120° [trans] and ~60° [gauche]). Trans configurations are clearly more energetically favored at any temperature, potentially due to steric effects.

Supplementary Note 4
Relation Between Viscosity and Relaxation Rate

Translational diffusion is described by the Stokes-Einstein equation, which predicts the translational diffusion coefficient to be given by

$$D_{trans} = \frac{k_B T}{6\pi\eta R} \quad (11)$$

If the temperature-dependent viscosity is described by a Vogel-Fulcher-Tammann equation, one would expect the relaxation time to be proportional to

$$\tau \sim T^{-1} \exp\left(\frac{DT_0}{T - T_0}\right) . \quad (12)$$

Supplementary Note 4
Low-temperature Heat Capacity

The heat capacity of an ideal Debye crystal is given by the expression

$$C_D = 9R\left(\frac{T}{T_D}\right)^3 \int_0^{T_D/T} \frac{x^4 e^x}{\left(e^x - 1\right)^2} dx , \quad (13)$$

where R is the gas constant and T_D the Debye temperature. The heat capacity of a mole of oscillators with a single frequency is given by the Einstein expression

$$C_E = 3R\left(\frac{T_E}{T}\right)^2 \frac{e^{T_E/T}}{\left(e^{T_E/T} - 1\right)^2} , \quad (14)$$

where the Einstein temperature is defined as $T_E = h\nu/k_B$ with ν the frequency of the oscillator.

The heat capacity divided by the temperature cubed as shown in Supplementary Figure 18 is mostly sensitive to the heat capacity associated with low frequency modes. Therefore, in fitting these data, one has to take into account lattice vibrations (described by a Debye function), the boson peak or other low frequency modes, and intramolecular vibrational modes and establish a best fit. Thus, the fit function used is

$$C / T^3 = \left(C_D\left(T_D\right) + aC_E\left(T_{boson}\right) + \sum_{i=1}^{3N-6} C_E\left(T_i\right) \right) / T^3 , \quad (15)$$

where the sum runs over the 165 vibrational normal modes of TBOS. The normal-mode frequencies were obtained as described in Methods and scaled by a factor of 0.93 to match the experimental frequencies. A fit to the experimental heat capacity in the Debye representation is shown in Supplementary Figure 22, yielding a Boson mode frequency of 0.6 THz.

Supplementary Note 5
Density of States and the Boson Peak

The vibrational density of states (VDoS) $g(\omega)$ can be measured in several related experiments such as Raman scattering (including OKE spectroscopy), inelastic neutron scattering, *etc*. Such spectroscopies often do not measure $g(\omega)$ directly but instead a product of the VDoS with the strength of the coupling associated with the particular spectroscopy. Thus, the various spectroscopies measure the same dynamics or spectra but with different amplitudes for the various components. For example, the spontaneous Raman scattering (SRS) spectrum is related to the VDoS by[13]

$$S^{SRS}(\omega) \propto g(\omega) C(\omega) \frac{n(\omega,T)+1}{\omega}, \qquad (16)$$

where

$$n(\omega,T) = \frac{1}{e^{\hbar\omega/kT}-1} \qquad (17)$$

is the Bose-Einstein occupation number, and $C(\omega)$ is the Raman coupling coefficient.

It has been suggested that the frequency dependence of the coupling in the SRS spectrum may follow ω^2 at very low frequencies (< 20 cm^{-1}). However, comparison between Raman and inelastic neutron scattering in inorganic glasses (GeO_2, SiO_2, B_2O_3) has shown the Raman coupling coefficient to be linear in frequency in the low terahertz range,[14–19] that is,

$$C(\omega) = \omega \qquad (18)$$

and therefore, one has

$$g(\omega) \cong \frac{S^{SRS}(\omega)}{n(\omega,T)+1}. \qquad (19)$$

The OKE spectrum is given by[2]

$$\begin{aligned} S^{OKE}(\omega) &= \chi''(\omega) \\ &= S^{SRS}(\omega)\frac{1-e^{-\hbar\omega/kT}}{2\hbar^2} \\ &\propto g(\omega)\frac{C(\omega)}{\omega} \end{aligned} \qquad (20)$$

and is also referred to as the susceptibility representation of the spontaneous depolarised Raman spectrum. Making the assumption of Eq. (18), one has

$$S^{OKE}(\omega) \cong g(\omega). \qquad (21)$$

Supplementary Figures

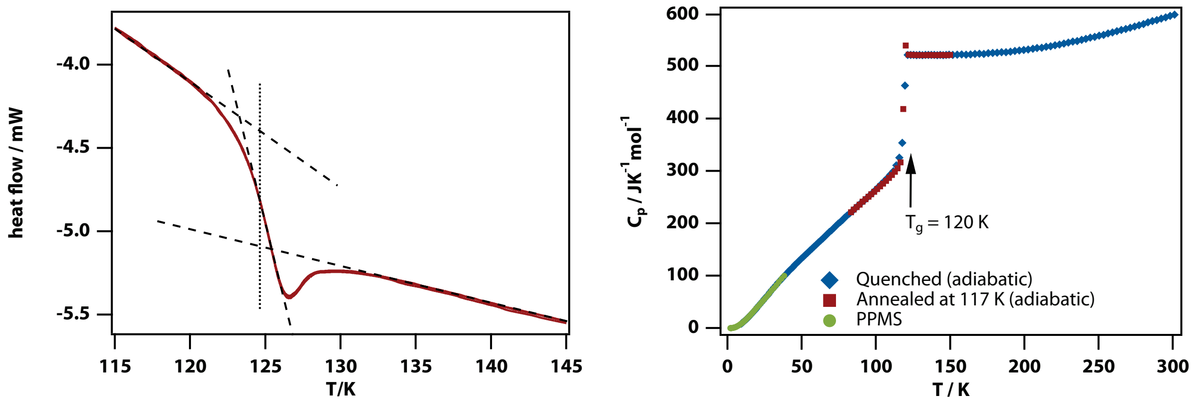

Supplementary Figure 1. Heat capacity measurements of TBOS. (**left**) Measured using the TA Instruments DSC 2500 (three scans superimposed) using quench cooling followed by controlled heating showing a glass transition at T_g = 124 K. (**right**) Heat capacity measured using a PPMS relaxation calorimeter (<20 K) and an adiabatic calorimeter (>10 K) showing a glass transition at T_g = 120 K.

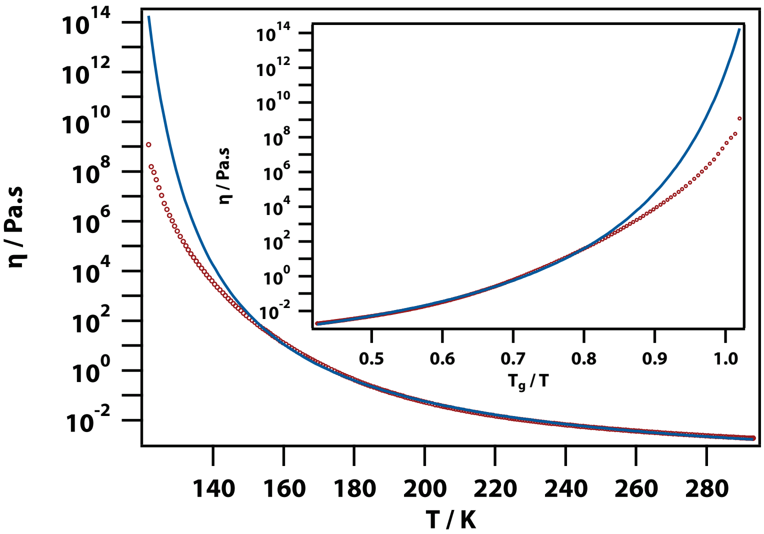

Supplementary Figure 2. Temperature-dependent viscosity of TBOS and Angell plot using the measured glass transition temperature of T_g = 124 K. Also shown is a fit (using data from 150 to 293 K) to the Vogel-Fulcher-Tammann expression for viscosity, $\eta = \eta_0 \exp(DT_0/(T-T_0))$, with η_0 = 4.62 10^{-5} ± 0.21 10^{-5} Pa s, D = 6.4±0.1 K, and T_0 = 105.4±0.5 K.

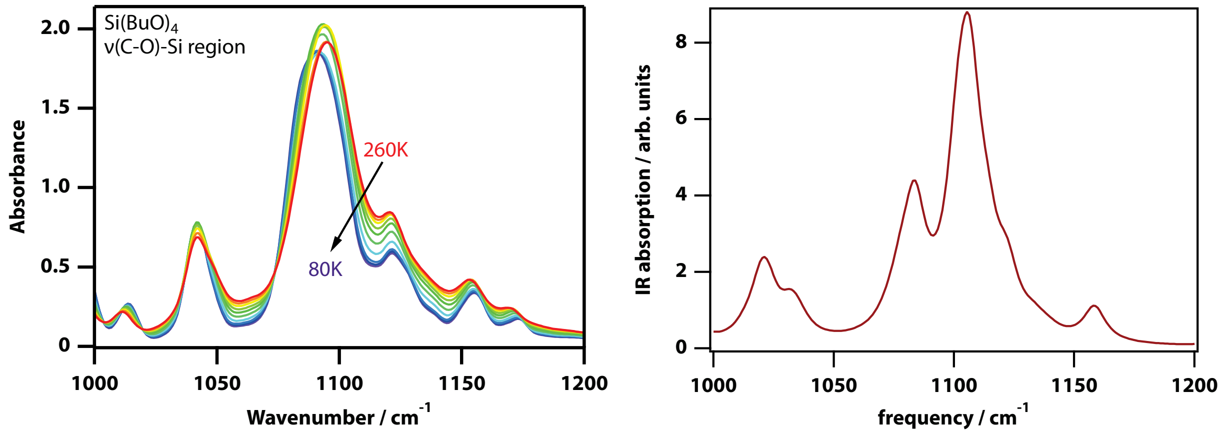

Supplementary Figure 3. Infrared spectra of TBOS in the mid-infrared region. (left) Experimental temperature-dependent IR spectra of liquid and vitrified TBOS. (right) Computed IR spectrum of TBOS. Here the frequencies have been multiplied by 0.936 to match up the experimental and computed spectra in the CH-stretch region (~2900 cm^{-1}).

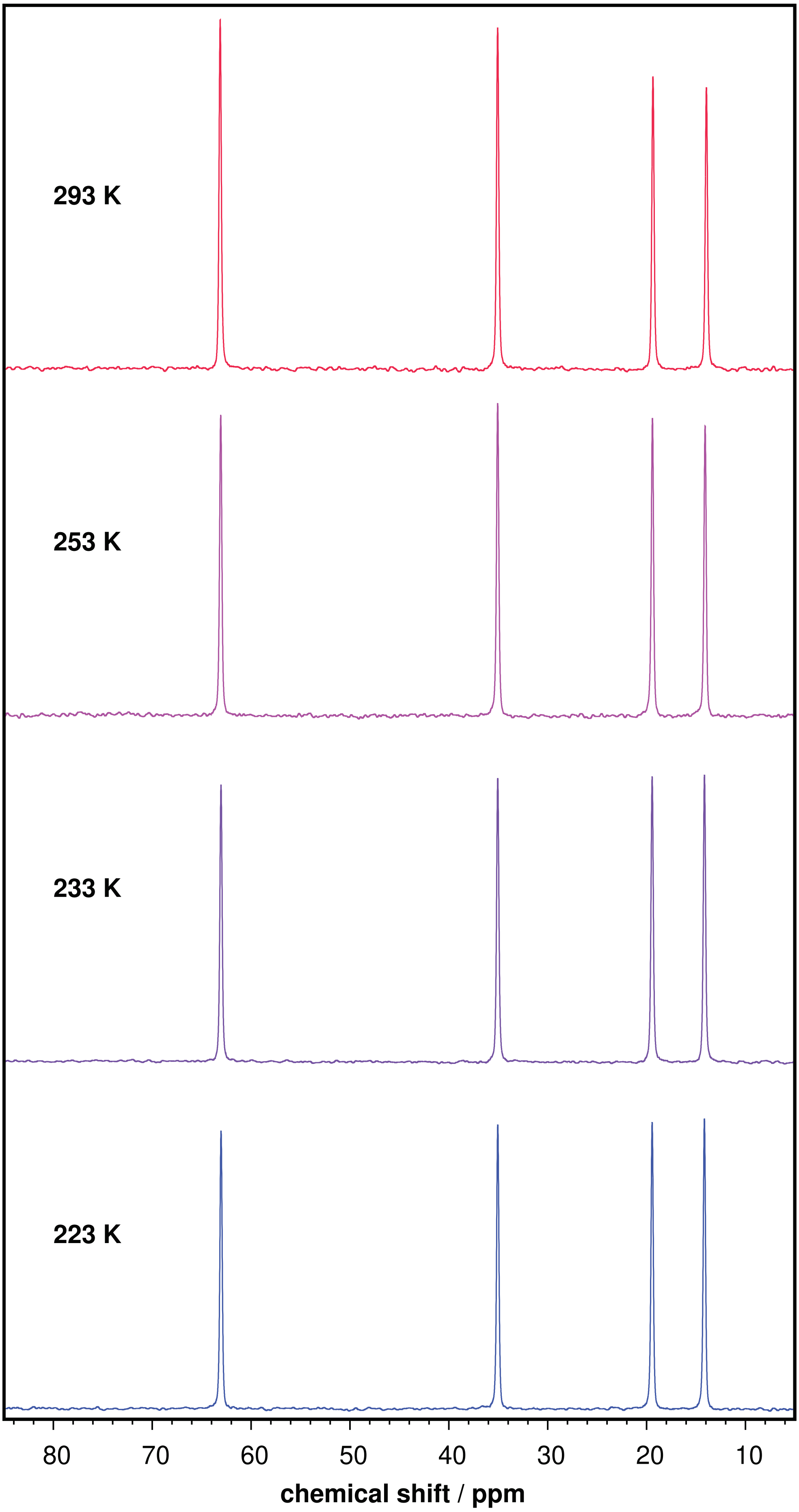

Supplementary Figure 4. Temperature-dependent ^{13}C NMR spectra of TBOS.

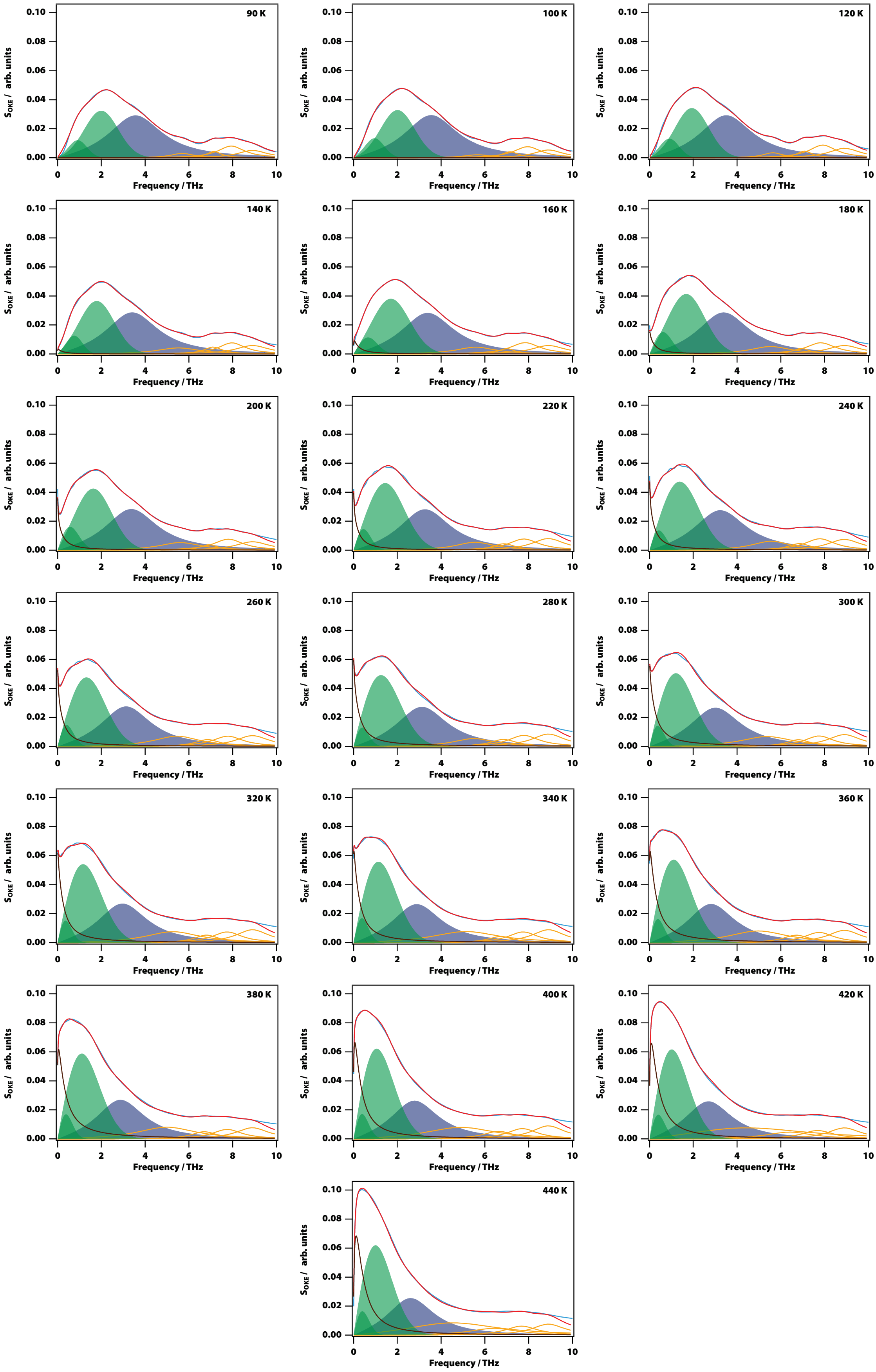

Supplementary Figure 5. All OKE data on a linear frequency scale.

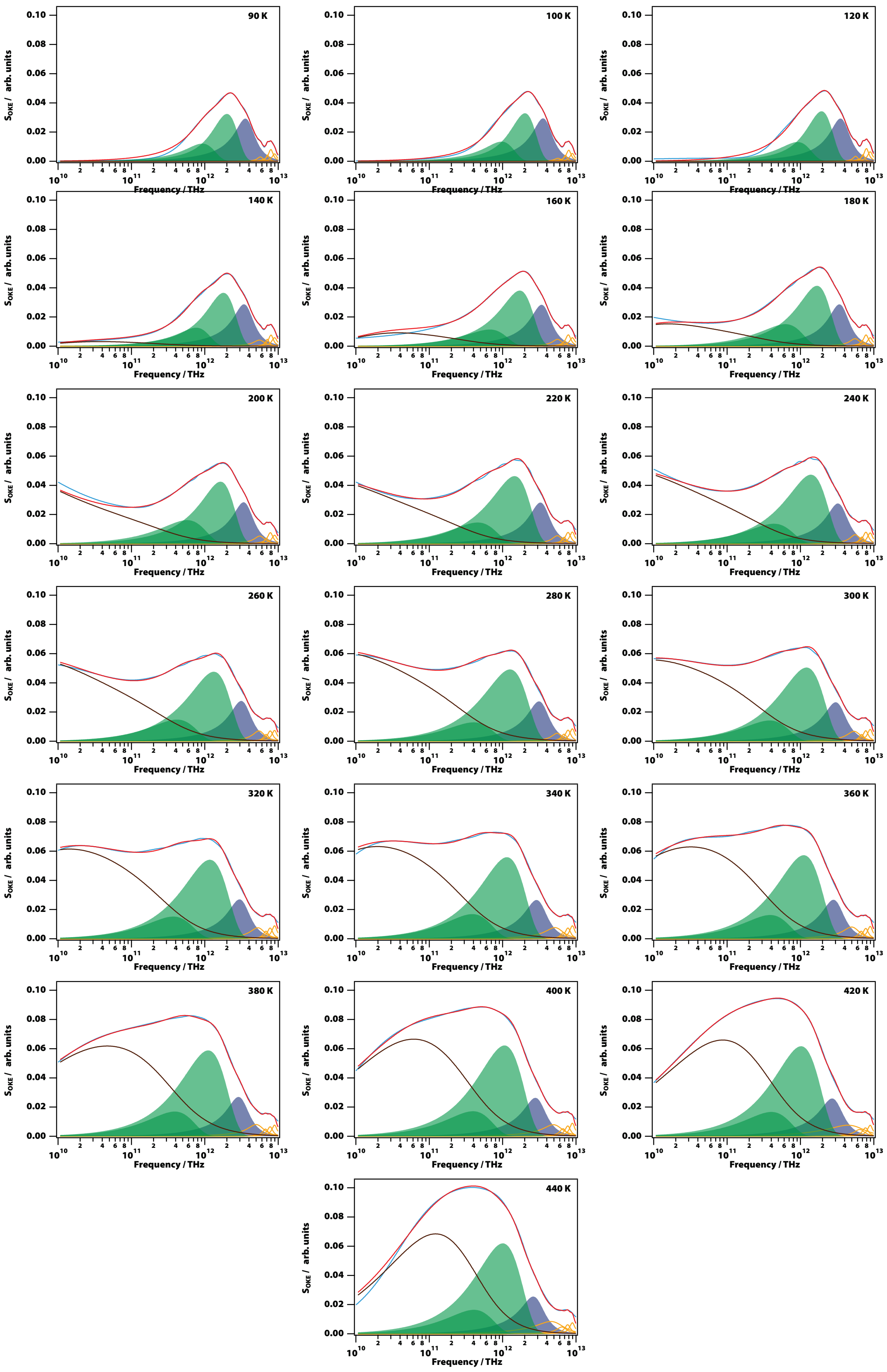

Supplementary Figure 6. All OKE data on a logarithmic frequency scale.

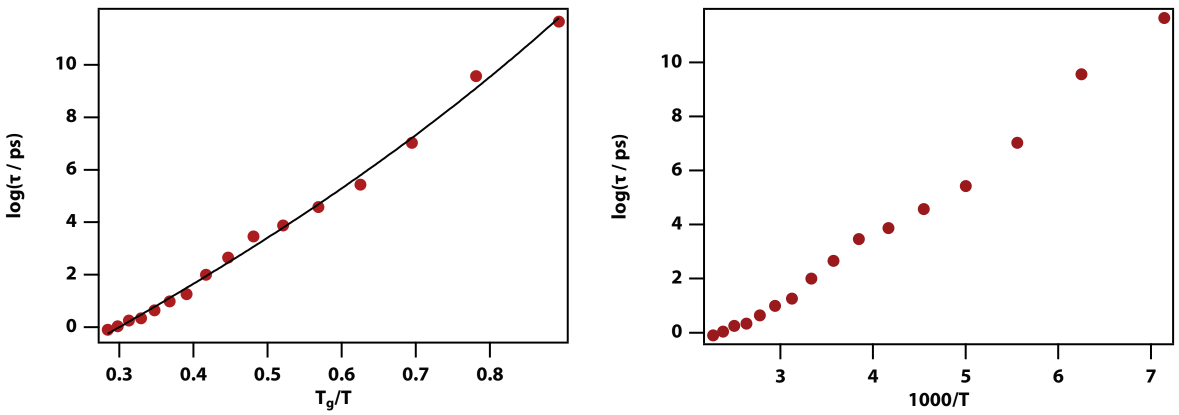

Supplementary Figure 7. Angell plot of the temperature-dependent translational-relaxation time constant of TBOS using the measured glass transition temperature of T_g = 125 K. Also shown is a fit to the Stokes-Einstein expression for the translational diffusion relaxation time using a Vogel-Fulcher-Tammann expression for viscosity, $\tau = (\eta_0/T)\ \exp(DT_0/(T\text{-}T_0))$, with η_0 = 0.05±0.04 cP, D = 78±22 K, and T_0 = 43±8 K. (**right**) Same on a 1000/T scale.

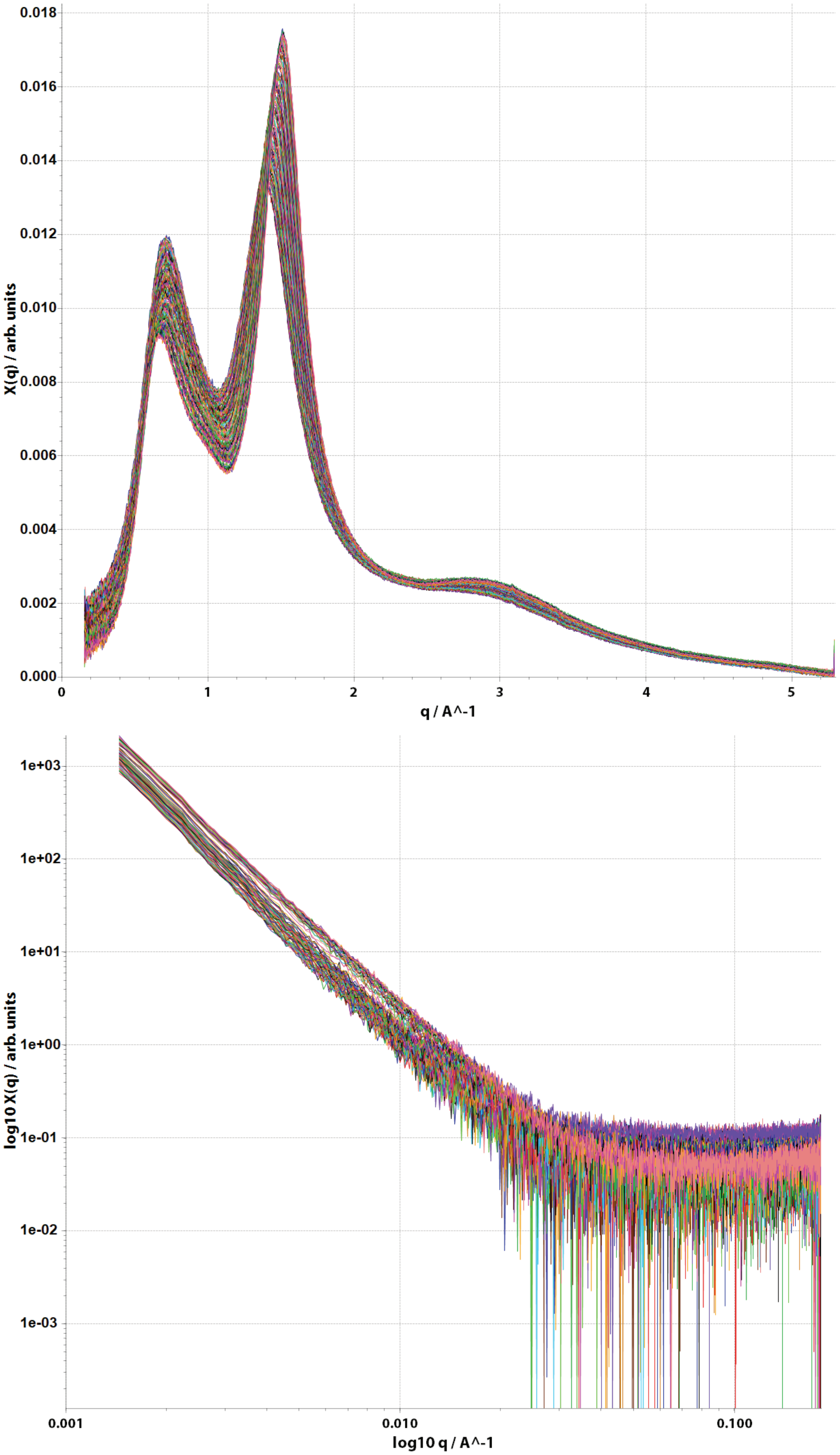

Supplementary Figure 8. Background subtracted WAXS (top) and SAXS (bottom) data on TBOS from 25° to -181°C. The SAXS data are shown on a logarithmic scale and follow a Porod law with an exponent of 3.5.

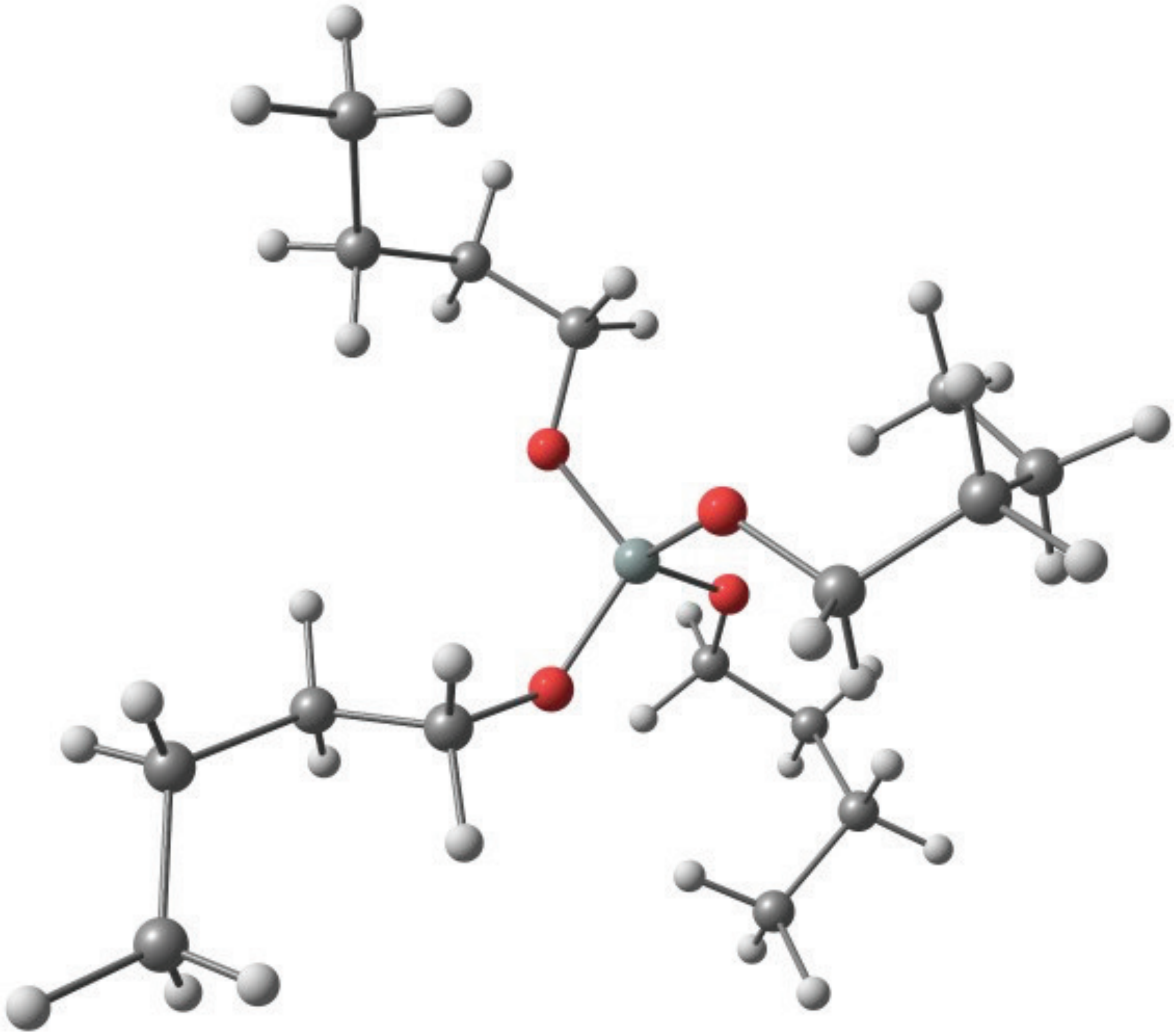

Supplementary Figure 9. Model of TBOS. Structure obtained by wB97X-D3/ma-def2-SVP. Based on the molecular weight of TBOS of 320.54 g/mol and its density at 25°C of 0.899 kg/l (Sigma-Aldrich), one calculates a molecular volume of 592 Å^3 or (assuming a spherical shape) a hydrodynamic radius of 5.2 Å.

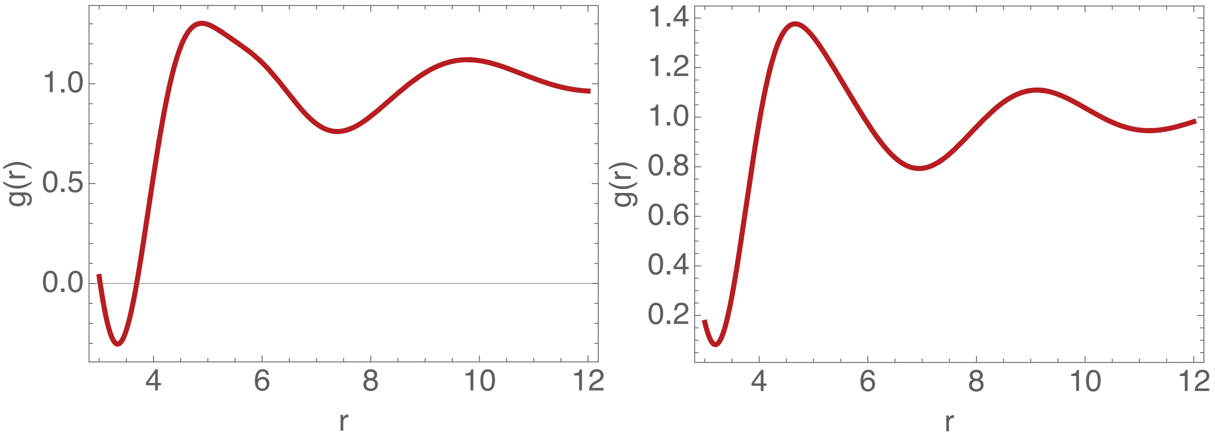

Supplementary Figure 10. Radial distribution functions calculated from the fits to the WAXS data and analytical transformation (see Supplementary Note 2). Comparison of the result at 25°C (left) and -110°C (right) shows a contraction of both the first and second solvation shell.

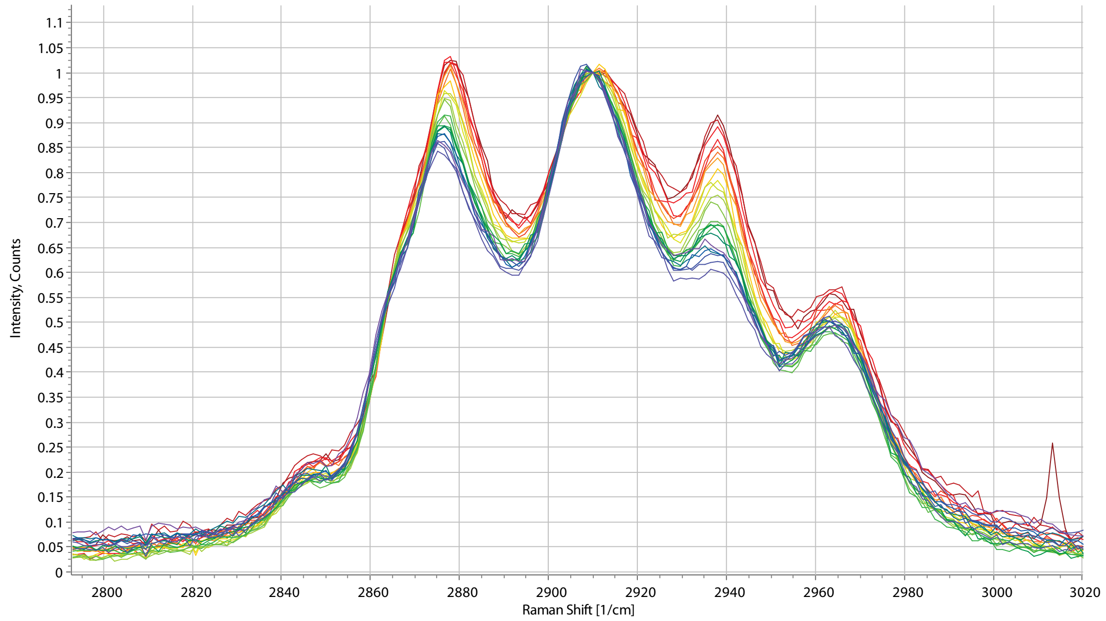

Supplementary Figure 11. Temperature-dependent (40°C to -180°C) Raman spectra in the CH-stretch region of TBOS normalised at 2910 cm^{-1}. The peak height ratio I_{2885}/I_{2850} is sensitive to chain packing type,[20,21] showing that the butoxide side chains are becoming all-trans at low temperature.

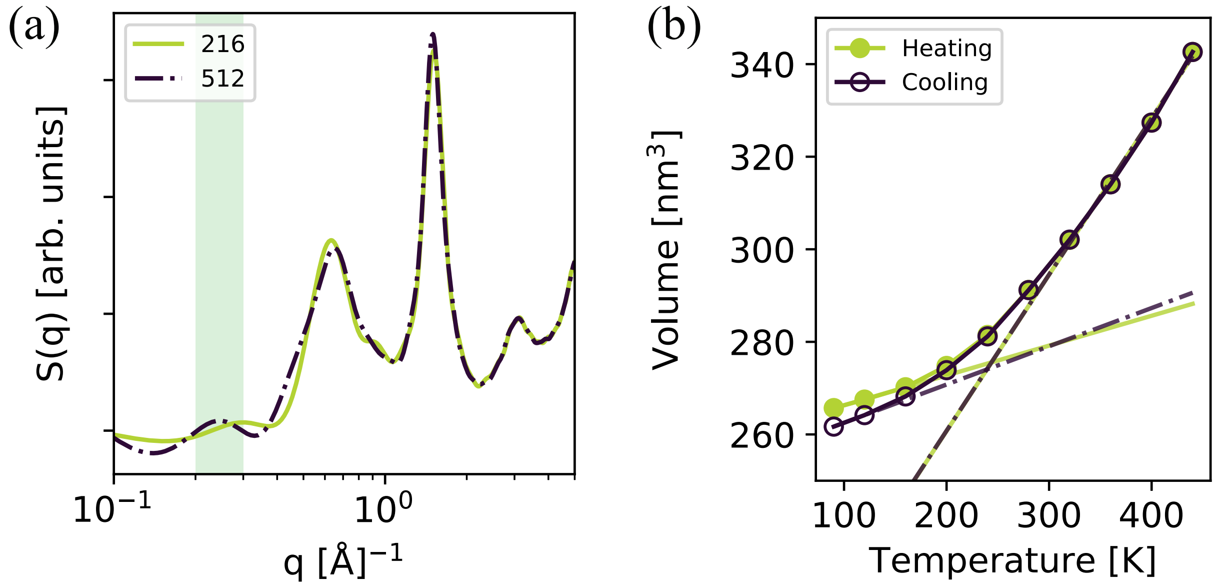

Supplementary Figure 12. Finite size effects and estimation of the glass transition temperature in the MD simulations. (a) Total (static) structure factor computed at 90K for a 216- and a 512-molecule model of TBOS. The region shaded in green highlights the emergence of the pre-peak in the structure factor discussed in the main text. (b) Average value of the simulation box for the 512-molecule model of TBOS as a function of temperature. T_g has been obtained from the intersection of the two linear fits with respect to the data points relative to the glass ($T < 200$ K) and the supercooled liquid ($T > 300$ K) respectively. The results are reported for both a heating and a cooling ramp, which give $T_g = 249 \pm 20$ and 241 ± 20 K, respectively.

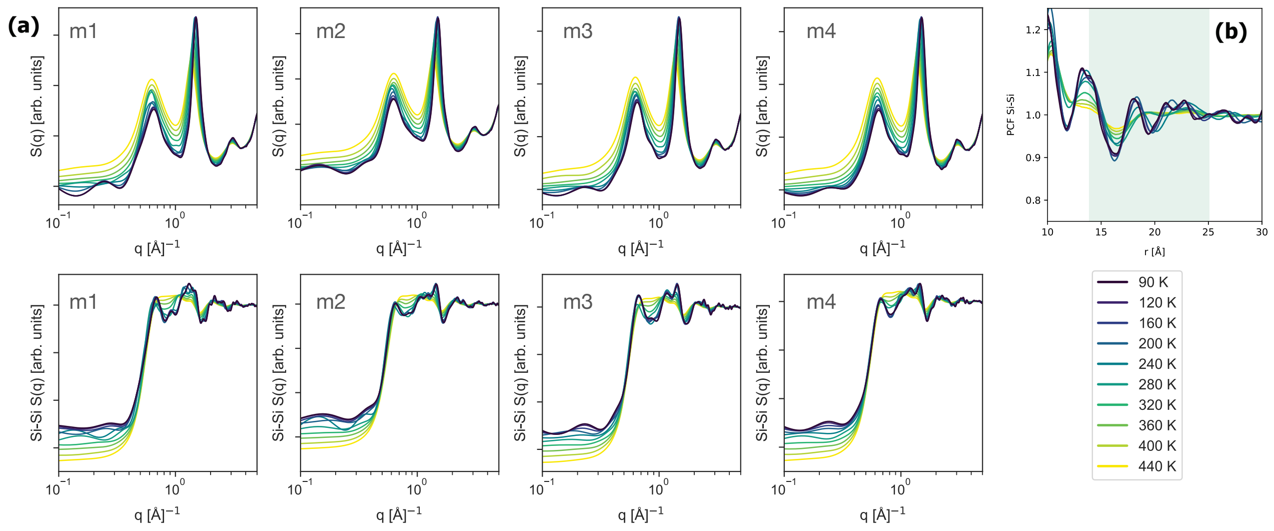

Supplementary Figure 13. (a) Total and Si-Si structure factor $S(q)$ as a function of temperature for four statistically independent models of TBOS. (b) Si-Si pair correlation function (PCF) as a function of temperature. The shaded region corresponds to the 0.25-0.45 Å^{-1} region in reciprocal space.

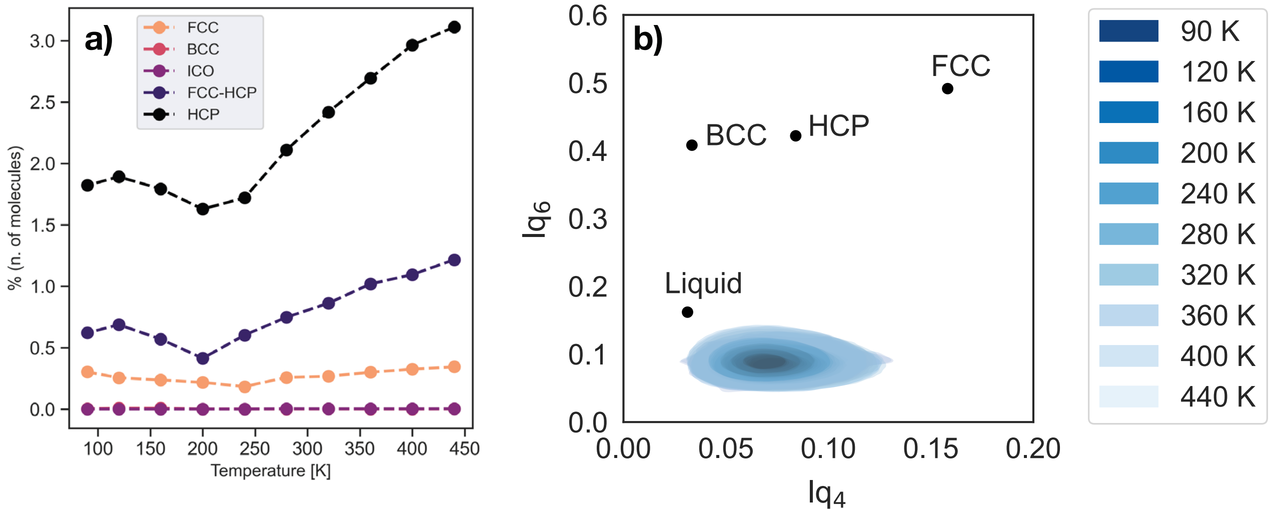

Supplementary Figure 14. (a) Percentage of TBOS molecules characterised by specific crystalline order, as a function of temperature, obtained according to the framework described in Ref. [10] (b) Steinhardt order parameters q_4 and q_6 (computed according to the local average put forward in Ref. [11] for each Si atom in the system, as a function of temperature. All the results reported in this figure have been averaged over 1000 statistically independent frames within 10 ns long MD trajectories.

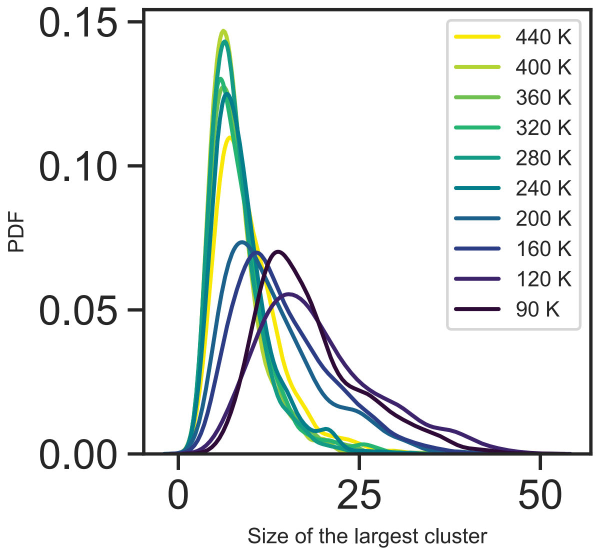

Supplementary Figure 15. Probability density function (PDF) of the size of the largest cluster formed by TBOS molecules characterised by Voronoi polyhedra with 15 or more faces, as a function of temperature. We identified the largest cluster for 1000 statistically independent frames within 10 ns long MD trajectories.

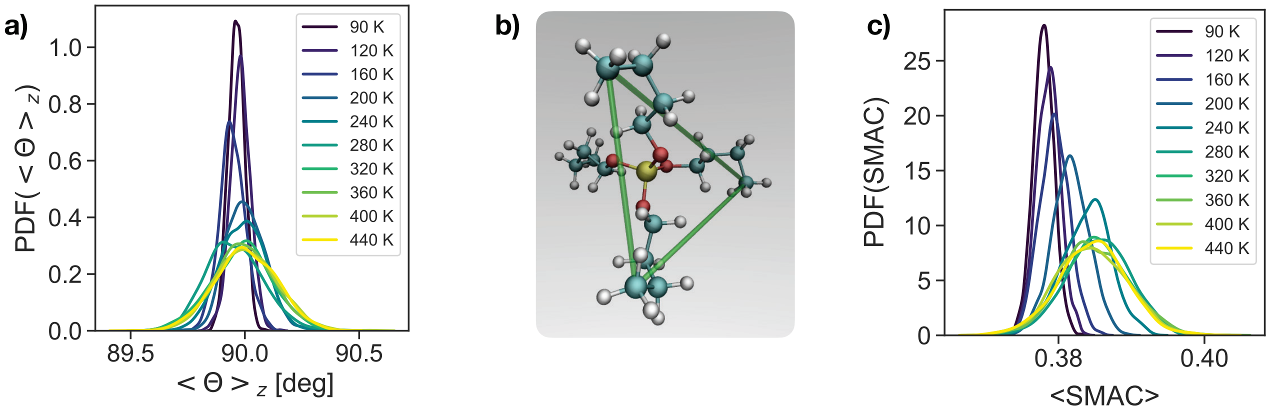

Supplementary Figure 16. Probability density function (PDF) of the average value of the angle Θ_z (see text) as a function of temperature. (a) The value of Θ_z for an entirely random average orientation of the molecular axis is 90°. (b) Definition of the molecular plane (green lines) identifying the orientation of a TBOS molecule / tetrahedral unit. (c) PDF of the average value of the SMAC order parameter (see text) as a function of temperature. All the results reported in this figure have been averaged over 1000, statistically independent frames within 10-ns long MD trajectories.

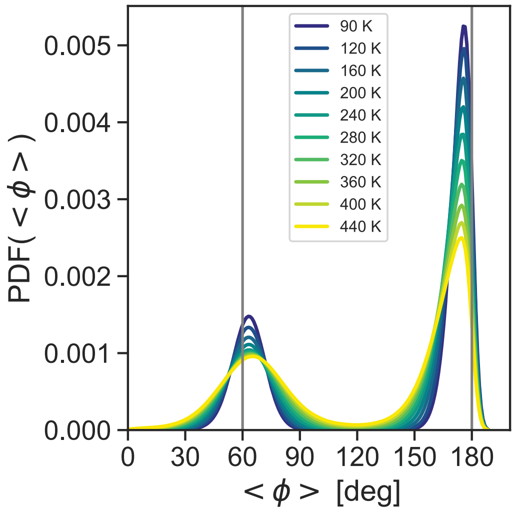

Supplementary Figure 17. Probability density function (PDF) of the averaged values of the H-C-C-H dihedral angles, ϕ, within the alkoxide side chains of TBOS molecules, as a function of temperature. The two grey lines at $\phi = 60°$ and 120° indicate typical values of gauche and trans configurations, respectively.

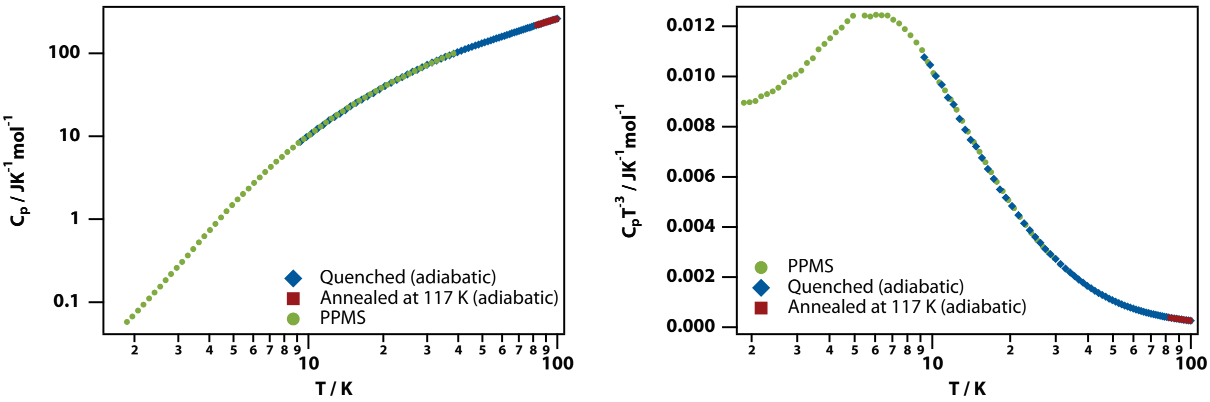

Supplementary Figure 18. Low temperature heat capacity TBOS measured using a PPMS relaxation calorimeter (<20 K) and an adiabatic calorimeter (>10 K). (left) Heat capacity and (right) heat capacity divided by temperature cubed.

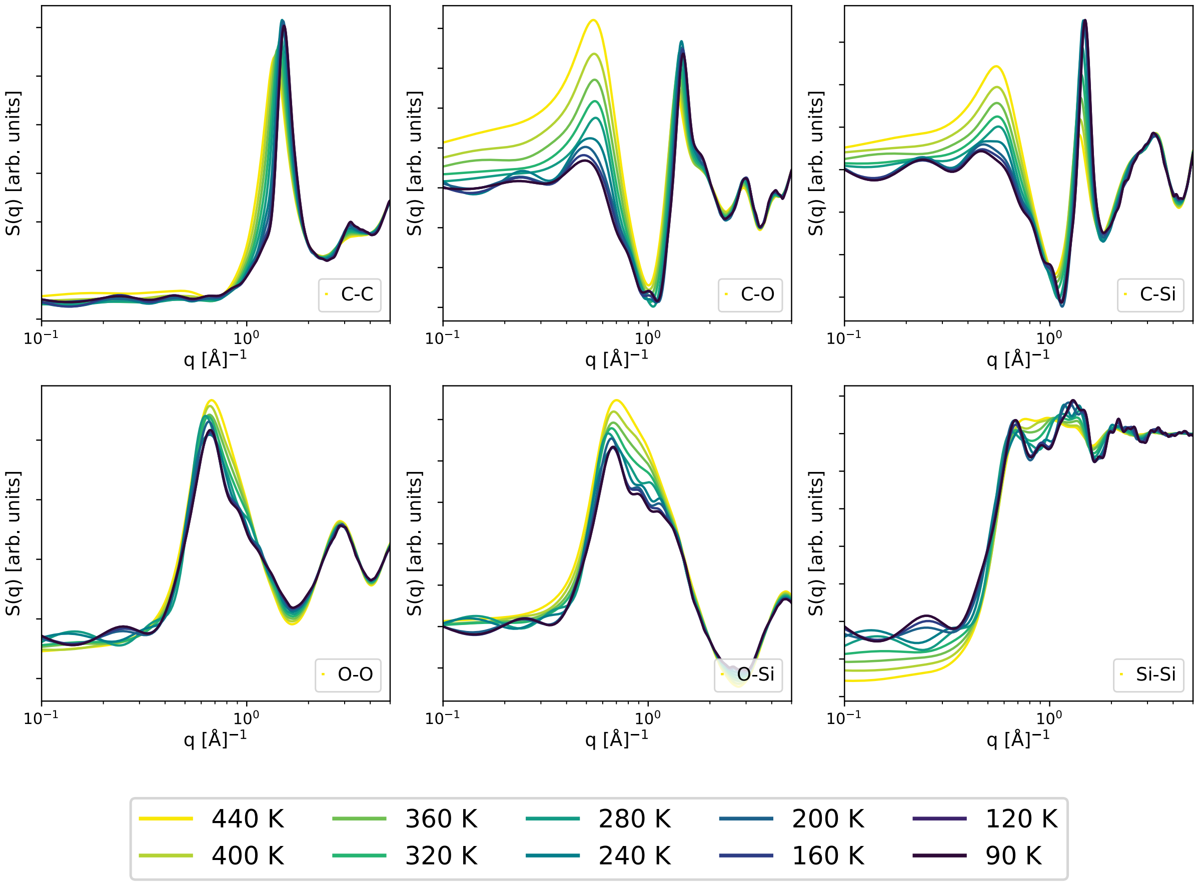

Supplementary Figure 19. (Static) structure factor for a 512-molecule model of TBOS as a function of temperature. The results are reported for every individual pair of atomic species (exception made for hydrogen atoms).

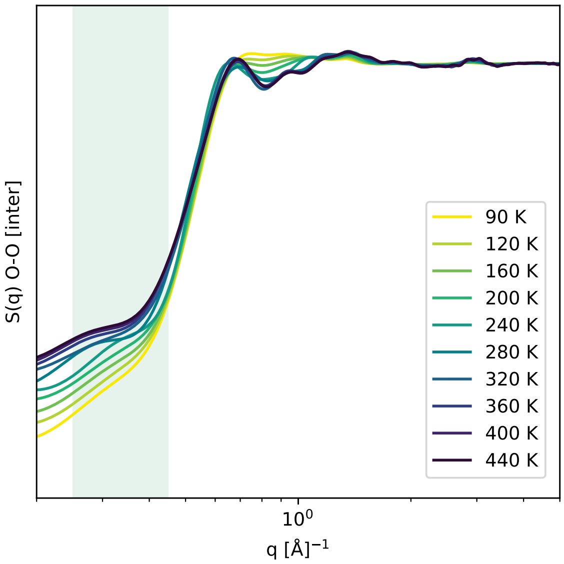

Supplementary Figure 20. (Static) structure factor $S(q)$, relative to the intermolecular O-O distances only, for a 512-molecule model of TBOS as a function of temperature. The shaded region highlights the emergence of the pre-peak discussed in the main text.

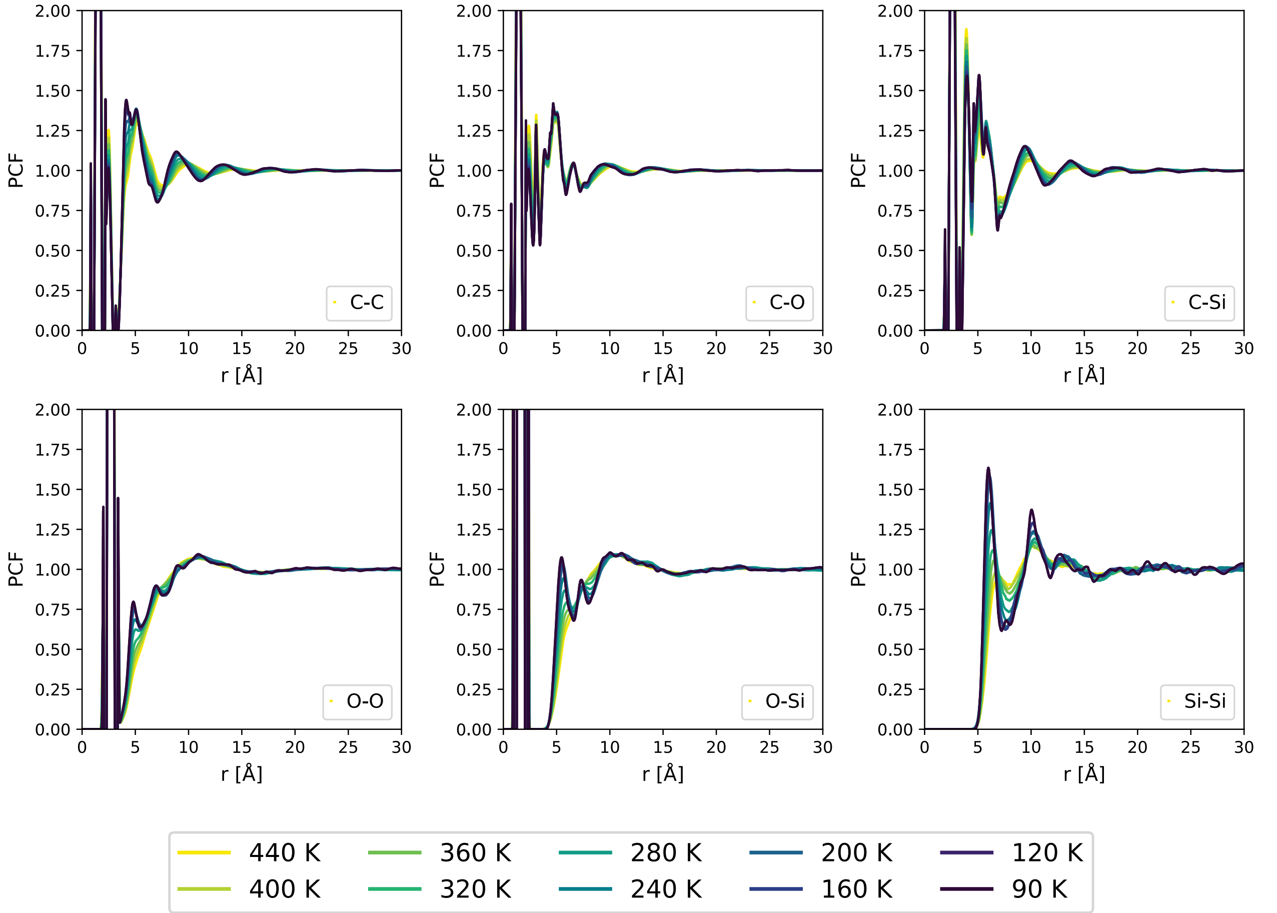

Supplementary Figure 21. Pair correlation functions (PCF) for a 512-molecule model of TBOS as a function of temperature. The results are reported for every individual pair of atomic species (exception made for hydrogen atoms).

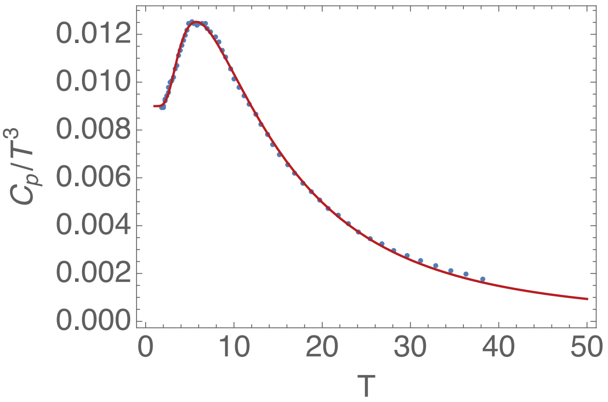

Supplementary Figure 22. Fit to the experimental heat capacity in the Debye representation with $T_D = 60$ K and $T_{boson} = 27$ K (ν_{boson} = 0.6 THz).

Supplementary Tables

Temp	HN				G_1			G_2			B_1		
K	A	t /ps	β	$10^{\tau rise}$ / ps	A	ω_0 / GHz	σ /GHz	A	ω_0 / GHz	σ /GHz	A	ω_0 / GHz	γ /GHz
**90**					12727	921	420	63176	1996	776	139.6	3860	3050
**100**					14858	962	439	63419	2012	781	139.6	3841	3016
**120**					14619	908	442	66246	1938	771	139.6	3800	2996
**140**	19196	$4.48\ 10^{11}$	0.304	-2.00	12680	753	401	74121	1790	808	139.6	3709	2989
**160**	12751	$3.73\ 10^{9}$	0.304	-2.00	13978	642	429	81792	1718	855	139.6	3691	3026
**180**	3102	$1.07\ 10^{7}$	0.304	-1.58	16235	604	422	88345	1676	851	139.6	3687	2976
**200**	1689	$2.70\ 10^{5}$	0.304	9.06	19531	509	450	90485	1629	848	139.6	3676	2998
**220**	1192	38163	0.304	-0.88	14976	390	373	103709	1435	888	139.6	3563	2927
**240**	947	7550.9	0.304	-0.88	14193	356	364	107436	1359	897	139.6	3548	2988
**260**	868	2918.3	0.304	-0.88	15253	331	352	107429	1288	887	139.6	3431	2886
**280**	700	459	0.304	-0.88	13346	311	347	119019	1178	930	139.6	3435	2925
**300**	545	101	0.304	-0.88	14790	280	336	116984	1131	891	139.6	3331	2907
**320**	405	18.4	0.407	-0.88	16500	257	334	132798	1044	916	139.6	3271	2821
**340**	387	9.8	0.428	-0.88	17863	265	348	131696	1031	886	139.6	3197	2806
**360**	375	4.4	0.440	-0.88	18827	259	347	141225	954	898	139.6	3109	2714
**380**	373	2.2	0.448	-0.88	18994	272	348	151508	931	920	139.6	3167	2736
**400**	323	1.8	0.554	-0.88	20250	265	356	163610	841	904	139.6	3090	2737
**420**	310	1.1	0.600	-0.88	21134	258	367	164907	775	888	139.6	3000	2709
**440**	292	0.8	0.692	-0.88	21992	251	374	171044	734	888	139.6	2914	2673

Supplementary Table 1. ***Fit parameters used in fitting the OKE data****.HN, G_i, and B_i refers to the parameters for the Havriliak-Negami, Gaussian, and Brownian-oscillator fit functions respectively (see Supplementary Note 1).*

T/°C	10^3 G_3	q_3	σ_3	10^3 G_4	q_4	σ_4	10^3 L_5	q_5	γ_5	10^3 G_6	q_6	σ_6	10^3 G_7	q_7	σ_7	10^3 G_8	q_8	σ_8
**25**	0.48	0.67	0.09	3.74	0.76	0.19	10.29	1.40	0.22	0.74	1.52	0.25	2.36	3.06	0.42	27.94	3.13	1.81
**23**	0.49	0.67	0.09	3.70	0.76	0.19	10.55	1.40	0.22	0.63	1.54	0.26	2.46	3.06	0.42	27.83	3.15	1.82
**21**	0.50	0.67	0.09	3.70	0.76	0.19	10.39	1.40	0.22	0.72	1.53	0.26	2.43	3.06	0.42	27.88	3.14	1.81
**19**	0.51	0.67	0.09	3.72	0.76	0.19	10.13	1.40	0.21	0.82	1.52	0.24	2.28	3.06	0.41	27.90	3.12	1.78
**17**	0.50	0.67	0.09	3.71	0.76	0.19	10.51	1.40	0.22	0.67	1.54	0.25	2.32	3.06	0.41	27.75	3.15	1.78
**15**	0.52	0.67	0.09	3.71	0.76	0.19	10.60	1.40	0.22	0.63	1.55	0.25	2.35	3.06	0.42	27.59	3.15	1.77
**13**	0.51	0.67	0.09	3.72	0.76	0.19	10.55	1.40	0.21	0.66	1.55	0.24	2.26	3.06	0.41	27.64	3.15	1.75
**11**	0.53	0.67	0.09	3.67	0.76	0.19	10.57	1.40	0.21	0.66	1.55	0.26	2.33	3.06	0.41	27.65	3.16	1.76
**9**	0.51	0.67	0.09	3.74	0.76	0.19	10.54	1.40	0.21	0.69	1.55	0.25	2.21	3.06	0.40	27.40	3.15	1.72
**7**	0.57	0.67	0.09	3.58	0.76	0.19	10.63	1.41	0.21	0.63	1.56	0.25	2.36	3.06	0.41	27.76	3.16	1.76
**5**	0.52	0.67	0.09	3.68	0.76	0.19	10.74	1.41	0.21	0.63	1.57	0.25	2.35	3.06	0.41	27.45	3.17	1.73
**3**	0.50	0.67	0.09	3.69	0.76	0.19	10.76	1.41	0.21	0.64	1.56	0.25	2.30	3.06	0.41	27.38	3.17	1.72
**1**	0.52	0.67	0.09	3.71	0.76	0.19	10.78	1.41	0.21	0.60	1.58	0.25	2.20	3.06	0.40	27.31	3.17	1.69
**-1**	0.51	0.67	0.09	3.69	0.76	0.19	10.79	1.41	0.21	0.62	1.58	0.25	2.26	3.06	0.41	27.36	3.17	1.70
**-3**	0.56	0.67	0.09	3.64	0.77	0.19	10.70	1.41	0.21	0.66	1.58	0.25	2.21	3.06	0.40	27.38	3.17	1.69
**-5**	0.51	0.67	0.09	3.68	0.76	0.19	10.94	1.41	0.21	0.57	1.59	0.25	2.19	3.06	0.40	27.19	3.18	1.68
**-7**	0.54	0.67	0.09	3.68	0.77	0.19	10.68	1.41	0.21	0.71	1.57	0.25	2.19	3.06	0.40	27.29	3.17	1.67
**-9**	0.56	0.67	0.09	3.70	0.77	0.20	11.00	1.42	0.21	0.58	1.61	0.25	2.22	3.06	0.40	26.91	3.19	1.65
**-11**	0.56	0.67	0.09	3.66	0.77	0.20	10.93	1.42	0.21	0.61	1.60	0.25	2.17	3.06	0.40	26.96	3.18	1.64
**-13**	0.56	0.67	0.09	3.63	0.77	0.19	10.95	1.42	0.21	0.61	1.60	0.24	2.16	3.06	0.39	27.11	3.19	1.65
**-15**	0.55	0.67	0.09	3.68	0.77	0.20	10.83	1.42	0.20	0.68	1.59	0.24	2.13	3.06	0.39	27.00	3.18	1.63
**-17**	0.56	0.67	0.09	3.64	0.77	0.20	10.97	1.42	0.20	0.60	1.61	0.24	2.14	3.06	0.39	26.99	3.18	1.63
**-19**	0.55	0.67	0.09	3.64	0.77	0.19	10.88	1.42	0.20	0.65	1.59	0.24	2.09	3.06	0.39	27.12	3.18	1.63
**-21**	0.56	0.67	0.09	3.68	0.77	0.20	10.99	1.42	0.20	0.63	1.60	0.24	2.01	3.06	0.38	26.79	3.18	1.59
**-23**	0.55	0.67	0.09	3.67	0.77	0.20	11.02	1.42	0.20	0.65	1.61	0.24	2.08	3.06	0.39	26.82	3.19	1.60
**-25**	0.56	0.67	0.09	3.70	0.77	0.20	10.98	1.42	0.20	0.66	1.60	0.24	1.95	3.05	0.38	26.73	3.18	1.57
**-27**	0.54	0.67	0.09	3.70	0.77	0.20	11.14	1.43	0.20	0.61	1.63	0.24	2.08	3.06	0.39	26.63	3.20	1.58
**-29**	0.58	0.67	0.09	3.63	0.77	0.20	11.16	1.43	0.20	0.61	1.63	0.24	2.03	3.05	0.38	26.75	3.20	1.58
**-31**	0.59	0.66	0.09	3.61	0.77	0.20	11.04	1.43	0.20	0.67	1.61	0.24	2.08	3.06	0.39	26.83	3.20	1.58
**-33**	0.57	0.67	0.09	3.66	0.77	0.20	11.17	1.43	0.20	0.63	1.63	0.24	2.04	3.05	0.38	26.60	3.20	1.56
**-35**	0.58	0.66	0.09	3.64	0.77	0.20	11.02	1.43	0.20	0.69	1.61	0.24	1.98	3.06	0.38	26.80	3.19	1.56
**-37**	0.57	0.67	0.09	3.64	0.77	0.20	11.15	1.43	0.20	0.64	1.62	0.24	1.95	3.05	0.37	26.73	3.20	1.55
**-39**	0.56	0.66	0.09	3.67	0.77	0.20	11.21	1.43	0.20	0.64	1.63	0.24	1.96	3.06	0.37	26.62	3.20	1.54
**-41**	0.55	0.67	0.09	3.69	0.77	0.20	11.18	1.43	0.20	0.66	1.63	0.24	1.92	3.05	0.37	26.55	3.20	1.53
**-43**	0.58	0.66	0.09	3.64	0.78	0.20	11.30	1.44	0.20	0.62	1.64	0.24	1.93	3.05	0.37	26.55	3.21	1.53
**-45**	0.62	0.66	0.09	3.61	0.78	0.20	11.20	1.44	0.20	0.68	1.63	0.24	1.92	3.05	0.37	26.58	3.21	1.52
**-47**	0.60	0.66	0.09	3.60	0.78	0.20	11.27	1.44	0.20	0.65	1.64	0.24	1.99	3.05	0.38	26.59	3.22	1.53
**-49**	0.61	0.66	0.09	3.62	0.78	0.20	11.32	1.44	0.20	0.69	1.65	0.25	1.99	3.05	0.38	26.40	3.22	1.51
**-51**	0.63	0.66	0.09	3.56	0.78	0.20	11.23	1.44	0.19	0.67	1.64	0.24	1.91	3.05	0.37	26.69	3.21	1.52
**-53**	0.63	0.66	0.09	3.51	0.78	0.20	11.25	1.44	0.19	0.68	1.64	0.24	2.07	3.06	0.38	26.73	3.22	1.54
**-55**	0.61	0.66	0.09	3.62	0.78	0.20	11.23	1.44	0.19	0.74	1.64	0.24	1.93	3.05	0.37	26.47	3.22	1.50

**-57**	0.63	0.66	0.09	3.59	0.78	0.20	11.33	1.44	0.19	0.69	1.65	0.24	1.90	3.05	0.37	26.47	3.22	1.49
**-59**	0.63	0.66	0.09	3.60	0.78	0.20	11.24	1.44	0.19	0.73	1.64	0.24	1.86	3.05	0.36	26.52	3.21	1.48
**-61**	0.64	0.66	0.09	3.56	0.78	0.20	11.34	1.45	0.19	0.72	1.65	0.24	1.95	3.05	0.37	26.47	3.23	1.49
**-63**	0.64	0.66	0.09	3.58	0.78	0.20	11.37	1.45	0.19	0.72	1.66	0.24	1.97	3.05	0.37	26.38	3.23	1.48
**-65**	0.65	0.66	0.09	3.56	0.78	0.20	11.49	1.45	0.19	0.69	1.67	0.25	1.94	3.05	0.37	26.35	3.24	1.48
**-67**	0.69	0.66	0.09	3.46	0.79	0.20	11.42	1.45	0.19	0.70	1.66	0.25	2.06	3.05	0.38	26.57	3.24	1.50
**-69**	0.68	0.66	0.09	3.48	0.79	0.20	11.31	1.45	0.19	0.74	1.65	0.24	1.94	3.05	0.37	26.67	3.23	1.49
**-71**	0.67	0.66	0.09	3.52	0.79	0.20	11.40	1.45	0.19	0.76	1.66	0.25	2.00	3.05	0.37	26.39	3.24	1.48
**-73**	0.67	0.66	0.09	3.55	0.79	0.20	11.36	1.45	0.19	0.79	1.65	0.24	1.89	3.05	0.36	26.38	3.23	1.46
**-75**	0.71	0.66	0.10	3.47	0.79	0.20	11.36	1.45	0.19	0.77	1.65	0.24	1.93	3.05	0.37	26.49	3.23	1.47
**-77**	0.68	0.66	0.09	3.50	0.79	0.20	11.45	1.46	0.19	0.77	1.66	0.24	1.92	3.05	0.37	26.40	3.24	1.46
**-79**	0.71	0.66	0.09	3.51	0.79	0.20	11.42	1.46	0.18	0.77	1.66	0.24	1.84	3.05	0.36	26.37	3.24	1.44
**-81**	0.72	0.66	0.09	3.45	0.79	0.20	11.33	1.46	0.18	0.80	1.65	0.24	1.87	3.05	0.36	26.58	3.23	1.46
**-83**	0.72	0.66	0.10	3.48	0.79	0.20	11.51	1.46	0.18	0.76	1.67	0.24	1.92	3.05	0.36	26.29	3.25	1.44
**-85**	0.73	0.66	0.10	3.44	0.79	0.20	11.48	1.46	0.18	0.78	1.67	0.24	1.97	3.05	0.36	26.41	3.25	1.45
**-87**	0.73	0.66	0.10	3.44	0.79	0.20	11.46	1.46	0.18	0.79	1.67	0.24	1.85	3.05	0.35	26.47	3.24	1.44
**-89**	0.71	0.66	0.09	3.50	0.79	0.20	11.59	1.46	0.18	0.79	1.68	0.25	1.90	3.04	0.36	26.15	3.25	1.42
**-91**	0.75	0.66	0.10	3.43	0.79	0.20	11.56	1.46	0.18	0.80	1.67	0.24	1.92	3.05	0.36	26.29	3.25	1.43
**-93**	0.74	0.66	0.09	3.44	0.79	0.20	11.51	1.47	0.18	0.81	1.67	0.24	1.88	3.05	0.36	26.41	3.25	1.43
**-95**	0.75	0.66	0.09	3.42	0.80	0.20	11.50	1.47	0.18	0.83	1.67	0.24	1.88	3.05	0.35	26.41	3.25	1.42
**-97**	0.77	0.66	0.10	3.39	0.80	0.20	11.52	1.47	0.18	0.82	1.67	0.24	1.92	3.05	0.36	26.48	3.26	1.43
**-99**	0.78	0.66	0.10	3.40	0.80	0.21	11.58	1.47	0.18	0.83	1.68	0.24	1.98	3.05	0.36	26.31	3.26	1.42
**-101**	0.78	0.66	0.10	3.40	0.80	0.21	11.50	1.47	0.18	0.85	1.67	0.24	1.85	3.05	0.35	26.43	3.25	1.41
**-103**	0.82	0.66	0.10	3.35	0.80	0.21	11.55	1.47	0.18	0.85	1.68	0.24	1.94	3.05	0.36	26.44	3.26	1.42
**-105**	0.84	0.65	0.10	3.29	0.80	0.21	11.61	1.47	0.18	0.83	1.69	0.24	2.04	3.05	0.36	26.49	3.27	1.43
**-107**	0.83	0.65	0.10	3.30	0.80	0.21	11.63	1.48	0.18	0.83	1.69	0.24	2.05	3.05	0.37	26.45	3.27	1.43
**-109**	0.84	0.65	0.10	3.31	0.80	0.21	11.67	1.48	0.18	0.84	1.69	0.24	2.03	3.05	0.36	26.40	3.28	1.42
**-111**	0.85	0.65	0.10	3.28	0.80	0.20	11.67	1.48	0.18	0.84	1.69	0.24	2.04	3.05	0.36	26.46	3.28	1.42
**-113**	0.85	0.65	0.10	3.28	0.80	0.21	11.66	1.48	0.18	0.83	1.69	0.24	2.04	3.05	0.36	26.49	3.28	1.42
**-115**	0.85	0.65	0.10	3.27	0.80	0.21	11.84	1.48	0.18	0.84	1.71	0.25	2.21	3.05	0.37	26.31	3.30	1.43
**-117**	0.86	0.65	0.10	3.25	0.81	0.21	11.68	1.48	0.17	0.85	1.68	0.24	2.02	3.05	0.36	26.55	3.28	1.42
**-119**	0.86	0.65	0.10	3.30	0.80	0.21	11.80	1.48	0.17	0.86	1.70	0.24	2.07	3.05	0.36	26.30	3.29	1.40
**-121**	0.87	0.65	0.10	3.26	0.81	0.21	11.84	1.49	0.17	0.85	1.70	0.24	2.11	3.05	0.36	26.31	3.30	1.40
**-123**	0.89	0.65	0.10	3.21	0.81	0.21	11.85	1.49	0.17	0.85	1.71	0.25	2.19	3.05	0.37	26.41	3.30	1.42
**-125**	0.85	0.65	0.10	3.32	0.81	0.21	11.83	1.49	0.17	0.89	1.70	0.25	2.06	3.05	0.36	26.26	3.30	1.39
**-127**	0.89	0.65	0.10	3.21	0.81	0.21	11.82	1.49	0.17	0.86	1.70	0.24	2.11	3.05	0.36	26.50	3.30	1.41
**-129**	0.90	0.65	0.10	3.19	0.81	0.21	11.93	1.49	0.17	0.84	1.71	0.24	2.13	3.05	0.36	26.40	3.30	1.40
**-131**	0.94	0.65	0.10	3.16	0.81	0.21	11.84	1.49	0.17	0.88	1.70	0.24	2.16	3.06	0.36	26.43	3.30	1.40
**-133**	0.93	0.65	0.10	3.17	0.81	0.21	11.87	1.49	0.17	0.89	1.71	0.24	2.16	3.05	0.36	26.37	3.30	1.40
**-135**	0.93	0.65	0.10	3.20	0.81	0.21	11.95	1.50	0.17	0.91	1.72	0.25	2.24	3.05	0.36	26.21	3.32	1.40
**-137**	0.94	0.65	0.10	3.19	0.81	0.21	11.97	1.50	0.17	0.89	1.71	0.24	2.19	3.05	0.36	26.18	3.31	1.39
**-139**	0.98	0.65	0.10	3.11	0.82	0.21	11.98	1.50	0.17	0.88	1.72	0.25	2.24	3.05	0.36	26.29	3.31	1.40
**-141**	1.02	0.64	0.10	3.02	0.82	0.21	11.96	1.50	0.17	0.85	1.71	0.24	2.25	3.06	0.36	26.52	3.31	1.41

**-143**	0.99	0.64	0.10	3.12	0.82	0.21	12.11	1.50	0.17	0.89	1.73	0.25	2.30	3.05	0.36	26.12	3.33	1.39
**-145**	0.98	0.64	0.10	3.15	0.81	0.21	12.25	1.50	0.17	0.92	1.75	0.26	2.54	3.05	0.38	25.88	3.35	1.40
**-147**	1.02	0.64	0.10	3.04	0.82	0.21	12.06	1.51	0.17	0.90	1.72	0.25	2.36	3.05	0.37	26.30	3.33	1.40
**-149**	0.95	0.64	0.10	3.26	0.81	0.21	12.37	1.51	0.17	1.09	1.76	0.28	2.84	3.04	0.40	25.22	3.40	1.39
**-151**	1.01	0.64	0.10	3.10	0.82	0.21	12.19	1.51	0.17	0.92	1.74	0.25	2.41	3.05	0.37	26.05	3.34	1.39
**-153**	0.99	0.64	0.10	3.13	0.82	0.21	12.30	1.51	0.17	0.96	1.75	0.26	2.60	3.05	0.38	25.77	3.37	1.39
**-155**	1.01	0.64	0.10	3.10	0.82	0.21	12.28	1.51	0.17	0.93	1.74	0.26	2.51	3.05	0.37	25.88	3.36	1.38
**-157**	1.00	0.64	0.10	3.15	0.81	0.21	12.47	1.51	0.17	1.02	1.77	0.28	2.86	3.04	0.39	25.38	3.40	1.39
**-159**	0.99	0.64	0.10	3.22	0.81	0.22	12.59	1.51	0.17	1.13	1.79	0.29	3.18	3.04	0.41	24.87	3.44	1.39
**-161**	1.05	0.64	0.10	3.08	0.82	0.21	12.47	1.52	0.17	1.07	1.78	0.28	3.04	3.04	0.40	25.33	3.42	1.41
**-163**	1.04	0.64	0.10	3.10	0.82	0.21	12.49	1.52	0.17	1.03	1.77	0.28	2.86	3.05	0.39	25.27	3.40	1.38
**-165**	1.01	0.64	0.10	3.15	0.82	0.22	12.60	1.52	0.17	1.07	1.79	-0.28	3.04	3.04	0.40	25.03	3.43	1.38
**-167**	1.03	0.64	0.10	3.10	0.82	0.21	12.46	1.52	0.17	1.03	1.77	-0.28	2.73	3.05	0.38	25.34	3.39	1.37
**-169**	1.03	0.64	0.10	3.10	0.82	0.21	12.56	1.52	0.17	1.05	1.78	-0.28	2.96	3.05	0.39	25.18	3.42	1.38
**-171**	1.01	0.64	0.10	3.14	0.82	0.21	12.58	1.52	0.17	1.08	1.78	-0.28	2.96	3.04	0.39	25.04	3.42	1.37
**-173**	1.02	0.64	0.10	3.10	0.82	0.21	12.59	1.52	0.17	1.05	1.79	-0.28	2.93	3.05	0.39	25.22	3.42	1.38
**-175**	1.05	0.64	0.10	3.03	0.82	0.21	12.58	1.52	0.17	1.00	1.78	-0.27	2.87	3.05	0.39	25.37	3.40	1.38
**-177**	1.05	0.64	0.10	3.00	0.82	0.21	12.48	1.52	0.17	0.95	1.76	-0.27	2.64	3.06	0.37	25.67	3.38	1.38
**-179**	1.01	0.64	0.10	3.09	0.82	0.21	12.57	1.52	0.17	1.03	1.78	-0.27	2.93	3.05	0.39	25.29	3.41	1.38

Supplementary Table 2. *Fit parameters for the temperature-dependent WAXS data. The meaning of the fit parameters is explained in Supplementary Note 2.*

**10^3 P_1**	38.40	**10^3 G_2**	0.020	**10^3 G_3**	1.368	**10^3 G_4**	0.521	**10^3 L_5**	6.435	**10^3 G_6**	0.477	**10^3 G_7**	2.550	**10^3 G_8**	9.947
**N**	3.62	**q_2**	0.234	**q_3**	0.681	**q_4**	0.988	**q_5**	1.521	**q_6**	1.927	**q_7**	3.022	**q_8**	3.787
		σ_2	0.063	σ_3	0.144	σ_4	0.128	γ_5	0.181	σ_6	0.263	σ_7	0.498	σ_8	1.445

Supplementary Table 3. *Fit parameters WAXS data averaged for temperature ≤ 110 K. The meaning of the fit parameters is explained in Supplementary Note 2.*

Voronoi index	CN	Notes
**Icosahedral (ICO)**		
< 0, 0, 12, 0 >	12	Icosahedron
< 0, 1, 10, 2 >	13	Defective icosahedron [22]
< 0, 2, 8, 1 >	11	Z11 Kasper polyhedron [23]
< 0, 2, 8, 2 >	12	Ref.[24]
< 0, 2, 8, 3 >	13	Ref.[25]
< 0, 1, 8, 3 >	12	Ref.[26]
< 0, 0, 10, 2 >	12	Ref. [26]
< 0, 3, 6, 3 >	12	Ref.[27]
< 0, 4, 4, 3 >	11	Based on < 0, 2, 8, 1 >, with defects in polytetrahedral packing [25]
< 0, 4, 4, 4 >	12	Ref.[27]
< 0, 4, 4, 5 >	13	Ref.[25]
**FCC**		
< 0, 12, 0, 0 >	12	Rhombic dodecahedron
< 0, 3, 6, 4 > [FCC-ICO]	13	Z13 Kasper polyhedron [22]<br>Occasionally labelled as indicative of ICO topology as well [28]
< 0, 3, 6, 5 >	14	Z14 Frank-Kasper polyhedron[25]
< 0, 4, 4, 7 >	15	Ref. [27]
**BCC**		
< 0, 6, 8, 0 >	14	Truncated octahedron
< 0, 2, 8, 4 >	14	Frank-Kasper polyhedron. Ref. [27]
< 0, 4, 4, 6 >	14	Z14 Kasper polyhedron. Distorted body-centred cubic crystalline clusters [27]
< 0, 5, 2, 6 >	13	Distorted body-centred cubic crystalline clusters [29]
**HCP**		
< 0, 12, 0, 0 >	12	Trapezo-rhombic dodecahedron
< 0, 4, 4, 2 >	10	Ref. [30]
**Prism**		
< 0, 3, 6, 0 >	9	Tri-capped trigonal prism (TTP) [31]
< 0, 2, 8, 0 >	10	Bi-capped square Archimedean antiprism (BSAP) [31]
< 0, 4, 4, 0 >	8	Tetragonal dodecahedron [31]
< 0, 4, 4, 1 >	9	Distorted TTP and BSAP [25]
< 0, 3, 6, 1 >	10	Distorted TTP and BSAP [25]
**Frank-Kasper**[32]		
< 0, 2, 8, 5 >	15	Ref. [25]
< 0, 3, 6, 6 >	15	Ref. [25]
< 0, 4, 5, 4, 1 >	15	Ref. [25]
< 0, 3, 7, 4, 1 >	15	Ref. [25]
**Mix**		
< 0, 3, 6, 2 >	11	Based on < 0, 2, 8, 1 >, with defects in polytetrahedral packing [25]
**Frequently observed in TBOS**		
< 0, 2, 5, 4 >	11	Relevant to Al-Cu alloys [26]
< 0, 4, 4, 3, 3 >	14	
< 0, 4, 6, 4, 2 >	16	
< 0, 4, 6, 2, 2, 1 >	15	

Supplementary Table 4. *Voronoi indices for selected Voronoi Polyhedra (VP), grouped according to different classes: VP characteristic of icosahedral order (ICO), VP characteristic of crystalline order (FCC, BCC and HCP), prism VP, Frank-Kasper VP (relevant in the context of intermetallic compounds), and uncategorised VP that are frequently observed in our models of TBOS. CN refers to the coordination number relative to each VP.*

Supplementary References

1. Reichenbach, J. *et al.* Phonon-like Hydrogen-Bond Modes in Protic Ionic Liquids. *J. Am. Chem. Soc.* **139**, 7160–7163 (2017).
2. Cho, M., Du, M., Scherer, N. F., Fleming, G. R. & Mukamel, S. Off-resonant transient birefringence in liquids. *J Chem Phys* **99**, 19 (1993).
3. Turton, D. A. *et al.* Terahertz underdamped vibrational motion governs protein-ligand binding in solution. *Nat. Commun.* **5**, 3999 (2014).
4. Daisenberger, D. *et al.* Polyamorphic Amorphous Silicon at High Pressure: Raman and Spatially Resolved X-ray Scattering and Molecular Dynamics Studies. *J. Phys. Chem. B* **115**, 14246–14255 (2011).
5. Martínez, L., Andrade, R., Birgin, E. G. & Martínez, J. M. PACKMOL: A package for building initial configurations for molecular dynamics simulations. *J. Comput. Chem.* **30**, 2157–2164 (2009).
6. Martínez, J. M. & Martínez, L. Packing optimization for automated generation of complex system's initial configurations for molecular dynamics and docking. *J. Comput. Chem.* **24**, 819–825 (2003).
7. Mizuguchi, T., Tatsumi, S. & Fujiwara, S. Icosahedral order in liquid and glassy phases of cyclohexane. *Mol. Simul.* **46**, 721–726 (2020).
8. Fukunaga, T. *et al.* Voronoi Analysis of the Structure of Ni-Zr-Al Ternary Metallic Glass. *Mater. Trans.* **48**, 1698–1702 (2007).
9. Brostow, W., Chybicki, M., Laskowski, R. & Rybicki, J. Voronoi polyhedra and Delaunay simplexes in the structural analysis of molecular-dynamics-simulated materials. *Phys. Rev. B* **57**, 13448–13458 (1998).
10. Mosayebi, M. *et al.* Beyond icosahedral symmetry in packings of proteins in spherical shells. *Proc. Natl. Acad. Sci.* **114**, 9014–9019 (2017).
11. Lechner, W. & Dellago, C. Accurate determination of crystal structures based on averaged local bond order parameters. *J. Chem. Phys.* **129**, 114707 (2008).
12. Giberti, F., Salvalaglio, M., Mazzotti, M. & Parrinello, M. Insight into the nucleation of urea crystals from the melt. *Chem. Eng. Sci.* **121**, 51–59 (2015).
13. Yannopoulos, S. N., Andrikopoulos, K. S. & Ruocco, G. On the analysis of the vibrational Boson peak and low-energy excitations in glasses. *J. Non-Cryst. Solids* **352**, 4541–4551 (2006).
14. Orsingher, L., Baldi, G., Fontana, A. & Rossi, F. The Raman coupling function in permanently densified GeO2 glasses. *Philos. Mag.* **91**, 1857–1863 (2011).
15. Fontana, A. *et al.* The Raman coupling function in amorphous silica and the nature of the long-wavelength excitations in disordered systems. *EPL Europhys. Lett.* **47**, 56 (1999).
16. Achibat, T., Boukenter, A. & Duval, E. Correlation effects on Raman scattering from low-energy vibrational modes in glasses. II. Experimental results. *J. Chem. Phys.* **99**, 2046–2051 (1993).
17. Mantisi, B. *et al.* Non-Debye normalization of the glass vibrational density of states in mildly densified silicate glasses. *J. Phys. Condens. Matter* **22**, 025402 (2010).
18. Deschamps, T., Martinet, C., de Ligny, D., Bruneel, J. L. & Champagnon, B. Low-frequency Raman scattering under high pressure in diamond anvil cell: Experimental protocol and application to GeO_2 and SiO_2 boson peaks. *J. Non-Cryst. Solids* **358**, 3156–3160 (2012).
19. Schroeder, J. *et al.* Raman scattering and Boson peaks in glasses: temperature and pressure effects. *J. Non-Cryst. Solids* **349**, 88–97 (2004).
20. Weeraman, C., Yatawara, A. K., Bordenyuk, A. N. & Benderskii, A. V. Effect of Nanoscale Geometry on Molecular Conformation: Vibrational Sum-Frequency Generation of Alkanethiols on Gold Nanoparticles. *J. Am. Chem. Soc.* **128**, 14244–14245 (2006).
21. Brown, K. G., Bicknell-Brown, Ellen. & Ladjadj, Meriem. Raman-active bands sensitive to motion and conformation at the chain termini and backbones of alkanes and lipids. *J. Phys. Chem.* **91**, 3436–3442 (1987).
22. Mizuguchi, T., Tatsumi, S. & Fujiwara, S. Icosahedral order in liquid and glassy phases of cyclohexane. *Mol. Simul.* **46**, 721–726 (2020).
23. Sheng, H. W., Luo, W. K., Alamgir, F. M., Bai, J. M. & Ma, E. Atomic packing and short-to-medium-range order in metallic glasses. *Nature* **439**, 419–425 (2006).
24. Guo, G. Quasi-Icosahedral Clusters in Zr-Based Metallic Glasses. *Metals* **10**, 1135 (2020).
25. Zhang, H., Sun, H., Li, Q. & Wang, L. Origin of Local Structures of U-Co Melts: A First-Principles Study. *Front. Mater.* **8**, (2022).
26. Zhang, Q., Li, J., Tang, S., Wang, Z. & Wang, J. Atomistic Mechanism Underlying Nucleation in Al–Cu Alloys with Different Compositions and Cooling Rates. *J. Phys. Chem. C* **125**, 3480–3494 (2021).
27. Şengül, S. & Çeltek, M. Pressure Effects on the Structural Evolution of Monatomic Metallic Liquid Hafnium. *Bitlis Eren Üniversitesi Fen Bilim. Derg.* 144–158 (2018) doi:10.17798/bitlisfen.416922.
28. Ren, L., Gao, T., Ma, R., Xie, Q. & Hu, X. The icosahedral short-range order and its local structures in $Cu_{50}Zr_{40}Al_{10}$ alloy. *Mater. Res. Express* **6**, 016510 (2018).
29. Han, J. *et al.* Atomic-Level Mechanisms of Nucleation of Pure Liquid Metals during Rapid Cooling. *ChemPhysChem* **16**, 3916–3927 (2015).

30. Durandurdu, M. MgCu metallic glass. *Philos. Mag.* **98**, 633–645 (2018).
31. Fukunaga, T. *et al.* Voronoi Analysis of the Structure of Ni-Zr-Al Ternary Metallic Glass. *Mater. Trans.* **48**, 1698–1702 (2007).
32. Frank, F. C. & Kasper, J. S. Complex alloy structures regarded as sphere packings. I. Definitions and basic principles. *Acta Crystallogr.* **11**, 184–190 (1958).
