# Supplementary material for: Understanding the emergence of the boson peak in molecular glasses
Source: Nat Commun. 2023 Jan 13;14:215. doi: 10.1038/s41467-023-35878-6 (PMC9839737; doi:10.1038/s41467-023-35878-6)
Supplement: Supplementary file 2 — Peer Review File [file 41467_2023_35878_MOESM2_ESM.pdf]

nature portfolio

Peer Review File

Understanding the emergence of the boson peak in molecular glasses

Open Access This file is licensed under a Creative Commons Attribution 4.0 International License, which permits use, sharing, adaptation, distribution and reproduction in any medium or format, as long as you give appropriate credit to the original author(s) and the source, provide a link to the Creative Commons license, and indicate if changes were made. In the cases where the authors are anonymous, such as is the case for the reports of anonymous peer reviewers, author attribution should be to 'Anonymous Referee' followed by a clear attribution to the source work. The images or other third party material in this file are included in the article's Creative Commons license, unless indicated otherwise in a credit line to the material. If material is not included in the article's Creative Commons license and your intended use is not permitted by statutory regulation or exceeds the permitted use, you will need to obtain permission directly from the copyright holder. To view a copy of this license, visit http://creativecommons.org/licenses/by/4.0/.

REVIEWER COMMENTS

Reviewer #1 (Remarks to the Author):

Summary
In this paper is examined the origin of the boson peak using a prototypical molecular glass former, tetrabutyl orthosilicate (TBOS). The experimental methods consist of optical Kerr effect (OKE) spectroscopy as well as wide- and small-angle x-ray scattering (SAXS,WAXS) measurements, complemented by other several other measurements (such as calorimetry, NMR, FTIR, Raman, viscometry) and simulations (MD and DFT). The main results relate to an excess temperature dependent intensity in the <3 THz region of the Raman spectrum, probed by OKE. The spectrum is analysed in terms of inter- and intramolecular components; the former consists of a high-frequency band at ~2THz attributed to acoustic phonons and a second low-frequency band ~0.9 THz attributed to the boson peak. The SAXS/WAXS measurements indicate the existence of a pre-peak at ~0.234 /Å which is attributed to the formation of molecular clusters corresponding to distances of ~26.9 Å. The MD simulations reproduce well both the OKE and SAXS/WAXS results, whereas Voronoi analysis indicates that below Tg = 124 K (estimated by calorimetry) emerge clusters with BCC local order. In conclusion, the observed band attributed to the boson peak is associated with the formation of over-coordinated clusters which can prevent crystallization.

Assessment
I find the paper well written, with clarity of presentation and rich as well as convincing results. I liked the described scheme for using probing the boson peak by the OKE approach by selectivity through symmetry of the selected system (here near tetrahedral), which allows to exclude rotational contributions (confirmed by DFT). I also find convincing that this assignment is confirmed by low-temperature calorimetry which shows an excess heat capacity at 6K. The results are of very high quality, including a nice comparison between the OKE measurements with WAXS measurements. The additional measurements are almost overwhelming, although useful for strengthening the interpretation of transient cluster formation while at the same time showing that the sample remains monomeric, as confirmed by NMR and DFT. In the results is also included MD simulations that allow an interesting insight into the molecular coordination of the clusters, which are mainly icosahedral (at any given temperature) and truncated octahedral (appearing at T_g) . Understanding the origin of the boson peak can be a of great interest for many in the field and gives a promising insight for understanding the link of collective oscillations due to “cage rattling” modes which manifest in the supercooled region, which can inhibit crystallization.

I therefore would support the manuscript’s publication in Nature Communications after the following comments have been considered.

Comments

1. I was wondering about the interpretation of the black line shown in Fig. 1b, which shows a pronounced temperature dependence as indicated in Suppl. Fig. 6. I missed assignment of this peak in the text, even though this is the range with the most pronounced T-dependence (at least in the log-x representation).

2. I have a few comments related to the pre-peak indicated in the WAXS at ~0.234 /Å:

2a. As far as I understand the WAXS and SAXS spectra are “stitched together” just below ~0.2 /Å, so it would be good to specify the exact q-range in the methods section.

2b. I am a bit concerned about the influence of the background either from the capillary or the direct beam. It is mentioned that plastic capillaries were used, so it would be useful to plot the SAXS/WAXS background from the capillaries (without any sample) to ensure that there is no matching peak at this momentum transfer range.

2c. Suppl. Fig. 8 needs some additional care. It would be helpful to make the figure labels visible, include proper legends and also re-bin (using larger q-bins) the SAXS data to improve the readability of the data in the q-range above 0.01/Å.

3. In connection to the previous comment, I am also wondering about the pre-peak observed S(q) simulations results shown in Fig. 4b and Suppl. Fig. 14:

3a. While indeed as it is pointed out in the paper, the pre-peak is reproduced in the Si-Si S(q), similar peaks in the region q<1/Å with even stronger temperature dependence are shown in the S(q)’s of C-O, C-Si and also in the O-O. Do these temperature-dependent signals reflect changes intermolecular or intramolecular distances? I.e. it should be specified whether in the calculation is included atoms within the same molecule or only intermolecular distances.

3b. It might be helpful to plot all these results in Suppl. Fig. 14 with logarithmic x-axis (as in Fig. 4b).

3c. The peak at q~0.2 /Å is attributed to clusters with size ~2π/q = 26.9 Å. Would then one not expect a similar peak in the Si-Si PCF at this range (Suppl. Fig.15)? Or is this range limited by the simulation box size?

4. Concerning the Voronoi analysis:

4a. It would be helpful to include some additional explanations concerning the formalism used in the corresponding indices and how one can derive the corresponding symmetry.

4b. It would be interesting to include a schematic of the Si-SI network and polyhedral derived from the simulations to facilitate the interpretation.

4c. The analysis indicates that network of the Si atoms exhibits icosahedral coordination at any given temperature, whereas the observed truncated octahedra emerge near the T_g. One of the main conclusions of the paper is however that the observed transient clusters can inhibit crystallization. According to this interpretation, wouldn’t one expect that the cluster formation should emerge in the supercooled regime?

Reviewer #2 (Remarks to the Author):

The work by González-Jiménez studied the dynamical properties, i.e. boson peak, of a molecular glass by combining experiments and simulations. A link to the local structure is

suggested. However, this paper is more technical relevant with lots of assumptions involved in the analysis, so it is not convincing to me. Therefore, I cannot recommend its publication in Nat. Commun., a more specialized journal may be more suitable. See some detailed comments below.
1) The introduction should be carefully re-organized to target the problem to be solved in this paper. For example, the main results are related to boson peak and glass structure, but the introduction mainly focused on different relaxation modes, like beta relaxation. Not many details about the boson peak are shown.
2) In the introduction, “Below the glass-transition, secondary or β-relaxation processes occur due to faster dynamic processes” may not be necessarily true. I believe different relaxation modes should coexist always in a thermodynamic state, but their observation will depend on the experimental time scale.
3) There is a contradiction about the origin of the secondary relaxations in the introduction, whether intramolecular or intermolecular.
4) The detailed data analysis from the experimental spectra depends on the assumption that they will be simplified for some molecules with ordinary symmetry, tetrahedral and octahedral. This has not been rationalized. Also, in the glass state, there are enormous types of these local structures with different distortions, how would this fact influence the results?
5) The spectrum fitting with four functions of many free parameters seems highly possible to bring over-fitting. This is very critical for the overall interpretation of the results.
6) The structure analysis by pair-correlation function and the Voronoi tessellation does not account for the orientation information in the local structures, which should be important in this molecular glass.
7) The direct comparison of the simulations by a simplified model with the experiments is not convincing. The consistency of the partial structure factor only shows the overall averaged information similarly, not necessarily mean the model it’s correct.
8) At elevated temperatures, anharmonicity is important, which should have been into consideration in the spectrum analysis.

Reviewer #3 (Remarks to the Author):

The manuscript of González-Jiménez et al. entitled "Understanding the emergence of the boson peak in molecular glasses" aims at unveiling the boson peak in a highly symmetric glass former using femtosecond optical Kerr-effect spectroscopy as the central characterization technique, at different temperatures. They then use other experimental techniques (WAXS, SAXS, Raman) and simulation to characterize this boson peak. It is because the characterized molecule, TBOS, is symmetric that the authors can tackle this longstanding problem of the origin of the boson peak.
Two intermolecular bands in the OKE spectrum have been mainly analyzed. In Figure 1 d, the amplitudes of both bands are shown versus the temperature, and in Figure 2 a, their ratio is displayed. Through theses analyses, the authors assign the low frequency band as the boson peak, and the high frequency band as the acoustic phonon band. Thanks to this assignment, they surmised that the boson peak is associated with the formation of clusters of TBOS molecules undergoing collective oscillation. WAXS experiments lead to a distance of 26.9 Angstrom for clusters, and from MD simulation, 25.1 Angstroms. Conclusions drawn from the OKE experiments can have been made because of the symmetric nature of the TBOS molecule. Regarding the over-coordinated molecules in the clusters, deduced from MD, the authors wrote: “the MD simulations are strongly suggestive that these structures are

clusters of over-coordinated TBOS molecules”. For an article in Nature Communications, the authors must be more convincing. For instance, as the molecular behavior can be probed, the over-coordination can be proved.
Other issues.
p.3: It is written “The calculated infrared spectrum in the Si-O-C stretch region (around 1,100 cm-1) matches the experimental one (see Supplementary Figure 3)”. Only the 1000-1200 cm-1 spectrum should be considered. A great part of the simulated spectrum does not fit the experimental one. The authors should indicate why the fit works only for the region of interest.
p.4: the Supplementary Figure 9 does not correspond to the liquid density as outlined in the text.
Indicate what are q3 and q5.
Below 110 K: the value seems to be 110 K.
It is written that “the TBOS molecules reduce the number of gauche defects in the alkoxide side chains”. Such a behavior should be observed in MD.
p.5: The authors consider the two coordination shells in the RDF, computing 2 and 12 molecules respectively. According to these spectra, these numbers should change with the temperature. Some relevant results can be obtained.
p.9: MD simulation part.
When the authors consider random positions within a cubic box: what is the random algorithm used? It is necessary to specify that the periodic boundary conditions have been applied.
Simulations are performed in the NPT ensemble, meaning that a thermostat and a barostat are needed simultaneously. So, why the authors wrote: “to sample the canonical ensemble”?
The authors wrote: “In this regime, the self-diffusion is sufficient to sample a reasonable portion of the configurational space for the liquid phase”. One point in the configurational space corresponds to 87,552 coordinates. What do the authors mean with this reasonable portion?

Finally, how can the study presented in this paper be correlated with the recent paper in Nature Physics on the origin of the boson peak?

Reply to reviewer comments

Reviewer #1 (Remarks to the Author):

Summary

In this paper is examined the origin of the boson peak using a prototypical molecular glass former, tetrabutyl orthosilicate (TBOS). The experimental methods consist of optical Kerr effect (OKE) spectroscopy as well as wide- and small-angle x-ray scattering (SAXS,WAXS) measurements, complemented by other several other measurements (such as calorimetry, NMR, FTIR, Raman, viscometry) and simulations (MD and DFT). The main results relate to an excess temperature dependent intensity in the <3 THz region of the Raman spectrum, probed by OKE. The spectrum is analysed in terms of inter- and intramolecular components; the former consists of a high-frequency band at ~2THz attributed to acoustic phonons and a second low-frequency band ~0.9 THz attributed to the boson peak. The SAXS/WAXS measurements indicate the existence of a pre-peak at ~0.234 /Å which is attributed to the formation of molecular clusters corresponding to distances of ~26.9 Å. The MD simulations reproduce well both the OKE and SAXS/WAXS results, whereas Voronoi analysis indicates that below Tg = 124 K (estimated by calorimetry) emerge clusters with BCC local order. In conclusion, the observed band attributed to the boson peak is associated with the formation of over-coordinated clusters which can prevent crystallization.

Assessment

I find the paper well written, with clarity of presentation and rich as well as convincing results. I liked the described scheme for using probing the boson peak by the OKE approach by selectivity through symmetry of the selected system (here near tetrahedral), which allows to exclude rotational contributions (confirmed by DFT). I also find convincing that this assignment is confirmed by low-temperature calorimetry which shows an excess heat capacity at 6K. The results are of very high quality, including a nice comparison between the OKE measurements with WAXS measurements. The additional measurements are almost overwhelming, although useful for strengthening the interpretation of transient cluster formation while at the same time showing that the sample remains monomeric, as confirmed by NMR and DFT. In the results is also included MD simulations that allow an interesting insight into the molecular coordination of the clusters, which are mainly icosahedral (at any given temperature) and truncated octahedral (appearing at T_g) . Understanding the origin of the boson peak can be a of great interest for many in the field and gives a promising insight for understanding the link of collective oscillations due to "cage rattling" modes which manifest in the supercooled region, which can inhibit crystallization.

We thank the reviewer for the very supportive comments.

I therefore would support the manuscript's publication in Nature Communications after the following comments have been considered.

Comments

1. I was wondering about the interpretation of the black line shown in Fig. 1b, which shows a pronounced temperature dependence as indicated in Suppl. Fig. 6. I missed assignment of this peak in the text, even though this is the range with the most pronounced T-dependence (at least in the log-x representation).

Presumably the confusion has arisen because of the figure caption. We have added a line to the figure caption explaining that the black line is due to diffusive α relaxation. The main text already contains a paragraph (starting with "The spectra in this low frequency range […]") most of which is devoted to diffusive α relaxation and linking it to the measured temperature-dependent shear viscosity.

2. I have a few comments related to the pre-peak indicated in the WAXS at ~0.234 /Å:

2a. As far as I understand the WAXS and SAXS spectra are "stitched together" just below ~0.2 /Å, so it would be good to specify the exact q-range in the methods section.

This is a very good point. The data were stitched together using a single scaling factor for all the SAXS data to produce a good match in the range q = 0.157 to 0.182 Å^{-1} (*i.e.*, just outside of the range of the boson peak feature). This is now described in some detail in the Methods section.

2b. I am a bit concerned about the influence of the background either from the capillary or the direct beam. It is mentioned that plastic capillaries were used, so it would be useful to plot the SAXS/WAXS background from the capillaries (without any sample) to ensure that there is no matching peak at this momentum transfer range.

The graph below shows the background compared to the signal (and fit) at low temperature showing the boson-peak feature. The amorphous polycarbonate capillary has a relatively low scattering cross section. Most importantly, it is flat in the region around 0.25 Å^{-1}. The sharp peak at 0.29 Å^{-1} is due to scattering from the mica windows on the Linkam stage, which remain at room temperature, and is therefore not seen to change with temperature. We are therefore confident that this feature is not caused by scattering from the capillary.

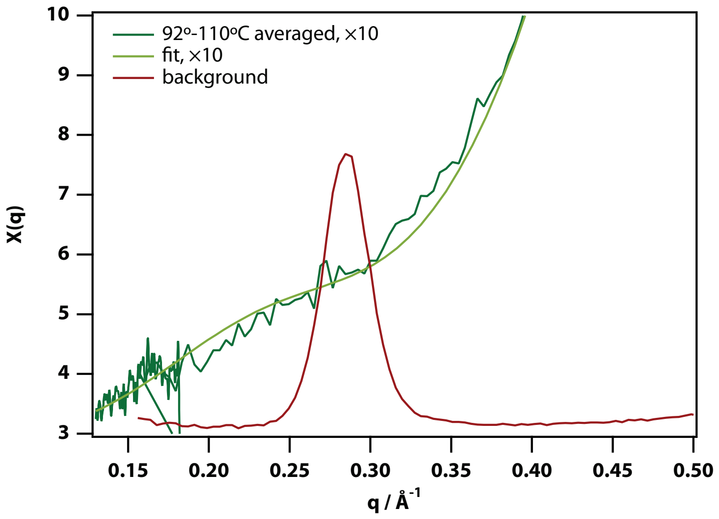

2c. Suppl. Fig. 8 needs some additional care. It would be helpful to make the figure labels visible, include proper legends and also re-bin (using larger q-bins) the SAXS data to improve the readability of the data in the q-range above 0.01/Å.

Supplementary Figure 8 has been improved with bigger and more informative labels, and a larger size. Because of the large amount of data (103 temperatures for each WAXS and SAXS), we have not been able to re-bin (it appears that DAWN only allows re-binning of one data set at a time) or re-colour in a better way. Of course, the raw data will be made available in a public data repository on publication.

3. In connection to the previous comment, I am also wondering about the pre-peak observed S(q) simulations results shown in Fig. 4b and Suppl. Fig. 14:

3a. While indeed as it is pointed out in the paper, the pre-peak is reproduced in the Si-Si S(q), similar peaks in the region q<1/Å with even stronger temperature dependence are shown in the S(q)'s of C-O, C-Si and also in the O-O. Do these temperature-dependent signals reflect changes intermolecular or intramolecular distances? I.e. it should be specified whether in the calculation is included atoms within the same molecule or only intermolecular distances.

Indeed, by definition, the Si-Si $S(q)$ exclusively contains information about the inter-molecular distances (as we have one Si atom / TBOS molecule). As such, it is reasonable to expect the emergence of similar pre-peaks in the other $S(q)$ as well, given that we did include both intra- and inter-molecular contributions in the computation of the $S(q)$. To highlight this aspect, we have now computed, for the O-O case, an $S(q)$ obtained from the PCF relative to the O-O inter-molecular contributions only. It can be seen from Figure R1 (which we have also added to the Supplementary Information) that the $S(q)$ (relative to the O-O intermolecular contributions only) resembles (as it should) the Si-Si $S(q)$ and similarly displays the emergence of a pre-peak. We have also specified in the revised version of the manuscript that our PCFs and thus $S(q)$ have all been obtained by considering all (*i.e.*, inter- as well as intra-molecular) contributions.

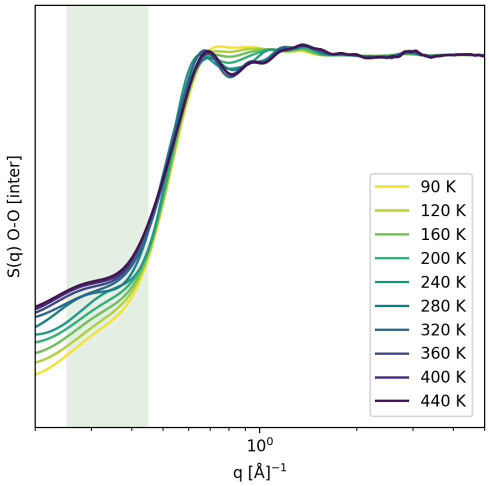

Figure R1. *The structure factor (from MD simulations) relative to the inter-molecular O-O distances only, as a function of temperature. The shaded region highlights the emergence of the pre-peak discussed in the main text.*

> *3b. It might be helpful to plot all these results in Suppl. Fig. 14 with logarithmic x-axis (as in Fig. 4b).*

We agree and have updated Supplementary Figure 14 (now 17) in the revised version of the manuscript as suggested.

> *3c. The peak at q~0.2 /Å is attributed to clusters with size ~2π/q = 26.9 Å. Would then one not expect a similar peak in the Si-Si PCF at this range (Suppl. Fig.15)? Or is this range limited by the simulation box size?*

Thank you for raising this important point. The size of the simulation box (~65 Å at 90 K) is sufficient for us to observe the emergence of these clusters. As illustrated in Figure R2(a), these structural features emerge between 0.2 and 0.4 $Å^{-1}$ in our simulated Si-Si $S(q)$ (here, we focus on Si alone as its structural signatures are the most representative of the topology of the TBOS network), which translates to a real-space range between 14 and 25 Å. The signature in the Si-Si PCF is present—the structure factor is basically the Fourier transform of the PCF after all— and it is highlighted in Figure R2(b). To be sure that the emergence of the pre-peak in our simulations is genuine:

- We have thoroughly investigated the influence of sampling (*i.e.*, how many frames we considered within a certain time interval) and binning (*i.e.*, the "resolution" of the PCF), and found our results to be 100% robust in that respect.
- We have invested a significant amount of computational time to generate three additional, statistically independent models of TBOS, following the same computational protocol discussed in the main text. The total as well as the Si-Si $S(q)$, computed as a function of temperature for the four models is reported in Figure R2(a). Note that, in addition to the (expected) variability of the $S(q)$ as a whole (on our scale we are bound to generate/observe an ensemble of slightly different amorphous systems) the pre-peak is present in all the four models.

As such, we are now entirely confident in claiming that the emergence of the pre-peak is indeed captured by our molecular dynamics simulations. We have now added these data into the Supplementary Information and we have highlighted the results of the additional models in the main text as well.

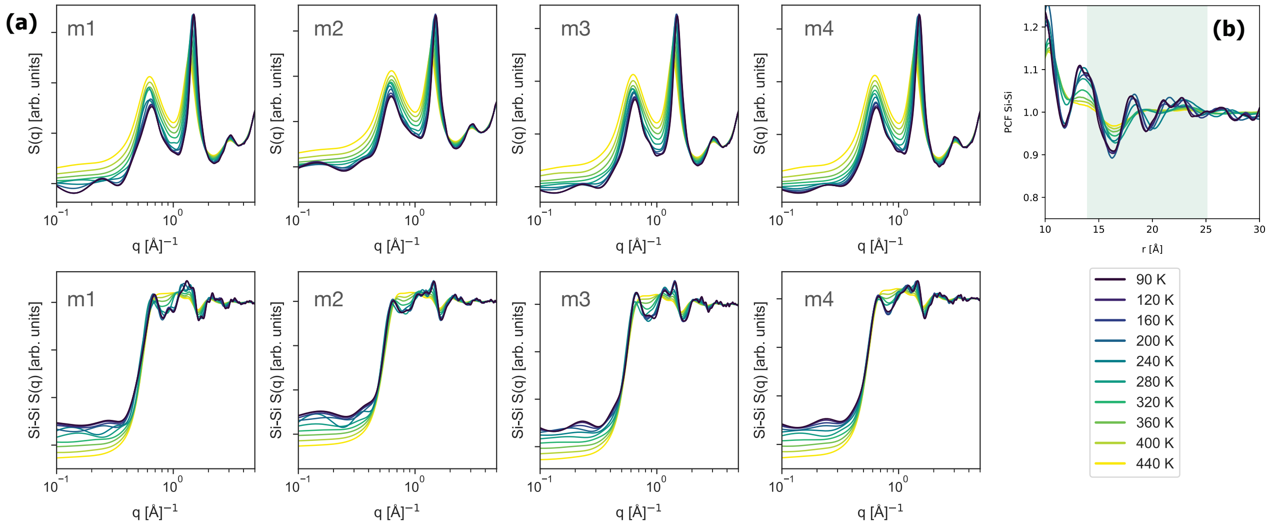

Figure R2. *(a) Total and Si-Si structure factor* S(q) *as a function of temperature for four, statistically independent models of TBOS. (b) Si-Si pair correlation function (PCF) as a function of temperature. The shaded region corresponds to the 0.25-0.45 Å^{-1} region in reciprocal space.*

4. Concerning the Voronoi analysis:

4a. It would be helpful to include some additional explanations concerning the formalism used in the corresponding indices and how one can derive the corresponding symmetry.

Thank you for bringing this matter to our attention. In revising our results, we realised that, in the original version of the manuscript, we inaccurately labelled the indices of some of the Voronoi polyhedra. This is because, upon removing the small faces (*i.e.*, faces with area < 1% of the total area of the Voronoi polyhedron in question) from our Voronoi polyhedra, voro++ (the library we originally used for this calculation) does not update the number of edges and/or vertices for each face. As the Schläfli notation we have used to identify the Voronoi polyhedra consists in a tuple of eight indices (<n_3, n_4, n_5, n_6, n_7, n_8, n_9, n_{10}> as discussed in the Supplementary Note 3), where n_i refers to the number of faces with *i* vertexes/edges, the labelling of the Voronoi polyhedra was not guaranteed to be correct.

The solution to this issue is to exclude the neighbouring particles responsible for the emergence of the small faces and to re-compute the Voronoi polyhedra in their absence. We have now implemented this strategy, and we have also cross-checked the robustness of our results by utilising a second piece of software, Ovito.[1] As such, we have updated Figure 5(c) to reflect the new/correct Voronoi indices.

We stress that the only result that has been impacted by this pitfall is the Voronoi indices: every other feature of the Voronoi polyhedra remains unchanged.

In terms of the relationship between the Voronoi indices and the symmetry of the corresponding Voronoi polyhedra, we have now compiled a rather comprehensive list, summarised in the table below (which has also been added to the revised version of the Supplementary Information). In addition to the corresponding coordination number (CN) for each Voronoi index tuple, we have grouped the different Voronoi polyhedra according to the short-range order they have been reported to be indicative of.

Voronoi index	CN	Notes
Icosahedral (ICO)		
< 0, 0, 12, 0 >	12	Icosahedron
< 0, 1, 10, 2 >	13	Defective icosahedron [2]
< 0, 2, 8, 1 >	11	Z11 Kasper polyhedron [3]
< 0, 2, 8, 2 >	12	Ref.[4]
< 0, 2, 8, 3 >	13	Ref.[5]
< 0, 1, 8, 3 >	12	Ref.[6]
< 0, 0, 10, 2 >	12	Ref. [6]
< 0, 3, 6, 3 >	12	Ref.[7]
< 0, 4, 4, 3 >	11	Based on < 0, 2, 8, 1 >, with defects in polytetrahedral packing [5]

< 0, 4, 4, 4 >	12	Ref.[7]
< 0, 4, 4, 5 >	13	Ref.[5]
FCC		
< 0, 12, 0, 0 >	12	Rhombic dodecahedron
< 0, 3, 6, 4 > [FCC-ICO]	13	Z13 Kasper polyhedron [2]<br>Occasionally labelled as indicative of ICO topology as well [8]
< 0, 3, 6, 5 >	14	Z14 Frank-Kasper polyhedron[5]
< 0, 4, 4, 7 >	15	Ref. [7]
BCC		
< 0, 6, 8, 0 >	14	Truncated octahedron
< 0, 2, 8, 4 >	14	Frank-Kasper polyhedron. Ref. [7]
< 0, 4, 4, 6 >	14	Z14 Kasper polyhedron. Distorted body-centred cubic crystalline clusters [7]
< 0, 5, 2, 6 >	13	Distorted body-centred cubic crystalline clusters [9]
HCP		
< 0, 12, 0, 0 >	12	Trapezo-rhombic dodecahedron
< 0, 4, 4, 2 >	10	Ref. [10]
Prism		
< 0, 3, 6, 0 >	9	Tri-capped trigonal prism (TTP) [11]
< 0, 2, 8, 0 >	10	Bi-capped square Archimedean antiprism (BSAP) [11]
< 0, 4, 4, 0 >	8	Tetragonal dodecahedron [11]
< 0, 4, 4, 1 >	9	Distorted TTP and BSAP [5]
< 0, 3, 6, 1 >	10	Distorted TTP and BSAP [5]
Frank-Kasper[12]		
< 0, 2, 8, 5 >	15	Ref. [5]
< 0, 3, 6, 6 >	15	Ref. [5]
< 0, 4, 5, 4, 1 >	15	Ref. [5]
< 0, 3, 7, 4, 1 >	15	Ref. [5]
Mix		
< 0, 3, 6, 2 >	11	Based on < 0, 2, 8, 1 >, with defects in polytetrahedral packing [5]
Frequently observed in TBOS		
< 0, 2, 5, 4 >	11	Relevant to Al-Cu alloys [6]
< 0, 4, 4, 3, 3 >	14	
< 0, 4, 6, 4, 2 >	16	
< 0, 4, 6, 2, 2, 1 >	15	

4b. It would be interesting to include a schematic of the Si-SI network and polyhedral derived from the simulations to facilitate the interpretation.

We have tried to follow this suggestion, but the very disordered nature of the Si-Si network within TBOS is such that any meaningful attempt to visualise its topology results in a rather messy/uninformative picture (see Figure R3). We hope that the additional information/discussion about the short-range order of the Si-Si network highlighted in the next point (point 4c – see the answers to Reviewer #2 as well, for additional structural/orientation analysis) would serve to provide a clearer picture of the complex topology of this system.

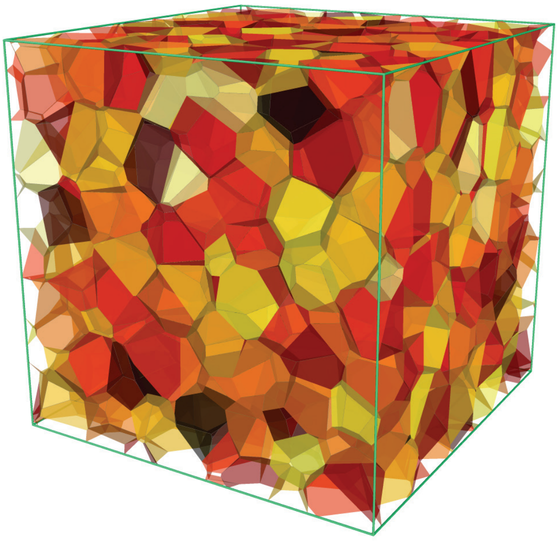

Figure R3. *A representative snapshot of a 512-molecule model of TBOS at 90 K. The network of the Voronoi polyhedra is coloured according to the number of faces of the polyhedra (or, equivalently, the coordination number of the corresponding Si atom). The lighter the colour, the higher the coordination number. Note the remarkable variety of topologies displayed by this truly disordered system.*

> *4c. The analysis indicates that network of the Si atoms exhibits icosahedral coordination at any given temperature, whereas the observed truncated octahedra emerge near the T_g. One of the main conclusions of the paper is however that the observed transient clusters can inhibit crystallization. According to this interpretation, wouldn't one expect that the cluster formation should emerge in the supercooled regime?*

In light of the revised indices for some of our Voronoi polyhedra (see point 4a), we had to reconsider our original claim regarding the emergence of icosahedral and BCC order. As can be seen from Figure R4(a), Voronoi polyhedra characteristic of icosahedral order (<0,1,10,2>, <0,2,8,3> and <0,4,4,5>) are very scarce in the supercooled liquid and become basically absent in the glass. The same can be said for (defective) FCC (<0,3,6,4>) and BCC (0,2,8,4) polyhedra as well, whose population decreases as we approach the T_g. On the other hand, the fraction of (defective) HCP polyhedra (<0,4,4,2>) appears to increase just below T_g, albeit it sharply decreases to a negligible proportion at ~120 K. We note that the emergence, below T_g, of the over-coordinated molecules (<0,4,4,3,3>, <0,4,6,4,2> and <0,4,6,2,2,1>) we observed in the original version of the manuscript, persists even in light of our revised analysis.

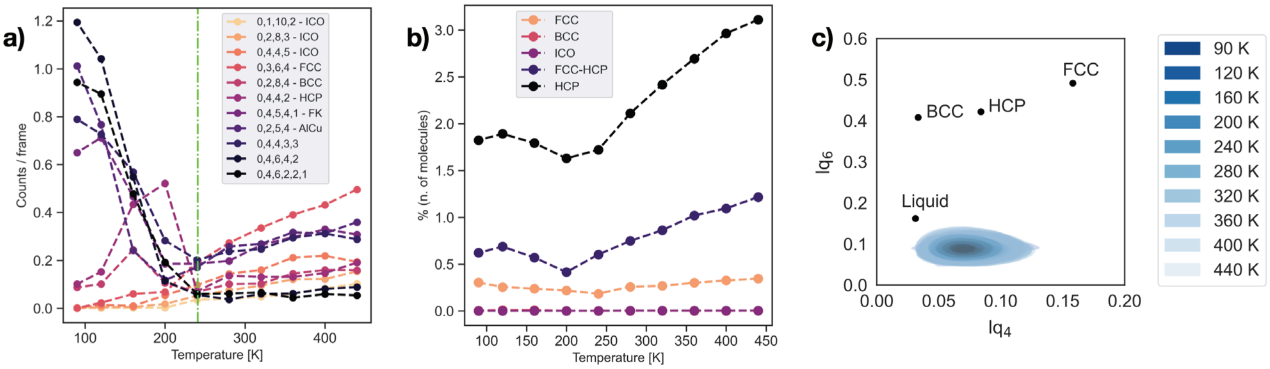

Figure R4. *(a) Frequency of selected Voronoi polyhedra as a function of temperature. The green dashed line indicated the (computationally obtained) T_g. (b) Percentage of TBOS molecules characterised by specific crystalline order, as a function of temperature, obtained according to the framework described in Ref.* [13] *(c) Steinhardt order parameters q_4 and q_6 (computed according to the local average put forward in Ref.* [14] *for each Si atom in the system, as a function of temperature. All the results reported in this figure have been averaged over 1000, statistically independent frames within 10 ns long MD trajectories.*

Whilst it is true that, even in metallic glasses (characterised by a much simpler topology if compared to TBOS), the fraction of crystalline-like Voronoi polyhedra is usually minimal (1-5 %, see *e.g*. Ref. [15]), the variety of Voronoi

polyhedra we observed for TBOS (~10^3) is such that we can only rely on averaged information, such as the number of faces and the volume of the polyhedra (see Figure 5a and 5b), to investigate statistically significant trends in terms of the topology of TBOS as a function of temperature.

To this end, we have leveraged the framework of Ref. [13] to investigate the local molecular environments of the Si network within our TBOS models. The results are summarised in Figure R4(b), and clearly exclude the emergence of either FCC or BCC structures at any temperature. Conversely, a small fraction (~3%) of TBOS molecules sit in HCP environments, whose abundance decreases as the system is cooled below the glass transition.

Further evidence for the absence of substantial crystalline order is provided by the bond order analysis illustrated in Figure R4(c), where we monitor the values of the Steinhardt parameters q_4 and q_6 as a function of temperatures (again, for each Si atom within the TBOS network). We remark that we have employed the local average version of q_4 and q_6 pioneered in Ref. [14]. It can be seen that the system is characterised by values of q_4 that are characteristic of HCP order. However, the fact that the values of q_6 are consistently within the liquid regime is further proof of the fact that this tendency toward the HCP structure is entirely frustrated by the emergence of over-coordinated environments, which add even more disorder into the network. Note that the supercooled liquid is in fact more consistent with HCP order (in terms of q_4) than the glassy state, which is entirely consistent with the local environment analysis of Figure R4(b) (and, to an extent, with the temperature trend of the frequency of the HCP-like <0,4,4,2> as well).

In light of all of the above, we have modified the discussion in the main text accordingly and replaced Fig. 5a with Figure R4(a). Figure R4(b) and (c) have been added in the Supplementary Information together with the corresponding methodological details.

Reviewer #2 (Remarks to the Author):

The work by González-Jiménez studied the dynamical properties, i.e. boson peak, of a molecular glass by combining experiments and simulations. A link to the local structure is suggested. However, this paper is more technical relevant with lots of assumptions involved in the analysis, so it is not convincing to me. Therefore, I cannot recommend its publication in Nat. Commun., a more specialized journal may be more suitable. See some detailed comments below.

1) The introduction should be carefully re-organized to target the problem to be solved in this paper. For example, the main results are related to boson peak and glass structure, but the introduction mainly focused on different relaxation modes, like beta relaxation. Not many details about the boson peak are shown.

We argue in the introduction that both secondary relaxations (Johari–Goldstein ones at least) and the boson peak are related to intermolecular interactions and therefore the intermolecular structure and dynamics. In our view that merits the one paragraph on secondary relaxations in the introduction. (The absence of) β relaxations in the OKE spectra are discussed again in the Discussion and conclusions, so it is important to keep that introductory paragraph.

To strengthen the paragraph on the boson peak, we have added references to the recent paper on the origin of the boson peak in Nature Physics (2022), which is also discussed briefly in the conclusion section. In addition, we have added a reference to the recent (2022) book "Low-Temperature Thermal and Vibrational Properties of Disordered Solids" by Miguel A. Ramos. Finally, a couple of sentences have been added regarding the VDOS in glasses *vs*. crystals.

2) In the introduction, "Below the glass-transition, secondary or β-relaxation processes occur due to faster dynamic processes" may not be necessarily true. I believe different relaxation modes should coexist always in a thermodynamic state, but their observation will depend on the experimental time scale.

We agree on this but feel that a discussion on observation timescales is not appropriate for this manuscript on the boson peak. However, the word "occur" does indeed suggest that β relaxations only come into existence below T_g, which is not necessarily correct. Therefore, we have modified the phrasing in that paragraph.

3) There is a contradiction about the origin of the secondary relaxations in the introduction, whether intramolecular or intermolecular.

There is no contradiction: β relaxations can be intermolecular or intramolecular and which one is dominant depends on the system. In polymers, intramolecular (small angle orientational diffusion of the side chains or functional groups) β relaxations are thought to be dominant whereas in small-molecule and metallic glasses intermolecular/atomic β relaxations (or Johari–Goldstein) are dominant. Since this manuscript is on a small-molecule glass former, the discussion in this paragraph is biased towards Johari–Goldstein β relaxation.

> *4) The detailed data analysis from the experimental spectra depends on the assumption that they will be simplified for some molecules with ordinary symmetry, tetrahedral and octahedral. This has not been rationalized. Also, in the glass state, there are enormous types of these local structures with different distortions, how would this fact influence the results?*

The fact that the depolarised Raman spectrum depends on the anisotropic part of the molecular polarisability tensor is in basic textbooks on Raman scattering while the vanishing of the anisotropic part in molecules with tetrahedral symmetry is basic group theory. However, it is of course correct that a molecule such as TBOS will be distorted and will not have perfect tetrahedral symmetry. The effect of this distortion is discussed in detail on page 3 (plus more details in the Methods section) where we discuss DFT calculations of the molecular polarisability tensor for 31,500 low-energy conformers.

> *5) The spectrum fitting with four functions of many free parameters seems highly possible to bring over-fitting. This is very critical for the overall interpretation of the results.*

This is of course correct in principle. However, TBOS was specifically chosen as its near-tetrahedral symmetry simplifies the spectra. As a result, the spectra taken in the glassy state (Figure 1(c)) clearly show 3 peaks below 4 THz, making fitting very straightforward and unambiguous. Well above the glass transition, these very same 3 peaks can still be readily observed, see, for example, Supplementary Figure 5, 300 K. At still higher temperatures, the diffusive α relaxation band shifts to ever higher frequencies, making fitting trickier. However, as Supplementary Figure 7 shows, the fitted relaxation time-constant closely follows the Stokes-Einstein law using the viscosity measured with rheometry as expected. Therefore, one can have full confidence in these fits.

> *6) The structure analysis by pair-correlation function and the Voronoi tessellation does not account for the orientation information in the local structures, which should be important in this molecular glass.*

TBOS molecules can be considered as approximately tetrahedral units. The first question in terms of the orientation of these tetrahedral units is whether, on average, they would assume any preferential orientation with respect to a given reference axis, particularly in the glassy state. To investigate this possibility, we have now computed, for each TBOS molecule, the angle Θ_z formed by each of the four Si-O molecular axes (defined as the vectors originating from the Si atom and pointing toward each of the four oxygen atoms) and the Cartesian z-axis. We have then averaged these four angles over all the molecules within a given frame to obtain an average orientation $\langle\Theta\rangle_z$. The probability density function of $\langle\Theta\rangle_z$, computed over 1000 statistically independent frames within 10 ns long trajectories, is reported in Figure R5(a) as a function of temperature. It can be seen that the distributions peak at ~90° at every temperature, which demonstrates that the system is isotropic even in the glassy state. Note the narrowing of the distribution as the temperature decreases – which is indicative of the much slower rotational dynamics within the glassy state.

Next, we wanted to investigate the relative orientation of the TBOS tetrahedral units within their local environments. To this end, we define, for each molecule, the plane illustrated in Figure R4(b). We then leverage the so-called Solid Molecule Angle Criteria (SMAC) order parameter, described in Ref. [16]. In a nutshell, SMAC measure the degree to which molecules, within a certain distance from each other, are similarly oriented. In this case, we considered up to the 2nd coordination shell of the TBOS molecules, which encompasses ~12 neighbours. The orientation of each molecule is defined by the vector normal to the plane illustrated in Figure R4(b). Higher (lower) values of SMAC correspond to more (less) ordered molecular environment in terms of the relative orientation of, in this case, the tetrahedral SMAC units.

The results are summarised in Figure R4(c), where we have averaged the value of SMAC for each molecule within a given frame (which yields <SMAC>) and then built a probability density function over different frames (same sampling as Figure R4(a)). Interestingly, it appears that the glassy state is less ordered, in terms of the relative orientation of the TBOS molecules, than the supercooled liquid. This result is consistent with the progressive loss

of short-range order we have observed via the analysis reported in Figure R3, and provides further evidence of the fact that the over-coordinated (Voronoi polyhedra characterised by > 12 faces, particularly 15 and 16 faces) clusters of TBOS molecules we have observed in the glassy state are not characterised by any degree of crystalline order.

This analysis has now been added to the Supplementary Information.

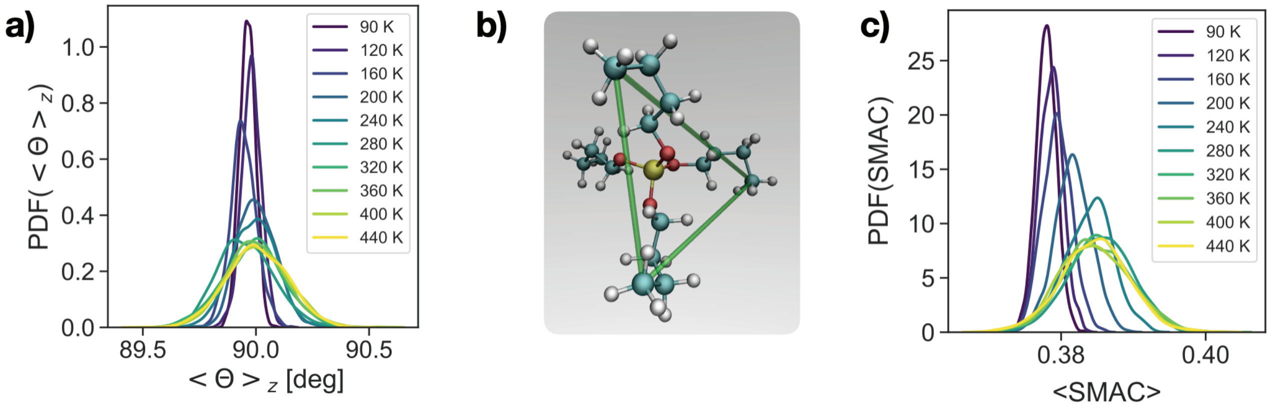

Figure R5. *(a) probability density function (PDF) of the average value of the angle Θ_z (see text) as a function of temperature. The value of Θ_z for an entirely random average orientation of the molecular axis is 90°. (b) Definition of the molecular plane (green lines) identifying the orientation of a TBOS molecule / tetrahedral unit. (c) PDF of the average value of the SMAC order parameter (see text) as a function of temperature. All the results reported in this figure have been averaged over 1000, statistically independent frames within 10-ns long MD trajectories.*

> *7) The direct comparison of the simulations by a simplified model with the experiments is not convincing. The consistency of the partial structure factor only shows the overall averaged information similarly, not necessarily mean the model it's correct.*

The MD simulations accurately reproduce: (1) the temperature-dependent behaviour of the OKE spectra; (2) the temperature-dependent behaviour of the WAXS data, including the emergence of the pre-peak at ~0.2 Å^{-1}; (3) the temperature-dependent changes of the relative population of gauche/trans configurations in the alkoxide chains of TBOS—an additional piece of analysis we have now included in the Supplementary Information, see our answer to Reviewer #3. As such, the agreement between simulations and experiments is remarkable, especially if we consider that the force field we have utilised was not explicitly parametrised with respect to alkoxides such as TBOS. In addition to this, we have now invested a substantial amount of computational time to generate three additional, statistically independent models of TBOS, which allowed us to strengthen even further our claims in terms of the structural features we observe in our molecular dynamics simulations (see the answer to Reviewer #1, point 3c).

> *8) At elevated temperatures, anharmonicity is important, which should have been into consideration in the spectrum analysis.*

Our simulated OKE spectra, which—because of the inherent symmetry of the TBOS molecules are directly related to the translational motion of Si atoms—have been obtained from the Fourier transform of the Si-Si velocity autocorrelation functions, *do* take into account anharmonicity, given that our MD simulations have been performed at finite temperature.

Reviewer #3 (Remarks to the Author):

> *The manuscript of González-Jiménez et al. entitled "Understanding the emergence of the boson peak in molecular glasses" aims at unveiling the boson peak in a highly symmetric glass former using femtosecond optical Kerr-effect spectroscopy as the central characterization technique, at different temperatures. They then use other experimental techniques (WAXS, SAXS, Raman) and simulation to characterize this boson peak. It is because the characterized molecule, TBOS, is symmetric that the authors can tackle this longstanding problem of the origin of the boson peak.*
>
> *Two intermolecular bands in the OKE spectrum have been mainly analyzed. In Figure 1 d, the amplitudes of both bands are shown versus the temperature, and in Figure 2 a, their*

ratio is displayed. Through theses analyses, the authors assign the low frequency band as the boson peak, and the high frequency band as the acoustic phonon band. Thanks to this assignment, they surmised that the boson peak is associated with the formation of clusters of TBOS molecules undergoing collective oscillation. WAXS experiments lead to a distance of 26.9 Angstrom for clusters, and from MD simulation, 25.1 Angstroms. Conclusions drawn from the OKE experiments can have been made because of the symmetric nature of the TBOS molecule. Regarding the over-coordinated molecules in the clusters, deduced from MD, the authors wrote: "the MD simulations are strongly suggestive that these structures are clusters of over-coordinated TBOS molecules". For an article in Nature Communications, the authors must be more convincing. For instance, as the molecular behavior can be probed, the over-coordination can be proved.

We have in fact conclusive evidence, from our molecular dynamics simulations, of the emergence of over-coordinated TBOS molecules – as proven by the Voronoi analysis reported in Fig. 5a. We also have conclusive evidence for the fact that these over-coordinated molecules tend to cluster in aggregates whose size increases as the system is cooled into the glassy state. This was mentioned, briefly in the original version of the manuscript, but we have now included the probability density function of the largest cluster (consisting of TBOS molecules characterised by Voronoi polyhedra with 15 or more faces) in each frame of our MD trajectories in the Supplementary Information. The result is summarised in Figure R6 as well, as a function of temperature. Finally, we have shown (Fig. 5(d)) that over-coordinated TBOS molecules (particularly those characterised by Voronoi polyhedra with 16 faces) are responsible for populating the high-frequency region of the Fourier Transform of the velocity-velocity autocorrelation function of the Si atoms (a quantity which is directly related to the experimental OKE spectrum given the symmetry of the TBOS molecules). In light of all of the above, we are quite confident that these particular structural features do correspond to those identified by the OKE measurements. As such, we have replaced the rather cautious wording, "strongly suggestive" with "show" in the revised version of the manuscript.

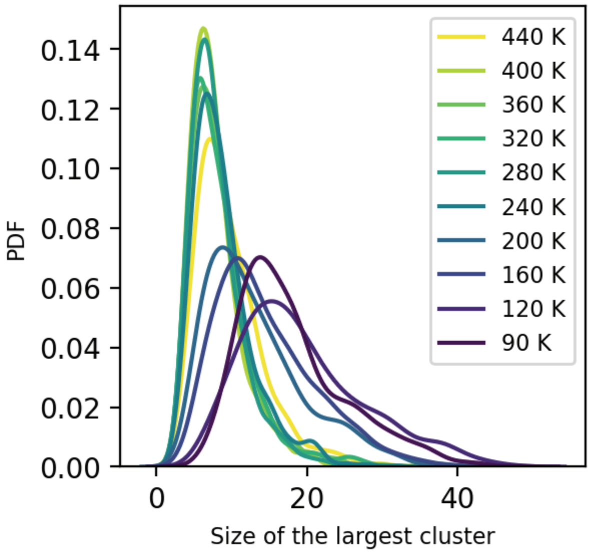

Figure R6. *Probability function of the size of the largest cluster of TBOS molecules characterised by Voronoi polyhedra with 15 or more faces, as a function of temperature.*

Other issues.

p.3: It is written "The calculated infrared spectrum in the Si-O-C stretch region (around 1,100 cm-1) matches the experimental one (see Supplementary Figure 3)". Only the 1000-1200 cm-1 spectrum should be considered. A great part of the simulated spectrum does not fit the experimental one. The authors should indicate why the fit works only for the region of interest.

Our apologies! In the original manuscript the computed spectrum was simply shifted and therefore appears (if you look at the peak wavenumbers rather than the shape) to be completely mismatched. It is common for normal mode calculations to slightly overestimate the normal-mode frequencies. Therefore, we have matched the calculated frequencies of the CH-stretch modes with the experimental ones by multiplying them with 0.936. This shifts the

calculated Si-O-C modes down a bit as well and the correspondence is now much more obvious. The figure and its caption have been updated.

The figures below show the spectra from 675 cm^{-1} (the lowest we have been able to go experimentally with the available IR windows) to 3,000 cm^{-1}. It can be seen that the correspondence is reasonable over the entire range. The manuscript only shows the 1,000-1,200 cm^{-1} region as this should show a prominent peak in highly symmetrically coordinated (tetrahedral, octahedral) alkoxides, as it does in TBOS.

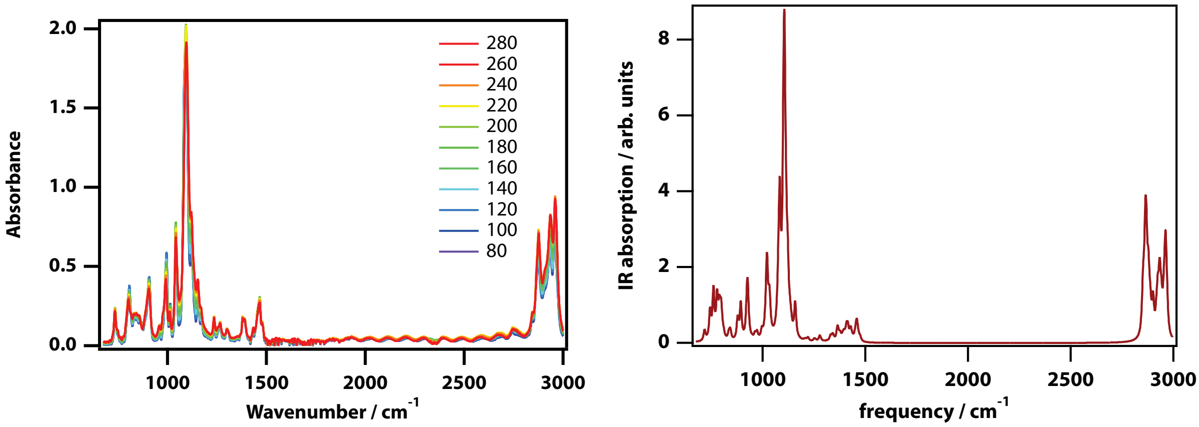

Figure R7. ***Experimental (left) and calculated normal-mode spectra (right) of TBOS.***

> *p.4: the Supplementary Figure 9 does not correspond to the liquid density as outlined in the text.*

We are not sure what the reviewer means by this as the density is not mentioned in the text except for the caption of Supplementary Figure 9.

> *Indicate what are q3 and q5.*

We apologise that several parameters were not defined in the manuscript! Changes were made to Supplementary note 2 (Analysis of WAXS data) and the captions/headers of Supplementary Tables 2 and 3. All the amplitude (L_i and G_i), width (γ_i and σ_i), and position (q_i) parameters are now properly defined and the definitions referred to in the text.

> *Below 110 K: the value seems to be 110 K.*

Agreed, changed to "at".

> *It is written that "the TBOS molecules reduce the number of gauche defects in the alkoxide side chains". Such a behaviour should be observed in MD.*

Indeed, this behaviour is captured by the MD simulations. To illustrate this aspect, we have computed the probability density function of the value of all the H-C-C-H dihedral angles (ϕ) within the alkoxide side chains, averaged across 40 ns at each temperature. The results are summarised in Figure R8. As the temperature decreases, the distribution of ϕ narrows (as the mobility of the side chains is greatly reduced in the glassy state) and we observe a reduction of the relative population of gauche configurations with respect to trans configurations (as demonstrated by the ratio between the height of the PDF peaks at ~120° [trans] and ~60° [gauche], which increases from ~2.5 at 440 K to ~3.4 at 90 K). Trans configurations are clearly more energetically favoured at any temperature, potentially due to steric effects. Figure R8 has now been added to the Supplementary Information.

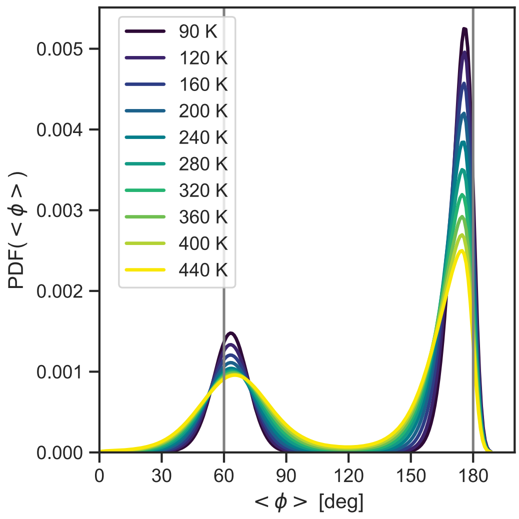

Figure R8. *Probability density function (PDF) of the averaged values of the H-C-C-H dihedral angles within the alkoxide side chains of TBOS molecules as a function of temperature. The two grey lines at $\phi = 60°$ and 120° indicate typical values of gauche and trans configurations, respectively.*

> *p.5: The authors consider the two coordination shells in the RDF, computing 2 and 12 molecules respectively. According to these spectra, these numbers should change with the temperature. Some relevant results can be obtained.*

Indeed, the Si-Si coordination number of the system does change with temperature, as illustrated in Fig. 4d. One aspect we failed to stress in the original version of the manuscript is that the short-/medium-range order in supercooled liquid, and even more so in glassy TBOS, is rather peculiar. In fact, we observe a first Si-Si coordination shell (up to 7.5 Å) that on average includes only ~2.5 nearest neighbours. This is interesting, as even in the glassy state the TBOS molecules can almost never quite arrange themselves into a 4-coordinated configuration within their first coordination shell. Instead, they either assume a 2-fold or at most a 3-fold coordinated configuration, illustrated in Figure R9, where the TBOS molecules are almost lying in the same plane. We argue that these peculiar structural features have to do with steric effects related to the presence of the alkoxide chains (on top of the fact that tetrahedra do not fill 3D space), which prevents a denser packing of the system. Rather distinct from this under-coordinated short-range feature, we also observe a second Si-Si coordination shell (which extends up to ~12 Å) that includes, on average, ~12 nearest neighbours (consistently with our Voronoi analysis, see *e.g.* Fig. 5a). We have now highlighted this structural feature of the system in the revised version of the manuscript.

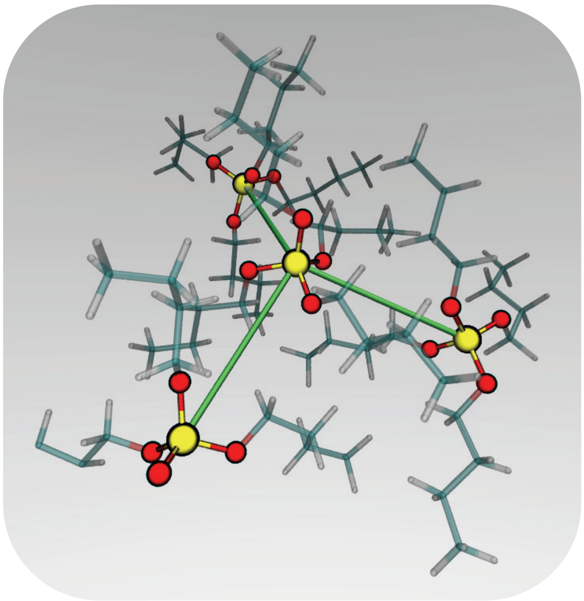

Figure R9. *A representative example of a 3-fold coordinated TBOS molecule*

p.9: MD simulation part.

When the authors consider random positions within a cubic box: what is the random algorithm used? It is necessary to specify that the periodic boundary conditions have been applied.

We have used Packmol[17] to generate the initial random arrangement of TBOS molecules within the simulation box. Details about the algorithm used by this particular piece of software can be found in Ref. [18]. We have added this information in the Methods section. However, we note that, given we have thoroughly equilibrated the system in a highly diffusive regime, the initial (random) configuration of the TBOS molecules within the simulation box does not have any impact on our subsequent molecular dynamics simulations.

Simulations are performed in the NPT ensemble, meaning that a thermostat and a barostat are needed simultaneously. So, why the authors wrote: "to sample the canonical ensemble"?

Thank you for pointing this out. We have now changed this sentence: "*The Bussi-Donadio-Parrinello thermostat[49] was used to sample the canonical ensemble, in conjunction with the Berendsen barostat.[50]*" to: "*The Bussi-Donadio-Parrinello thermostat[49] was used in conjunction with the Berendsen barostat[50] to sample the isobaric-isothermal (NPT) ensemble.*"

The authors wrote: "In this regime, the self-diffusion is sufficient to sample a reasonable portion of the configurational space for the liquid phase". One point in the configurational space corresponds to 87,552 coordinates. What do the authors mean with this reasonable portion?

We apologise for the confusion. We have computed the self-diffusion coefficient of the Si atoms at that temperature to be ~1 nm^2/ns at 440 K, which indicates that the system is in a highly diffusive, hydrodynamic regime (we have verified this by analysing both the mean square displacement and the self part of the intermediate scattering function, which at that temperature displays a single exponential decay characteristic of a purely hydrodynamic regime). As we have equilibrated the system for > 20 ns at that temperature, we are confident that we have generated statistically independent configuration of the liquid to serve as starting points for our quench/cooling protocol.

To make this clearer, we have now changed the sentence: "In this regime, the self-diffusion is sufficient to sample a reasonable portion of the configurational space for the liquid phase, thus providing an adequate starting point for the cooling ramp." to: "Under these conditions, the self-diffusion coefficient of the Si atoms was computed to be ~1 nm^2/ns indicative of a highly diffusive hydrodynamic regime. As the system was equilibrated for a further 20 ns at that temperature and pressure, statistically independent well-equilibrated configurations of the liquid were generated providing an adequate starting point for the cooling ramp." in the revised version of the manuscript.

Finally, how can the study presented in this paper be correlated with the recent paper in Nature Physics on the origin of the boson peak?

Thank you. Of course, this nice paper only came out when this manuscript was submitted. A sentence has been added to the Discussion linking our results to the recent theoretical work by Hu and Tanaka in Nat. Phys.

References

1. Stukowski, A. Visualization and analysis of atomistic simulation data with OVITO–the Open Visualization Tool. *Model. Simul. Mater. Sci. Eng.* **18**, 015012 (2009).
2. Mizuguchi, T., Tatsumi, S. & Fujiwara, S. Icosahedral order in liquid and glassy phases of cyclohexane. *Mol. Simul.* **46**, 721–726 (2020).
3. Sheng, H. W., Luo, W. K., Alamgir, F. M., Bai, J. M. & Ma, E. Atomic packing and short-to-medium-range order in metallic glasses. *Nature* **439**, 419–425 (2006).
4. Guo, G. Quasi-Icosahedral Clusters in Zr-Based Metallic Glasses. *Metals* **10**, 1135 (2020).
5. Zhang, H., Sun, H., Li, Q. & Wang, L. Origin of Local Structures of U-Co Melts: A First-Principles Study. *Front. Mater.* **8**, (2022).
6. Zhang, Q., Li, J., Tang, S., Wang, Z. & Wang, J. Atomistic Mechanism Underlying Nucleation in Al–Cu Alloys with Different Compositions and Cooling Rates. *J. Phys. Chem. C* **125**, 3480–3494 (2021).

7. Şengül, S. & Çeltek, M. Pressure Effects on the Structural Evolution of Monatomic Metallic Liquid Hafnium. *Bitlis Eren Üniversitesi Fen Bilim. Derg.* 144–158 (2018) doi:10.17798/bitlisfen.416922.
8. Ren, L., Gao, T., Ma, R., Xie, Q. & Hu, X. The icosahedral short-range order and its local structures in $Cu_{50}Zr_{40}Al_{10}$ alloy. *Mater. Res. Express* **6**, 016510 (2018).
9. Han, J. *et al.* Atomic-Level Mechanisms of Nucleation of Pure Liquid Metals during Rapid Cooling. *ChemPhysChem* **16**, 3916–3927 (2015).
10. Durandurdu, M. MgCu metallic glass. *Philos. Mag.* **98**, 633–645 (2018).
11. Fukunaga, T. *et al.* Voronoi Analysis of the Structure of Ni-Zr-Al Ternary Metallic Glass. *Mater. Trans.* **48**, 1698–1702 (2007).
12. Frank, F. C. & Kasper, J. S. Complex alloy structures regarded as sphere packings. I. Definitions and basic principles. *Acta Crystallogr.* **11**, 184–190 (1958).
13. Mosayebi, M. *et al.* Beyond icosahedral symmetry in packings of proteins in spherical shells. *Proc. Natl. Acad. Sci.* **114**, 9014–9019 (2017).
14. Lechner, W. & Dellago, C. Accurate determination of crystal structures based on averaged local bond order parameters. *J. Chem. Phys.* **129**, 114707 (2008).
15. Yang, L. *et al.* Structural responses of metallic glasses under neutron irradiation. *Sci. Rep.* **7**, 16739 (2017).
16. Giberti, F., Salvalaglio, M., Mazzotti, M. & Parrinello, M. Insight into the nucleation of urea crystals from the melt. *Chem. Eng. Sci.* **121**, 51–59 (2015).
17. Martínez, L., Andrade, R., Birgin, E. G. & Martínez, J. M. PACKMOL: A package for building initial configurations for molecular dynamics simulations. *J. Comput. Chem.* **30**, 2157–2164 (2009).
18. Martínez, J. M. & Martínez, L. Packing optimization for automated generation of complex system's initial configurations for molecular dynamics and docking. *J. Comput. Chem.* **24**, 819–825 (2003).

REVIEWERS' COMMENTS

Reviewer #1 (Remarks to the Author):

I thank the authors for addressing my comments in detail with the revised version. Therefore, I support the paper in its present form for publication at Nature Communications.

Reviewer #2 (Remarks to the Author):

I am glad to see that the authors have carefully addressed my concerns, and the manuscript has been much improved. The paper now can be in principal recommended to publication in Nat Commun., while the following points are required to be addressed.

1. At the beginning of the Results, the authors claim the fragile nature of the liquid based on T_0 < T_g. I cannot agree that the fragility of a material can be identified in that way. As T_0 is the hypothetical dynamical glass transition point, which should always be smaller than T_g, no matter for fragile or strong liquid.
2. In Fig. 1(d), the amplitudes were depicted, and their ratio was shown in Fig. 2(a). The authors claimed that one of them is temperature invariant and the other linearly changes with temperature. Then why their ratio shows so large fluctuation and fits to an exponential function. What is the physical meaning of this function?
3. I have some concerns on the Voronoi analysis. 1) the local polyhedral are usually distorted, a specific order, like ICO, may be associated with many different Voronoi index. Counting a specific Voronoi index may not be enough and accurate. 2) extremely small Voronoi faces can appear which will significantly affect the Voronoi index.

Reviewer #3 (Remarks to the Author):

The authors' responses to my comments are very satisfactory. The new manuscript better meets the expected quality standards.

Reply to reviewers' comments

Reviewer #1 (Remarks to the Author):

I thank the authors for addressing my comments in detail with the revised version. Therefore, I support the paper in its present form for publication at Nature Communications.

Reviewer #2 (Remarks to the Author):

I am glad to see that the authors have carefully addressed my concerns, and the manuscript has been much improved. The paper now can be in principal recommended to publication in Nat Commun., while the following points are required to be addressed.

1. At the beginning of the Results, the authors claim the fragile nature of the liquid based on T_0 < T_g. I cannot agree that the fragility of a material can be identified in that way. As T_0 is the hypothetical dynamical glass transition point, which should always be smaller than T_g, no matter for fragile or strong liquid.

This sentence has now been deleted.

2. In Fig. 1(d), the amplitudes were depicted, and their ratio was shown in Fig. 2(a). The authors claimed that one of them is temperature invariant and the other linearly changes with temperature. Then why their ratio shows so large fluctuation and fits to an exponential function. What is the physical meaning of this function?

The reviewer has probably misinterpreted the caption of Fig. 1, which states that the amplitudes of the **intramolecular** modes are invariant. As clearly discussed in the caption of Fig. 2, the exponential fit is included solely as a **guide the eye** and therefore has no physical meaning.

3. I have some concerns on the Voronoi analysis. 1) the local polyhedral are usually distorted, a specific order, like ICO, may be associated with many different Voronoi index. Counting a specific Voronoi index may not be enough and accurate. 2) extremely small Voronoi faces can appear which will significantly affect the Voronoi index.

These issues were already addressed in detail in the manuscript.

1) It is true that a specific local order (icosahedral, FCC, BCC, *etc*…) may be associated with a variety of different Voronoi indices. This is why we had compiled a comprehensive portfolio of Voronoi indices (11 different ones for icosahedral order alone) for each type of local order, by collating information from the literature. The results of this exercise were reported in Supplementary Table 4.

2) As already discussed in the Supplementary Information (Page 3), "To reduce the complexity of the Voronoi analysis, faces that account for less than 1% of the total surface area of a given VP were ignored—in line with the approach of, *e.g.*, Ref. [9] —aimed at avoiding overcounting and remove too small structural fluctuations."

Reviewer #3 (Remarks to the Author):

The authors' responses to my comments are very satisfactory. The new manuscript better meets the expected quality standards.
